# The third orthogonal dynamic covalent bond†

**DOI:** 10.1039/c6sc01133k

**Published:** 2016-04-20

**Authors:** Inés Lascano, Kang-Da Zhang, Robin Wehlauch, Karl Gademann, Naomi Sakai, Stefan Matile

**Affiliations:** a National Centre of Competence in Research (NCCR), Molecular Systems Engineering (MSE) Switzerland http://www.nccr-mse.ch; b Department of Organic Chemistry, University of Geneva Geneva Switzerland stefan.matile@unige.ch http://www.unige.ch/sciences/chiorg/matile/ +41 22 379 5123 +41 22 379 6523; c Department of Chemistry, University of Zurich Zurich Switzerland

## Abstract

Orthogonal dynamic covalent bonds are of interest for the construction of functional systems. The orthogonality of disulfide and hydrazone exchange under basic and acidic conditions, respectively, is well established. However, the integration of boronate esters as the third bond has failed so far because they exchanged too easily, especially under hydrazone exchange conditions. In this report, a collection of bioinspired catechols derived from adhesive natural products from cyanobacteria is screened with phenylboronic acids with proximal alcohols (benzoboroxoles), amines and fluorines to identify the least labile boronate esters. Moreover, Kool's 2-aminophenol catalysts are introduced to selectively accelerate hydrazone exchange without disturbing sufficiently inert boronate esters. Based on these results, we identified three different conditions to selectively exchange disulfides, hydrazones and boronate esters, that is to demonstrate the existence of three orthogonal dynamic covalent bonds. Moreover, their compatibility with functional systems is confirmed by successful hydrazone exchange in multicomponent surface architectures in the presence of intact boronate esters and disulfides.

## Abstract 2

The existence of three fully orthogonal dynamic covalent bonds is demonstrated in solution and in functional surface architectures.

## Graphical abstract

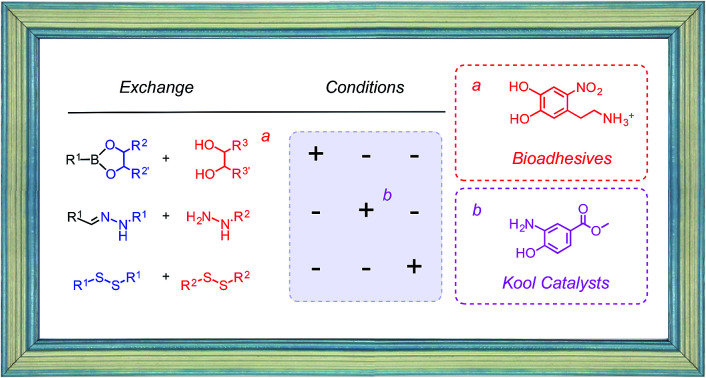

Dynamic covalent bonds are fascinating because they can be either as stable as covalent bonds or as rapidly exchanging as non-covalent bonds, depending on the conditions.^1–4^ This dual nature makes them ideal tools for the construction of multicomponent functional systems. However, contrary to the routine use of several non-covalent bonds at the same time, dynamic covalent bonds are usually used alone. This could be in part due to the poor orthogonality between different types of dynamic covalent bonds. Orthogonality is defined by the existence of conditions that allow the exchange of one without disturbing the others (Fig. 1). The most popular pair of orthogonal dynamic bonds, hydrazones^2^ and disulfides^3^ exchange exclusively under acidic and basic conditions, respectively, and thus enabled the construction and operation of various doubly dynamic functional systems.^5–8^

**Fig. 1:**
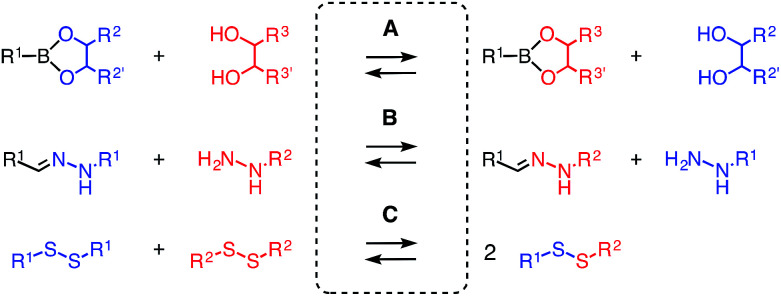
Selective exchange under conditions A, B and C defines orthogonality of disulfides, hydrazones and boronic/boronate esters.

Extending this approach, triply dynamic functional systems have been constructed very recently^9,10^ with boronate esters,^1^ hydrazones and disulfides. These studies have shown the feasibility to exchange boronate esters in the presence of intact hydrazones and disulfides^9^ and to simultaneously form these three types of bonds.^10^ However, the orthogonality of boronate esters as the third dynamic covalent bond could not be established so far because (a) they are too labile, particularly under acidic conditions, and (b) hydrazone exchange requires strong acids.^9,10^ To overcome this dilemma, we first considered the use of electron-deficient catechol derivatives of anachelin, a natural product from the cyanobacterium *Anabaena cylindrica*. The high affinity of these bioinspired catechols to mineral oxides^11^ suggested that they might also afford more inert boronate esters. To overcome the second obstacle *en route* to the third orthogonal organic dynamic covalent bond, weakening of the hydrazone bond was not an option because it would destabilize the entire functional system.^12^ Acceleration of the exchange of inert aryl hydrazones appeared more promising, also because the original aniline catalysts^13^ have been dramatically improved recently with bifunctional acid/base catalysts.^14–16^ The anthranilic acids^15^ and, more interestingly, the 2-aminophenols^16^ are thought to operate by intramolecular protonation of the OH leaving group *via* 8- and 7-membered rings, respectively, to accelerate the rate-limiting dehydration during hydrazone formation from aldehydes and hydrazines. Contemplating this elegant catalyst design, we felt that the proposed mechanism could also extend to the activation of the NH leaving group during hydrazone exchange. In the following, we show that indeed the combination of bioinspired catechol adhesives^11^ and cutting-edge organocatalysts^16^ was the key to find the third orthogonal dynamic covalent bond.

Catechols 1–9 were chosen for this study (Fig. 2) and the protected anachelin chromophore 5^11^ served as starting point for the design of catechols. Recognizing the power of electron-deficient aromatic units, we prepared new catecholate derivatives 2, 7–9 for subsequent experiments. Phenylboronic acids 10–12 with proximal primary alcohols (benzoboroxoles),^17^ tertiary amines^1*c*,18^ and fluorines^7^ have been reported previously. The formation of boronate esters was assessed in MeOH/water 3 : 1, pH 7.8, at room temperature (Fig. S1 and 2†). Changes in the absorption spectra of catechols, kept constant at low micromolar concentrations, were recorded in response to increasing concentrations of boronic acids. Reverse titration of boronate esters of alizarin red 4 provided access to the *K*_D_'s of otherwise “invisible” catechols.

**Fig. 2:**
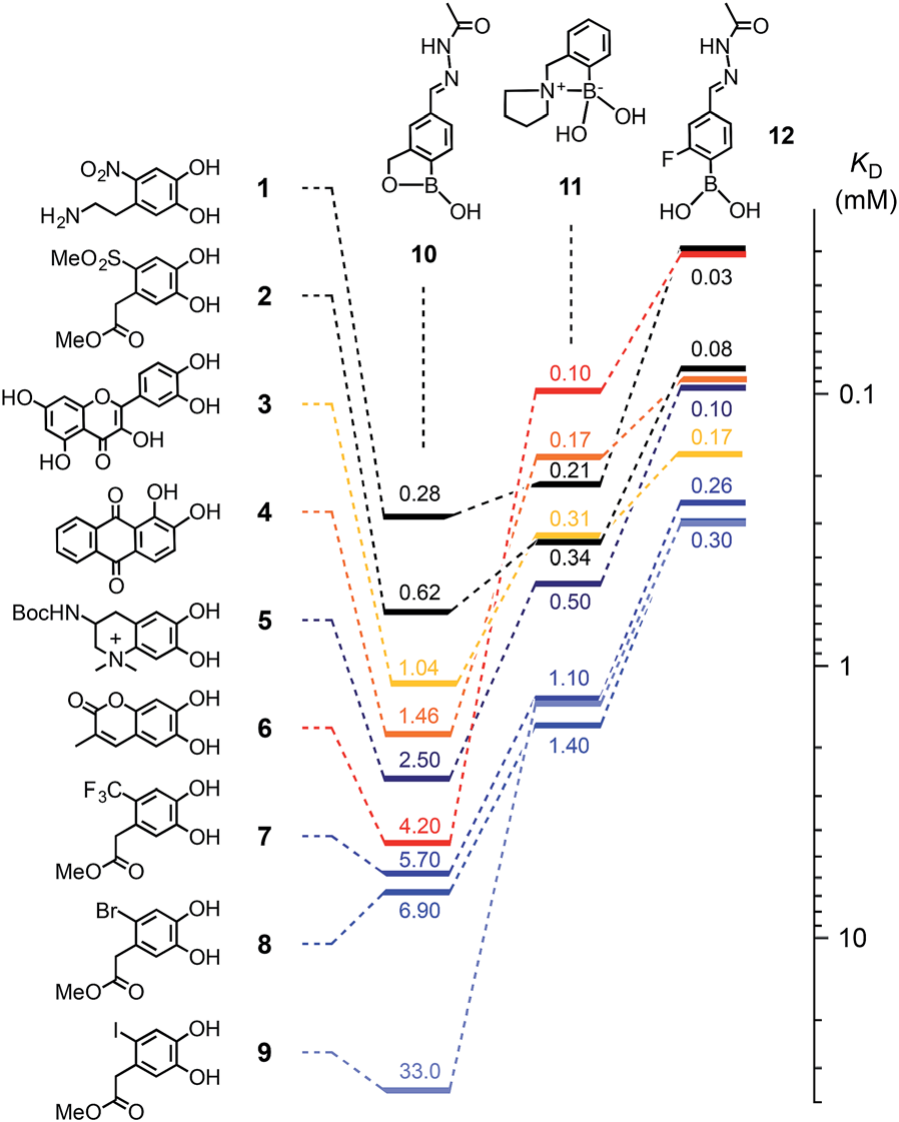
Dissociation constants *K*_D_ (mM) of boronic esters obtained from catechols 1–9 and boronic acids 10–12 in MeOH/H_2_O 3 : 1 (100 mM HEPES, pH 7.8); from changes in absorption upon direct titration (1, 3, 4 and 6) or reverse titration with 4 (2, 5, 7–9).

Compared to the previous best performing catechols 3, 4 and 6, the most distinctive results with the new “bioadhesives” 1, 2, 5 and 7–9 were obtained with benzoboroxoles 10 (Fig. 2, Table S1†). As on oxide surfaces,^11^ nitrodopamine 1 with a *K*_D_ = 280 μM was best also with regard to boronate ester formation. It was followed by the similarly electron-deficient methyl sulfone 2 at 620 μM, quercetin 3 and alizarin red 4 as the first positive controls at 1.04 and 1.46 mM, and the close anachelin mimic 5 at 2.50 mM. “Bioadhesives” 2, 5 and particularly 1 also gave top values with the fluorinated boronic acid 12, whereas boronic acid 11 was overall slightly less convincing with anachelin mimics, particularly 1.

The outstanding *K*_D_'s of the new “bioadhesives” 1 and 2 with benzoboroxole 10 were most important because boronate esters of benzoboroxoles have been identified previously as best in functional systems, presumably because the less electrophilic tetrahedral boronate is intramolecularly stabilized.^9^ Testifying its inertness, the boronate ester 13, prepared from the best performing anachelin mimic 1 and 10, underwent only marginal exchange with catechol 8 to boronate 14 under acidic hydrazone exchange conditions B (around 10%, see below, Fig. 3a, dB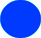 and eB). In contrast, the exchange reached around 35% when replacing nitrodopamine 1 with anachelin 5 (Fig. S20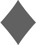 and 21†).

**Fig. 3:**
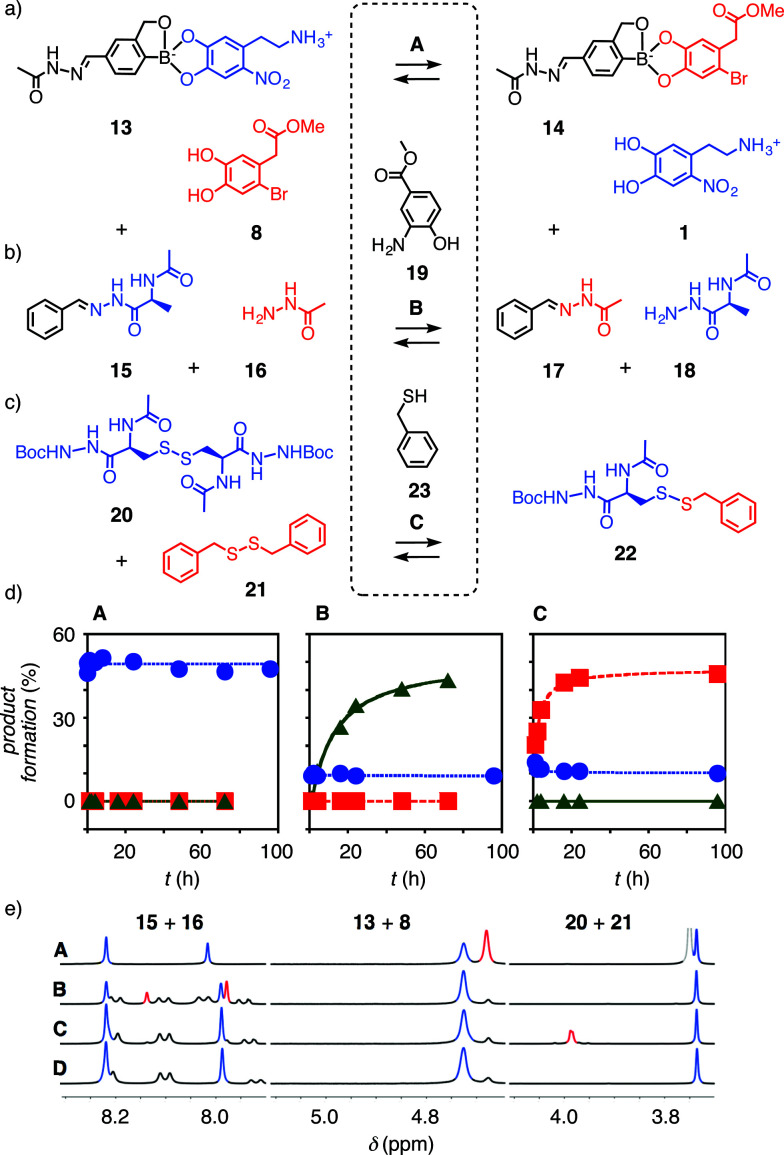
(a–c) Exchange reactions used in this study to identify orthogonality under conditions A, B and C. (d) Exchange kinetics for boronate esters (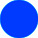, 20 mM 13, 22 mM 8) hydrazones (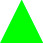, 20 mM 15, 20 mM 16) and disulfides (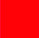, 20 mM 20, 20 mM 21) under conditions A (DMSO-*d*_6_, 10% D_2_O, 2% DIPEA), B (1.0 mM 19, 1.5 mM TFA in DMSO-*d*_6_), and C (2.0 mM 22, 2.0 mM TEA in DMSO-*d*_6_). (e) Original data showing diagnostic peaks in ^1^H NMR spectra in conditions A–C and D (DMSO-*d*_6_) of 15, 20 and 13 (blue peaks) and their exchange products (red peaks). The signals at ∼8.1 ppm disappear in conditions A due to H/D exchange.

According to the kinetics measurements using ^1^H NMR spectroscopy, the exchange reaction of boronate ester 13 with the catechol 8 in DMSO-*d*_6_ containing 10% D_2_O and 2% Hünig base, *i.e.*, conditions A, reached the equilibrium rapidly with a *t*_50_ < 3 minutes (Table 1, entry 1, Fig. 3a, dA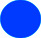, eA (red peak) and S4†). The result was a mixture of 13 and 14 at a ratio of 1 : 1.1. Reactivity in pure DMSO-*d*_6_ (condition D, Fig. 3e) and the other conditions B and C was low (Fig. 3a, dB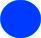, dC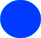, eB and C, S3–7†). Thus, standard boronate exchange conditions were already compatible with hydrazone and disulfide bonds.

**Table 1:** Orthogonal exchange kinetics^a^

	Exchange	*t*_50_ b (h)		
		A	B	C
1	Boronate	<0.05	—	—
2	Hydrazone	—	11.9	—
3	Disulfide	—	—	2.5

a See Fig. 3 for structures, conditions and original data.

b Half-life of substrates to reach equilibrium under conditions A, B and C.

It was difficult to identify the hydrazone exchange conditions compatible with the other two dynamic covalent bonds. Boronate ester 13 hydrolyzed quickly and completely under the acidic hydrazone exchange conditions even with aniline^13–15^ or with the recent anthranilic acid catalysts,^15^ because the necessary amounts of acid and catalysts were excessive (Fig. S19†). However, 2-aminophenol 19, acidified by a withdrawing ester in position 4,^16^ catalyzed hydrazone exchange with a *t*_50_ = 11.9 h to reach equilibrium at a ratio 15/17 = 1.3 : 1 under condition B (Fig. 3b, dB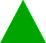, eB (red peaks), S10†; Table 1, entry 2). Most importantly, the ^1^H NMR spectrum of boronate ester 13 did not change much under condition B, and disulfides were not affected either (Fig. 3dB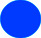, 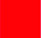, eB, S9–13†, Table 1).

To complete the series needed to demonstrate the existence of the third orthogonal dynamic covalent bond, disulfide exchange from substrates 20 and 21 to mixed product 22 and back was initiated with traces of thiol 23. Under condition C, equilibrium was reached with *t*_50_ = 2.5 h at a ratio of 20/22 = 1 : 1.6 (Fig. 3c, dC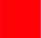, eC (red peak)), without disturbing hydrazones and boronate esters (Fig. 3dC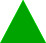, 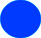, eC, S14–18†; Table 1, entry 3).

Compatibility of the third orthogonal dynamic covalent bond with functional systems was explored with multicomponent surface architecture 24 (Fig. 4). This system was obtained by the well-established SOSIP-TSE method (SOSIP = self-organizing surface-initiated polymerization; TSE = templated stack exchange, Fig. S22†).^8,9^ Namely, ring opening disulfide exchange polymerization was initiated by the thiolate groups bound on an indium-tin oxide (ITO) surface to yield, after the removal of benzaldehyde protecting group, the architecture 25. Reaction of boroxole aldehyde 10* in DMSO/AcOH with the hydrazides along the central stacks in 25 gave surface architecture 26. Incubation of electrode 26 in a solution of nitrodopamine 1 in DMSO/Hünig base afforded the desired multicomponent architecture 24.

**Fig. 4:**
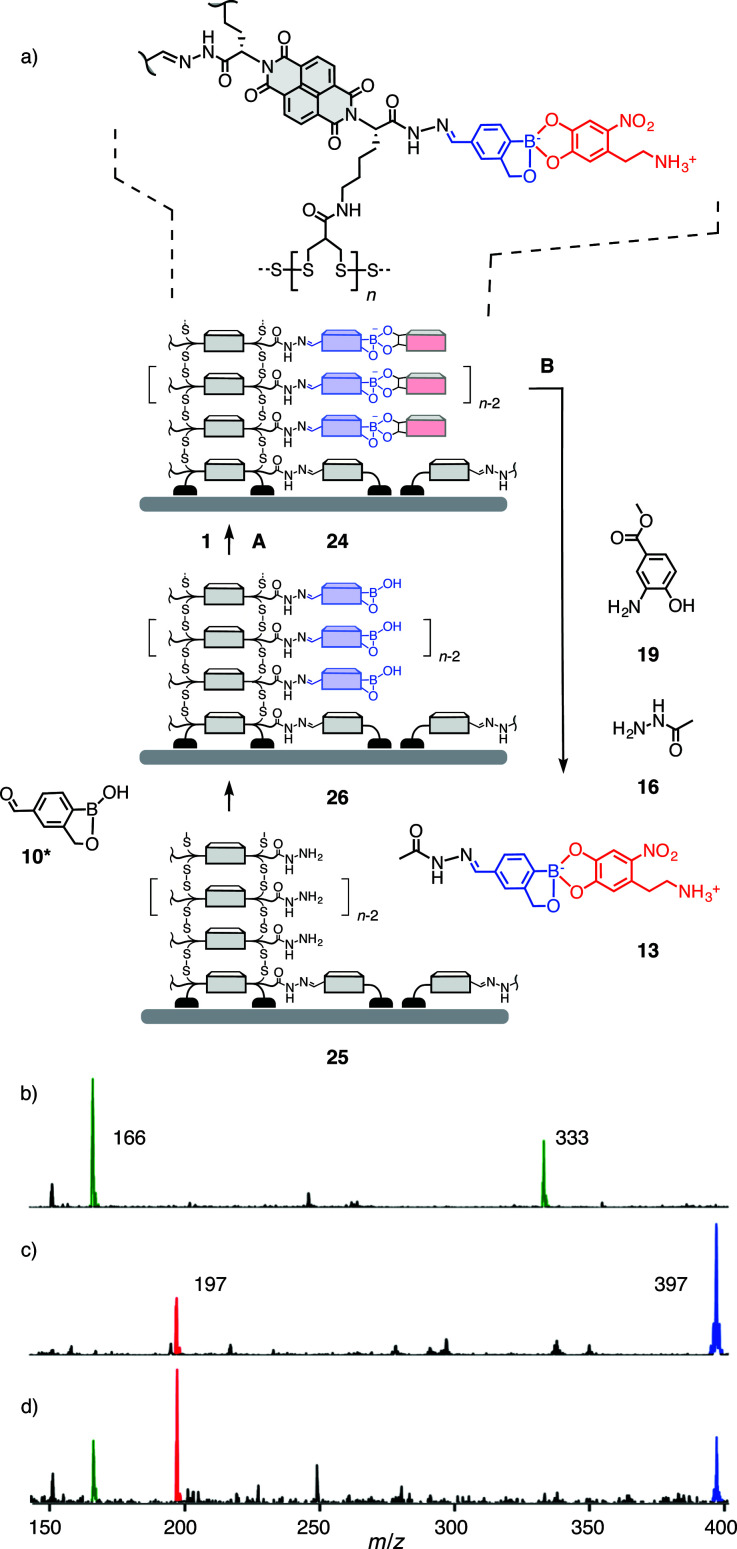
(a) Synthesis of architecture 24 and subsequent recovery of intact boronate ester 13 by orthogonal hydrazone exchange, as evidenced by ESI-MS (negative mode) of (b) catalyst 19 (*m*/*z* 166 ([M − H]^−^), 333 ([2M − H]^−^)), (c) 13 (*m*/*z* 397 ([M − H]^−^)) and catechol 1 (*m*/*z* 197 ([M − H]^−^)), and (d) the solution obtained from incubation of multicomponent surface architecture 24 with hydrazide 16 and 1.0 mM catalyst 19 in DMSO-*d*_6_, 1.5 mM TFA, for 7 h at 40 °C (10: *m*/*z* 217 ([M − H]^−^)).

While the orthogonal formation of three dynamic covalent bonds has been demonstrated for the construction of this system^9^ and others,^10^ true orthogonality in functional systems, *i.e.*, selective exchange, cleavage and formation of one bond without disturbing the others, remained to be demonstrated. The so far missing piece of evidence was hydrazone exchange in the presence of intact boronate esters. With the multicomponent surface architecture 24, this translated to the task to release intact boronate ester 13.^19^ To tackle this challenge, the conditions B elaborated above for selective hydrazone exchange were applied (20 mM hydrazide 16, 1.0 mM catalyst 19, 1.5 mM TFA in DMSO-*d*_6_). Because of the small amounts of material present per electrode, the same exchange solution was used for multiple electrodes (10 electrodes for 0.5 mL of solution). ^1^H NMR of the obtained exchange solution showed the diagnostic peak of the boronate ester 13 at 4.72 ppm and only a small peak of the hydrolysed benzoboroxole 10 at 5.01 ppm (Fig. S24†). Corroborative evidence for the release of intact ester 13 was obtained by ESI-MS (Fig. 4, S25†). These results identify the multicomponent surface architecture 24 as the first functional system that operates with three fully orthogonal organic^20^ dynamic covalent bonds, to the best of our knowledge. The insights gained here will be important for future developments in the construction of dynamic functional systems, and lead to the tantalizing question of the possible existence of a fourth orthogonal organic dynamic covalent bond.

## Supplementary Material

SC-007-C6SC01133K-s001

## References

[cit1] Wu X., Li Z., Chen X.-X., Fossey J. S., James T. D., Jiang Y.-B. (2013). Chem. Soc. Rev..

[cit2] Ramström O., Lohmann S., Bunyapaiboonsri T., Lehn J.-M. (2004). Chem.–Eur. J..

[cit3] Au-Yeung H. Y., Cougnon F. B. L., Otto S., Pantoş G. D., Sanders J. K. M. (2010). Chem. Sci..

[cit4] Li J., Nowak P., Otto S. (2013). J. Am. Chem. Soc..

[cit5] Wilson A., Gasparini G., Matile S. (2014). Chem. Soc. Rev..

[cit6] Jo H. H., Edupuganti R., You L., Dalby K. N., V Anslyn E. (2015). Chem. Sci..

[cit7] Hagihara S., Tanaka H., Matile S. (2008). J. Am. Chem. Soc..

[cit8] Sakai N., Matile S. (2011). J. Am. Chem. Soc..

[cit9] Zhang K.-D., Matile S. (2015). Angew. Chem., Int. Ed..

[cit10] Rocard L., Berezin A., De Leo F., Bonifazi D. (2015). Angew. Chem., Int. Ed..

[cit11] Gademann K. (2015). Acc. Chem. Res..

[cit12] Nguyen R., Huc I. (2003). Chem. Commun..

[cit13] Cordes E. H., Jencks W. P. (1962). J. Am. Chem. Soc..

[cit14] Wendeler M., Grinberg L., Wang X., Dawson P. E., Baca M. (2014). Bioconjugate Chem..

[cit15] Kool E. T., Park D., Crisalli P. (2013). J. Am. Chem. Soc..

[cit16] Larsen D., Pittelkow M., Karmakar S., Kool E. T. (2015). Org. Lett..

[cit17] Bérubé M., Dowlut M., Hall D. G. (2008). J. Org. Chem..

[cit18] Li M., Ishihara S., Akada M., Liao M., Sang L., Hill J. P., Krishnan V., Ma Y., Ariga K. (2011). J. Am. Chem. Soc..

[cit19] Intact poly(disulfide)s during hydrazone exchange, also under hasher conditions, is the basis of all functional studies realized so far with these systems.^5,6*k*,8,9^

[cit20] Sarma R. J., Otto S., Nitschke J. R. (2007). Chem.–Eur. J..

