# Supplementary material for: The third orthogonal dynamic covalent bond
Source: Chem Sci. 2016 Apr 20;7(7):4720–4. doi: 10.1039/c6sc01133k (PMC6014071; doi:10.1039/c6sc01133k)
Supplement: SC-007-C6SC01133K-s001 [file SC-007-C6SC01133K-s001.pdf]

Electronic Supplementary Material (ESI) for Chemical Science.
This journal is © The Royal Society of Chemistry 2016

The third orthogonal dynamic covalent bond

Inés Lascano, Kang-Da Zhang, Robin Wehlauch, Karl Gademann, Naomi Sakai,

Stefan Matile*

National Centre of Competence in Research (NCCR) Molecular Systems Engineering

Department of Organic Chemistry, University of Geneva, Geneva, Switzerland

Department of Chemistry, University of Zurich, Zurich, Switzerland

stefan.matile@unige.ch

Supporting Information

Table of Content

1. Materials and methods S2

2. Synthesis S3

3. Formation of boronate esters in solution S12

    3.1. Direct titration S12

    3.2. Reverse titration S13

4. Orthogonal dynamic covalent bonds in solution S16

    4.1. Boronate ester exchange in solution S17

    4.2. Hydrazone exchange in solution S23

    4.3. Disulfide exchange in solution S28

    4.4. Control experiments in solution S33

5. Orthogonal dynamic covalent bonds on surfaces S36

6. Supporting references S40

7. NMR spectra S41

1. Materials and methods

As in reference S1. Briefly, reagents for synthesis were purchased from Fluka, Sigma-Aldrich, Apollo Scientific, Alfa Aesar, Fluorochem and Acros, buffers and salts of the best grade available from Fluka or Sigma-Aldrich and used as received. Unless stated otherwise, column chromatography was carried out on silica gel 60 (Silicycle, 40-63 µm). Analytical TLC was performed on silica gel 60 (Fluka, 0.2 mm or Merck, 0.25 mm). Preparative, reversed-phase HPLC was performed with a Varian 218 pump, a Dionex VWD-3400 detector and a Dionex AFC-3000 fraction collector on a Phenomenex Gemini-NX 10µ C18 110A (250 x 21.2 mm) column eluting with a constant gradient of MeCN in H_2O from 5–100% over 30 min at a flow rate of 20 mL min^{-1}. Analytical, reversed-phase UPLC was performed with an Agilent 1290 Infinity LC system on a Zorbax SB-C18 (1.8 µm, 50 x 2.1 mm) column eluting with a linear gradient of MeCN in H_2O containing 0.1% formic acid at a flow rate of 0.8 mL min^{-1}. UV-Vis spectra were recorded on a JASCO V-650 spectrophotometer equipped with a stirrer and a temperature controller and are reported as maximal absorption wavelength λ in nm. Melting points (Mp) were recorded on a Büchi Melting Point M-565 or B-545. $[\alpha]^{20}_{D}$ values were recorded on a Jasco P-1030 Polarimeter. IR spectra were recorded on a Perkin Elmer Spectrum One FT-IR spectrometer (ATR, Golden Gate) or a Varian 800 FT-IR (ATR) spectrometer and are reported as wavenumbers ν in cm^{-1} with band intensities indicated as s (strong), m (medium), w (weak) or br (broad). ^{1}H and ^{13}C NMR spectra were recorded (as indicated) either on a Bruker 300 MHz or 400 MHz spectrometer and are reported as chemical shifts (δ) in ppm relative to (residual) solvent signal. Spin multiplicities are reported as a singlet (s), doublet (d), triplet (t), quartet (q), doublet of doublets (dd), doublet of quartets (dq) with coupling constants (J) given in Hz, or multiplet (m). ^{13}C resonances were assigned with the aid of additional information from DEPT 135 spectra. ESI-MS for the characterization of new compounds was performed on an ESI API 150EX and are reported as mass-per-charge ratio m/z (intensity in %, [assignment]). HR ESI-MS for the characterization of new

compounds were performed on a QSTAR Pulsar (AB/MDS Sciex) or a Bruker maXis 4G QTOF and are reported as mass per charge ratio *m/z* calculated and observed.

Abbreviations. $AcNHNH_2$: Acethydrazide; Boc: *tert*-Butyloxycarbonyl; Calcd: Calculated; DIPEA: *N*,*N*-Diisopropylethylamine; DMF: *N*,*N*-Dimethylformamide; DMSO: Dimethylsulfoxide; EDCI: 1-Ethyl-3-(3-dimethylaminopropyl)carbodiimide**;** HOBt: Hydroxybenzotriazole; ITO: Indium tin oxide; NMP: *N*-methyl-2-pyrrolidone; PE: Petroleum ether; PTLC: Preparative thin layer chromatography; rt: Room temperature; TEA: Triethylamine; TES: Tetraethylsilane; TFA: Trifluoroacetic acid; TMS-CF_3: Trifluoromethyltrimethylsilane.

2. Synthesis

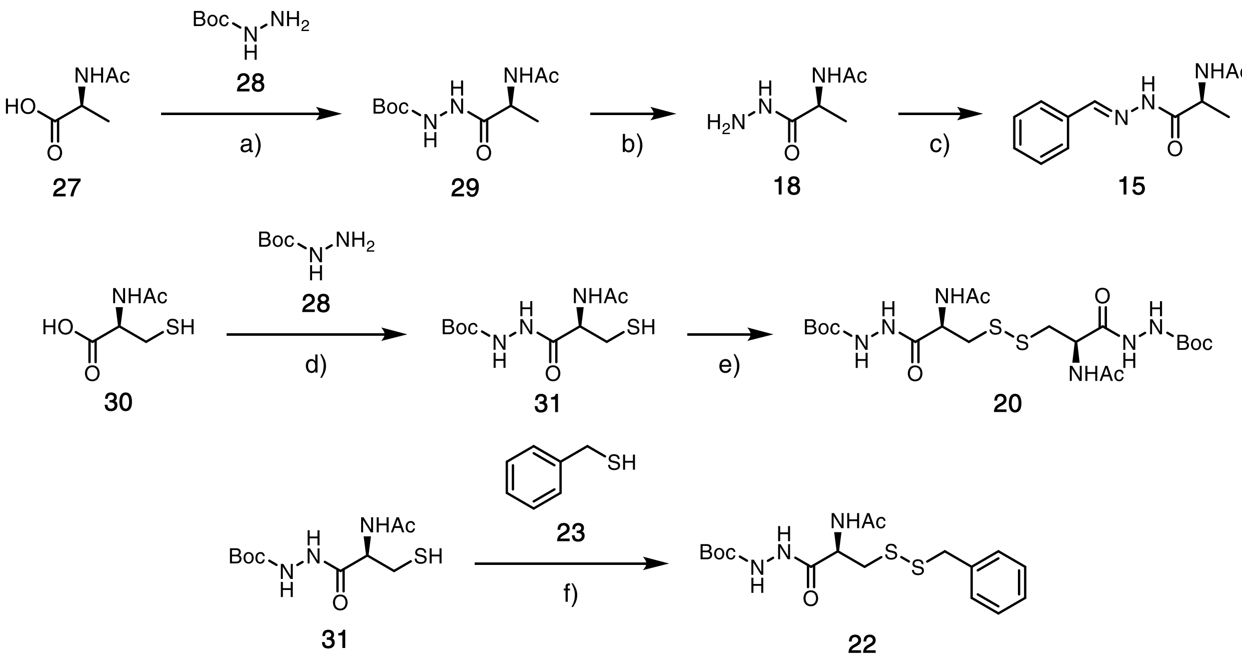

Scheme S1. a) HOBt, EDCI, CH_2Cl_2, DMF, rt, 20 h, 66%; b) TFA, CH_2Cl_2, rt, 1 h, 95%; c) benzaldehyde, thioanisole, CH_2Cl_2, rt, 1 h, quantitative; d) HOBt, EDCI, CH_2Cl_2, DMF, rt, 20 h, 70%; e) H_2O_2, NaI, EtOAc, rt, 3 h, 83%; f) H_2O_2, NaI, EtOAc, rt, 1 h, 60%.

Compound 29. To a solution of *N*-acetyl-L-alanine **27** (195 mg, 1.49 mmol) and **28** (340 mg, 2.57 mmol) in a mixture of distilled DMF (1.5 mL) and CH_2Cl_2 (12.5 mL) were added HOBt·H_2O (370 mg, 2.08 mmol) and EDCI (324 mg, 1.69 mmol) and the mixture was left under stirring at rt

for 20 h. The solvents were evaporated *in vacuo* and the crude subjected to flash column chromatography (SiO_2, CH_2Cl_2/MeOH/acetone 42:6:2). Further purification by flash column chromatography (SiO_2, EtOAc/MeOH 9:1) afforded compound **29** as a colorless solid (239 mg, 66%). R_f (CH_2Cl_2/MeOH 9:1): 0.41; Mp: 173-174 °C; $[\alpha]^{20}_D$: -70 (*c* 0.84, CH_2Cl_2/MeOH 4:1); IR (neat): 3265 (w), 2980 (w), 1651 (m), 1531 (m), 1368 (m), 1241 (m), 1158 (s), 1013 (w); ^1H NMR (400 MHz, DMSO-d_6): 9.61 (s, 1H), 8.72 (s, 1H), 8.05 (d, ^3J (H,H) = 7.9 Hz, 1H), 4.31 (dq, ^3J (H,H) = 7.9 Hz, ^3J (H,H) = 7.1 Hz, 1H), 1.82 (s, 3H), 1.38 (s, 9H), 1.19 (d, ^3J (H,H) = 7.1 Hz, 3H); ^{13}C NMR (101 MHz, DMSO-d_6): 172.1 (C), 168.9 (C), 155.1 (C), 79.1 (C), 46.4 (CH), 28.1 (CH_3), 22.5 (CH_3), 18.5 (CH_3); ESI-MS ($CHCl_3$/MeOH 1:1, HCO_2H 0.1%): 999 (12, $[4M+NH_4]^+$), 754 (44, $[3M+NH_4]^+$), 509 (100, $[2M+NH_4]^+$), 492 (40, $[2M+H]^+$), 264 (69, $[M+NH_4]^+$), 247 (46, $[M+H]^+$), 114 (67, $[M\text{-NHNHBoc}+H]^+$); HRMS (ESI, $CHCl_3$/MeOH 1:1, HCO_2H 0.1%) calcd for $C_{10}H_{19}N_3O_4$+Na: 268.1268, found: 268.1267.

Compound 18. To a solution of **29** (17 mg, 68 µmol) in CH_2Cl_2 (5 mL) was added TFA (1.0 mL), and the mixture was left under stirring at rt for 1 h. The solvents were then evaporated *in vacuo* and the residue dissolved in Et_2O. Evaporation *in vacuo* of the solvent afforded compound **18** as a colorless oil (9 mg, 95%). R_f (CH_2Cl_2/MeOH 9:1): 0.13; $[\alpha]^{20}_D$: -31 (*c* 0.59, MeOH); IR (neat): 3262 (w) 1645 (s), 1535 (s), 1451 (w), 1432 (w), 1376 (m), 1294 (w), 1200 (s), 1179 (s), 1133 (s), 1049 (w), 1026 (m), 999 (m), 833 (w), 800 (m); ^1H NMR (400 MHz, DMSO-d_6): 10.73 (br, 1H), 8.26 (d, ^3J (H,H) = 7.0 Hz, 1H), 4.27 (dq, ^3J (H,H) = 7.0 Hz, ^3J (H,H) = 7.0 Hz, 1H), 1.84 (s, 3H), 1.23 (d, ^3J (H,H) = 7.0 Hz, 3H); ^{13}C NMR (101 MHz, DMSO-d_6): 172.1 (C), 169.3 (C), 46.9 (CH), 22.3 (CH_3), 17.6 (CH_3); ESI-MS ($CHCl_3$/MeOH 1:1, HCO_2H 0.1%): 314 (35, $[2M+Na]^+$), 292 (63, $[2M+H]^+$), 169 (37, $[M+Na]^+$), 146 (100, $[M+H]^+$), 114 (52, $[M\text{-}NH_2NH+H]^+$); HRMS (ESI, $CHCl_3$/MeOH 1:1, HCO_2H 0.1%) calcd for $C_5H_{11}N_3O_2$: 168.0744, found: 168.0744.

Compound 15. To a solution of **29** (156 mg, 0.64 mmol) in a mixture of CH_2Cl_2 (10 mL) and thioanisole (1.2 mL) was added TFA (5.0 mL) and the mixture was left under stirring at rt for 1 h.

After addition of benzaldehyde (5.0 mL, 92 mmol), the mixture was left under stirring at rt for 1 h. The mixture was subjected to liquid-liquid extraction with sat. aq. $NaHCO_3$ and EtOAc. The organic phase was dried over Na_2SO_4, filtered and concentrated. Purification of the remaining oil by flash column chromatography (SiO_2, CH_2Cl_2/MeOH 9:1) afforded compound **15** as a colorless solid (151 mg, quantitative). R_f (CH_2Cl_2/MeOH 9:1): 0.50; $[\alpha]^{20}_D$: +29 (*c* 0.96, MeOH); Mp: decomp. > 170 °C; IR (neat): 3274 (w), 3210 (w), 3067 (w), 1667 (s), 1633 (s), 1549 (s), 1444 (w), 1371 (m), 1323 (w), 1281 (w), 1230 (m), 1209 (w), 1146 (m), 1107 (w), 1058 (w), 949 (m), 754 (s), 693 (s), 604 (s), 562 (w); ^1H NMR (400 MHz, CD_3OD, *n/n*: stereoisomeric peaks): 8.15 / 7.95 (s, 1H), 7.78 - 7.72 / 7.70 - 7.65 (m, 2H), 7.44 - 7.35 (m, 3H), 5.32 / 4.43 (q, ^3J (H,H) = 7.2 Hz, 1H), 2.00 (s, 3H), 1.43 / 1.42 (d, ^3J (H,H) = 7.2 Hz, 3H); ^{13}C NMR (101 MHz, CD_3OD, *n/n*: stereoisomeric peaks): 176.1 / 172.0 (C), 173.2 / 172.9 (C), 150.3 / 146.3 (CH), 135.5 / 135.3 (C), 131.6 / 131.2 (CH), 129.81 / 129.77 (CH), 128.7 / 128.2 (CH), 49.5 / 47.5 (CH), 22.43 / 22.38 (CH_3), 18.0 / 17.6 (CH_3); ESI-MS ($CHCl_3$/MeOH 1:1, HCO_2H 0.1%): 490 (22, $[2M+Na]^+$), 468 (50, $[2M+H]^+$), 256 (33, $[M+Na]^+$), 234 (100, $[M+H]^+$); HRMS (ESI, $CHCl_3$/MeOH 1:1, HCO_2H 0.1%) calcd for $C_{12}H_{15}N_3O_2$: 234.1237, found: 234.1239.

Compound 31. To a solution of *N*-acetyl-L-cysteine **30** (1.00 g, 6.13 mmol) and **28** (1.34 g, 10.1 mmol) in a mixture of distilled DMF (6 mL) and CH_2Cl_2 (50 mL) were added HOBt·H_2O (1.46 g, 8.22 mmol) and EDCI (1.30 g, 6.79 mmol) and the mixture was left under stirring at rt for 20 h. The solvents were evaporated *in vacuo* and the crude subjected to flash column chromatography (SiO_2, CH_2Cl_2/MeOH 9:1). Further purification by flash column chromatography (SiO_2, EtOAc) afforded compound **31** as a colorless oil (1.20 g, 70%). R_f (CH_2Cl_2/MeOH 9:1): 0.47; $[\alpha]^{20}_D$: -45 (*c* 0.92, MeOH); IR (neat): 3271 (m), 2978 (w), 1656 (s), 1530 (m), 1369 (m), 1243 (m), 1161 (s), 1045 (w), 1014 (w); ^1H NMR (400 MHz, $CDCl_3$): 8.91 (s, 1H), 7.02 - 6.86 (m, 2H), 4.70 – 4.76 (m, 1H), 3.01 - 2.70 (m, 2H), 2.05 (s, 3H), 1.83 – 1.89 (m, 1H), 1.45 (s, 9H); ^{13}C NMR (101 MHz, $CDCl_3$): 171.1 (C), 170.1 (C), 155.4 (C), 82.1 (C), 53.2 (CH), 28.3 (CH_3), 26.8

(CH_2), 23.2 (CH_3); ESI-MS ($CHCl_3$/MeOH 1:1, HCO_2H 0.1%): 1127 (5, $[4M+NH_4]^+$), 850 (28, $[3M+NH_4]^+$), 573 (85, $[2M+NH_4]^+$), 556 (20, $[2M+H]^+$), 296 (100, $[M+NH_4]^+$), 279 (36, $[M+H]^+$), 147 (90, $[M\text{-}NHNHBoc+H]^+$); HRMS (ESI, $CHCl_3$/MeOH 1:1, HCO_2H 0.1%) calcd for $C_{10}H_{19}N_3O_4S$+Na: 300.0989, found: 300.0986.

Compound 20. To a solution of **31** (598 mg, 2.16 mmol) in EtOAc (30 mL) were added NaI (10 mg, 70 μmol) and H_2O_2 (30% solution, 0.22 mL, 2.2 mmol) and the mixture was left under stirring at rt for 3 h, after which more H_2O_2 (30% solution, 0.22 mL, 2.2 mmol) was added. After 5 minutes stirring at rt, the solution was subjected to liquid-liquid extraction with sat. aq. $Na_2S_2O_3$, EtOAc, and then CH_2Cl_2/MeOH 9:1. The organic phases were dried over Na_2SO_4, filtered and concentrated. The resulting solid was subjected to solid-liquid extraction with Et_2O, affording compound **20** as a colorless solid (496 mg, 83%). R_f (CH_2Cl_2/MeOH 9:1): 0.41; Mp: decomp. > 158 °C; $[\alpha]^{20}_D$: -101 (*c* 0.98, MeOH); IR (neat): 3280 (w), 1715 (m), 1661 (s), 1555 (m), 1368 (s), 1297 (w), 1258 (w), 1161 (m), 1048 (w), 1022 (w), 773 (w), 713 (m), 620 (m), 588 (s); ^{1}H NMR (400 MHz, CD_3OD): 4.79 (dd, ^3J (H,H) = 9.3 Hz, ^3J (H,H) = 5.1 Hz, 2H), 3.27 - 2.87 (m, 4H), 2.00 (s, 6H), 1.47 (s, 18H); ^{13}C NMR (101 MHz, CD_3OD): 173.4 (C), 172.3 (C), 157.5 (C), 82.0 (C), 52.3 (CH), 41.4 (CH_2), 28.5 (CH_3), 22.5 (CH_3); ESI-MS ($CHCl_3$/MeOH 1:1, HCO_2H 0.1%): 1123 (28, $[2M+NH_4]^+$), 1106 (18, $[2M+H]^+$), 576 (41, $[M+Na]^+$), 571 (97, $[M+NH_4]^+$), 553 (100, $[M+H]^+$); HRMS (ESI, $CHCl_3$/MeOH 1:1, HCO_2H 0.1%) calcd for $C_{20}H_{36}N_6O_8S_2$: 553.2109, found: 553.2111.

Compound 22. To a solution of **31** (6 mg, 20 μmol) and benzylthiol **23** (25.0 μL, 216 μmol) in EtOAc (2 mL) were added NaI (3 mg, 20 μmol) and H_2O_2 (30% solution, 50 μL, 490 mmol) and the mixture was left under stirring at rt for 1 h. The solution was then subjected to liquid-liquid extraction with sat. aq. $Na_2S_2O_3$ and CH_2Cl_2. The organic phases were dried over Na_2SO_4, filtered and concentrated. The resulting solid was purified by PTLC (CH_2Cl_2/MeOH/acetone 44:3:3), affording compound **22** as a colorless oil (5 mg, 60%). R_f (CH_2Cl_2/MeOH 9:1): 0.31; $[\alpha]^{20}_D$: -25 (*c*

0.18, CH_2Cl_2); IR (neat): 3283 (m), 1655 (s), 1530 (m), 1493 (m), 1371 (s), 1244 (s), 1159 (s), 911 (w), 766 (w), 728 (s), 699 (s); ^{1}H NMR (400 MHz, $CDCl_3$): 8.10 (s, 1H), 7.37 – 7.27 (m, 5H), 6.41 (s, 1H), 6.22 (d, ^3J (H,H) = 7.1 Hz, 1H), 4.65 (q, ^3J (H,H) = 7.1 Hz, 1H), 3.90 – 3.97 (m, 2H), 2.75 (d, ^3J (H,H) = 7.1 Hz, 2H), 2.03 (s, 3H), 1.46 (s, 9H); ^{13}C NMR (101 MHz, $CDCl_3$): 170.9 (C), 169.9 (C), 154.9 (C), 137.0 (C), 129.6 (CH), 128.8 (CH), 127.8 (CH), 82.1 (C), 51.0 (CH), 43.4 (CH_2), 38.7 (CH_2), 28.3 (CH_3), 23.3 (CH_3); ESI-MS ($CHCl_3$/MeOH 1:1, HCO_2H 0.1%): 417 (51, $[M+NH_4]^+$), 400 (83, $[M+H]^+$), 267 (100, $[M\text{-}NHNHBoc+H]^+$); HRMS (ESI, $CHCl_3$/MeOH 1:1, HCO_2H 0.1%) calcd for $C_{17}H_{25}N_3O_4S_2$: 400.1359, found: 400.1361.

Compounds 10 and 10* were synthesized according to the procedure reported in ref. S1.

Compound 19 was synthesized according to the procedure reported in ref. S2.

Compound 17 was synthesized according to the procedure reported in ref. S3.

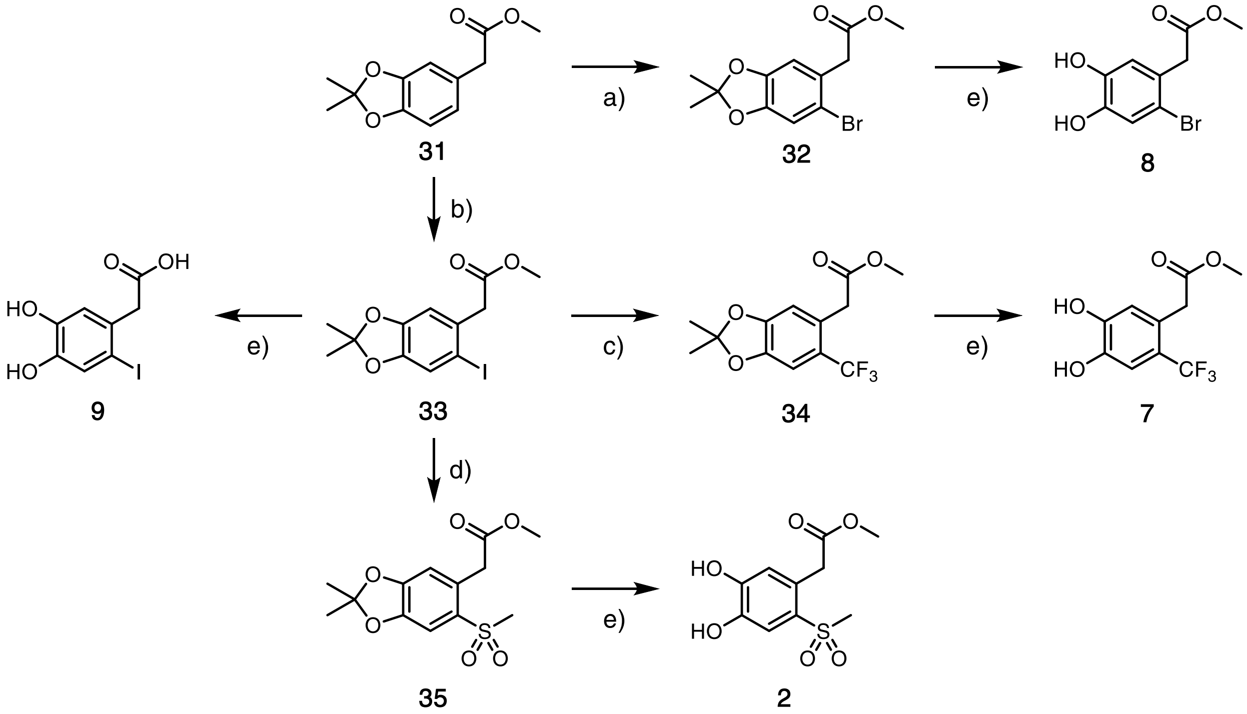

Scheme S2. a) KBr, oxone, acetone, H_2O, rt, 1 h, quantitative; b) I_2, Ag_2SO_4, MeOH, rt, 1 h, 99%; c) TMS-CF_3, KF, CuI, NMP, 80 °C (sealed), 22 h, 88%; d) CH_3SO_2Na, L-proline, $Cu(OAc)_2$, DMSO, 100 °C (sealed), 24 h, 77%; e) TFA, H_2O, 0 °C to rt, 2.5 h, 45–78%.

Compound 32. To a solution of **31** (300 mg, 1.35 mmol) in acetone (14 mL) and water (8.0 mL) was added oxone (873 mg, 1.42 mmol) and KBr (193 mg, 1.62 mmol) and the mixture was stirred at rt for 1 h. The mixture was filtered and acetone was removed under reduced pressure. Sat. aq. $Na_2S_2O_3$ was added and the mixture extracted with CH_2Cl_2. The combined organic layers were dried over Na_2SO_4 and evaporated to give compound **32** (409 mg, quant.) as a yellow oil. R_f (pentane/Et_2O, 10:1): 0.43; Mp: 49-50 °C; IR (neat): 2991 (w), 2952 (w), 1738 (s), 1489 (s), 1380 (m), 1215 (s), 1149 (s), 980 (s), 855 (s), 783 (m); ^1H NMR (400 MHz, $CDCl_3$): 6.92 (s, 1H), 6.67 (s, 1H), 3.72 (s, 3H), 3.68 (s, 2H), 1.66 (s, 6H); ^{13}C NMR (101 MHz, $CDCl_3$): 171.4, 147.7, 147.4, 126.3, 119.5, 114.8, 112.6, 110.9, 52.3, 41.4, 26.0; HRMS (ESI, MeOH) calcd for $C_{12}H_{13}BrO_4$+Na: 322.9889, found: 322.9888.

Compound 8. Compound **32** (161 mg, 535 µmol) was cooled to 0 °C and precooled 20% aq. TFA (1 mL) was added. After 5 minutes, the ice bath was removed and stirring continued at room

temperature for 2.5 h. Volatiles were removed under reduced pressure and the residue was purified by preparative reversed-phase HPLC to yield **8** (102 mg, 73%) as an off-white solid. Mp: 106-108 °C; IR (neat): 3370 (br, m), 2955 (w), 2364 (w), 1712 (s), 1510 (s), 1436 (m), 1339 (m), 1282 (s), 1221 (s), 1162 (s), 1011 (m), 870 (m), 821 (m); ^{1}H NMR (400 MHz, CD_3OD): 6.94 (s, 1H), 6.75 (s, 1H), 3.68 (s, 3H), 3.63 (s, 2H); ^{13}C NMR (101 MHz, CD_3OD): 173.5, 146.8, 146.1, 126.2, 119.9, 119.1, 114.0, 52.4, 41.4; HRMS (ESI, MeOH): calcd for $C_9H_9BrO_4$+Na: 282.9576, found: 282.9576.

Compound 33. To a solution of **31** (302 mg, 1.36 mmol) in MeOH (8.0 mL) was added Ag_2SO_4 (636 mg, 2.04 mmol) and iodine (517 mg, 2.04 mmol) and the brown mixture was stirred at rt for 1 h during which the brown color faded. The mixture was filtered, diluted with water and extracted with Et_2O. The combined organic layers were dried over Na_2SO_4 and evaporated. The residue was eluted through a short silica column (pentane/Et_2O, 5:1, 1% Et_3N) to yield compound **33** (468 mg, 99%) as an orange oil that solidified overnight at 4 °C. R_f (pentane/Et_2O, 5:1): 0.55; Mp: 68-69 °C; IR (neat): 2988 (w), 2956 (w), 1728 (s), 1489 (s), 1458 (m), 1380 (m), 1335 (m), 1224 (s), 1189 (s), 1151 (s), 981 (s), 836 (s); ^{1}H NMR (400 MHz, $CDCl_3$): 7.15 (s, 1H), 6.71 (s, 1H), 3.72 (s, 4H), 3.70 (s, 2H), 1.66 (s, 7H); ^{13}C NMR (101 MHz, $CDCl_3$): 171.4, 148.4, 147.7, 130.1, 119.4, 118.5, 110.4, 88.2, 52.3, 46.0, 26.0; HRMS (ESI, MeOH): calcd for $C_{12}H_{13}IO_4$+Na: 370.9751, found: 370.9750.

Compound 9. Deprotection of **33** (120 mg, 345 µmol) was performed analogously to **8** yielding **9** (66 mg, 62%) as an off-white solid. Mp: 112-114 °C; IR (neat): 3385 (br, m), 2952 (w), 2360 (w), 2338 (w), 1711 (s), 1504 (s), 1437 (m), 1335 (m), 1279 (s), 1224 (s), 1162 (s); ^{1}H NMR (400 MHz, CD_3OD): 7.18 (s, 1H), 6.76 (s, 1H), 3.68 (s, 3H), 3.64 (s, 2H); ^{13}C NMR (101 MHz, CD_3OD): 173.5, 147.1, 146.7, 130.1, 126.3, 118.6, 87.5, 52.5, 45.9; HRMS (ESI, MeOH): calcd for $C_9H_9IO_4$+Na: 330.9438, found: 330.9438.

Compound 34. A mixture of **33** (468 mg, 1.34 mmol), KF (156 mg, 2.69 mmol; predried at 100 °C under high vacuum) and CuI (538 mg, 2.82 mmol) was suspended in NMP (3.7 mL). TMS-CF_3 (0.40 mL, 2.69 mmol) was added and the mixture was heated to 80 °C for 22 h in a sealed tube. The mixture was poured into dilute aq. NaCl and extracted with CH_2Cl_2. The combined organic layers were subjected to liquid-liquid extraction with water, dried over Na_2SO_4 and the solvent evaporated. The residue was subjected to flash column chromatography (pentane/Et_2O, 5:1, 1% Et_3N) to yield **34** (341 mg, 88%) as a yellow oil. R_f (pentane/Et_2O, 5:1): 0.66; IR (neat): 2995 (w), 2956 (w), 1743 (m), 1504 (m), 1386 (m), 1295 (m), 1218 (m), 1144 (s), 1109 (s), 979 (m), 870 (m); ^{1}H NMR (400 MHz, $CDCl_3$): 6.99 (s, 1H), 6.72 (s, 1H), 3.71 (s, 5H), 1.69 (s, 6H); ^{13}C NMR (101 MHz, $CDCl_3$): 171.4, 149.9, 146.6, 126.6 (q, ^3J (C,F) = 2.1 Hz), 124.2 (q, ^1J (C,F) = 273.2 Hz), 121.8 (q, ^2J (C,F) = 30.7 Hz), 119.8, 112.1, 106.2 (q, ^3J (C,F) = 6.0 Hz), 52.2, 37.7 (q, ^4J (C,F) = 1.8 Hz), 25.9; HRMS (ESI, MeOH): calcd for $C_{13}H_{13}F_3O_4$+Na: 313.0658, found: 313.0658.

Compound 7. Deprotection of **34** (200 mg, 689 µmol) was performed analogously to **8** yielding **7** (134 mg, 78%) as an off-white solid. Mp: 131-133 °C; IR (neat): 3507 (m), 3293 (br, m), 1712 (s), 1459 (m), 1344 (m), 1310 (s), 1281 (m), 1234 (s), 1159 (m), 1114 (s), 1018 (m), 841 (m); ^{1}H NMR (400 MHz, CD_3OD): 7.03 (s, 1H), 6.80 (s, 1H), 3.67 (s, 3H), 3.66 (s, 2H); ^{13}C NMR (101 MHz, CD_3OD): 173.6, 149.5, 145.4, 126.1 (q, ^1J (C,F)= 271.5 Hz), 125.4 (q, ^3J (C,F) = 1.8 Hz), 120.9 (q, ^2J (C,F) = 30.2 Hz), 120.6, 114.1 (q, ^3J (C,F) = 5.5 Hz), 52.5, 38.1 (q, ^4J (C,F) = 2.2 Hz); HRMS (ESI, MeOH): calcd for $C_{10}H_9F_3O_4$+Na: 273.0345, found: 273.0346.

Compound 35. A mixture of **33** (153 mg, 439 µmol), sodium methanesulfinate (67.3 mg, 659 µmol), L-proline (20.4 mg, 176 µmol) and $Cu(OAc)_2$ (31.9 mg, 176 µmol) was suspended in DMSO and the mixture was heated to 100 °C for 24 h in a sealed tube. The reaction mixture was cooled to rt and quenched with sat. aq. NH_4Cl, diluted with water and extracted with EtOAc. The combined organic layers were subjected to a liquid-liquid extraction with water and brine, and dried over Na_2SO_4. The residue was subjected to flash column chromatography (pentane/Et_2O, 1:1, 1%

Et_3N) to yield **35** (102 mg, 77%) as a yellowish solid. R_f (pentane/Et_2O, 1:1): 0.41; Mp: 117-119 °C; IR (neat): 3019 (w), 2988 (w), 2966 (w), 2923 (w), 1727 (s), 1496 (s), 1300 (m), 1244 (m), 1196 (m), 1136 (m), 983 (m), 830 (s), 764 (m); ^{1}H NMR (400 MHz, $CDCl_3$): 7.38 (s, 1H), 6.68 (s, 1H), 4.11 (s, 2H), 3.73 (s, 3H), 3.10 (s, 3H), 1.70 (s, 6H); ^{13}C NMR (101 MHz, $CDCl_3$): 171.9, 151.8, 147.4, 131.7, 129.0, 120.8, 112.3, 109.7, 52.4, 45.1, 38.2, 26.1; HRMS (ESI, MeOH): calcd for $C_{13}H_{16}O_6S$+Na: 323.0560, found: 323.0561.

Compound 2. Deprotection of **35** (96.0 mg, 320 µmol) was performed analogously to **8** yielding **2** (37.3 mg, 45%) as a hygroscopic, orange solid. IR (neat): 3362 (br, m), 3027 (w), 2957 (w), 1721 (m), 1595 (m), 1520 (m), 1440 (m), 1293 (s), 1208 (m), 1128 (s), 764 (m); ^{1}H NMR (400 MHz, CD_3OD): 7.38 (s, 1H), 6.78 (s, 1H), 3.99 (s, 2H), 3.69 (s, 3H), 3.06 (s, 3H); ^{13}C NMR (101 MHz, CD_3OD): 173.8, 151.6, 146.0, 130.3, 127.5, 120.7, 117.6, 52.5, 45.2, 38.6; HRMS (ESI, H_2O): calcd for $C_{10}H_{12}O_6S$+Na: 283.0247, found: 283.0249.

Compound 1 was prepared according to the procedure reported in ref. S4.

Compound 5 was prepared according to the procedure reported in ref. S5.

Compound 31 was prepared according to the procedure reported in ref. S6.

3. Formation of boronate esters in solution

3.1. Direct titration

The dissociation constants (K_D) of the boronate esters in aqueous 75% MeOH (100 mM HEPES, pH 7.8) were determined with UV-Vis titration experiments (Fig. 2). Representative examples are shown in Fig. S1. The K_D's were deduced from equation (S1) for 1:1 binding motif:[S7]

$$\Delta A=((\Delta A_{max})/[\mathbf{C}]_0) \times (0.5 \times [\mathbf{B}] + 0.5 \times ([\mathbf{C}]_0 + K_D) - (0.5 \times (([\mathbf{B}]^2) + (2 \times ([\mathbf{B}]) \times (K_D - [\mathbf{C}]_0)) + (K_D + [\mathbf{C}]_0)^2)^{0.5}) \quad \text{(S1)}$$

where $\Delta A = A - A_0$, [**B**] = concentration of **10**, **11** or **12**, and [**C**] = concentration of the catechol. Values for couples **10-3**, **10-4**, **10-6**, **12-3**, **12-4** and **12-6** were taken from ref S1.

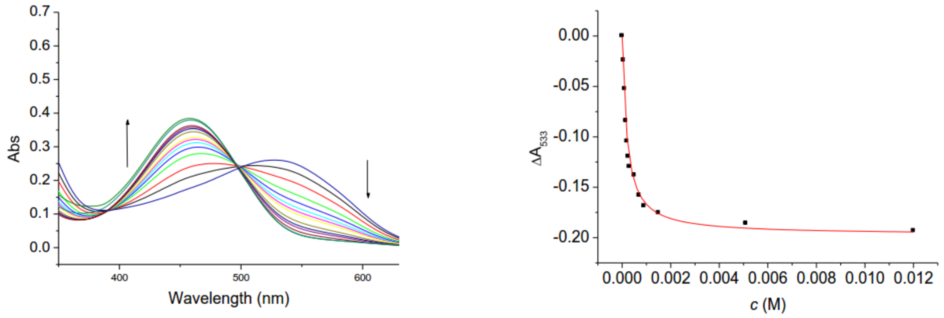

Fig. S1. UV-Vis spectra of **4** (45 µM) in aqueous 75% MeOH (100 mM HEPES, pH 7.8) in the presence of 0-12 mM **11**.

3.2. Reverse titration

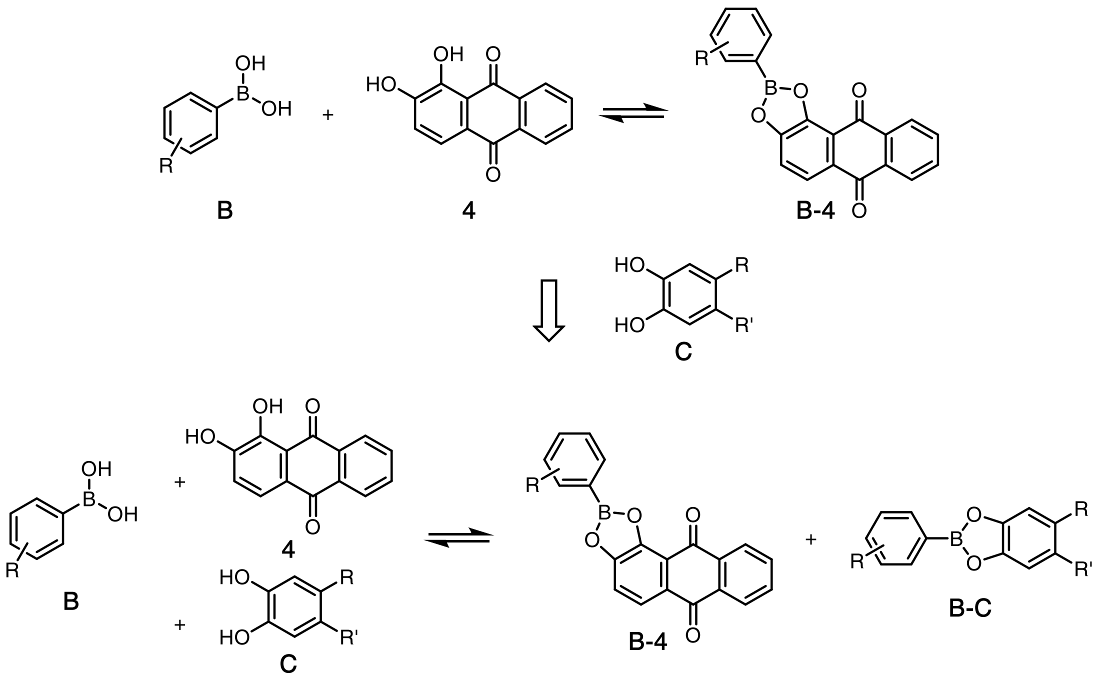

Scheme S3. Equilibria in solution during the determination of the dissociation constant of boronate ester **B-C** by reverse titration of boronate esters **B-4** with catechols **C**.

The K_D's of non-UV-active boronate esters (**B-C**) were determined similarly by reverse titration, in which alizarin red (**4**) was added to the mixture to serve as an indicator (Scheme S3). Representative examples are shown in Fig. S2. The K_D(**B-C**) can be expressed by the following equation:

$$K_D(\mathbf{B\text{-}C}) = ([\mathbf{B_f}] \text{ x } [\mathbf{C_f}]) / [\mathbf{B\text{-}C}] \quad \text{(S2)}$$

where [$\mathbf{B_f}$] = concentration of free boronic acid **B** (**10-12**), [$\mathbf{C_f}$] = concentration of free catechol **C** (**2**, **5**, **7-9**), [**B-C**] = concentration of boronate ester **B-C**.

Considering

$$[\mathbf{B}] = [\mathbf{B_f}] + [\mathbf{B\text{-}4}] + [\mathbf{B\text{-}C}] \quad \text{(S3)}$$

where [**B-4**] = concentration of boronate ester **B-4**, we can obtain

$$[\mathbf{B\text{-}C}] = [\mathbf{B}] - ([\mathbf{B_f}] + [\mathbf{B\text{-}4}]) \quad \text{(S4)}$$

The value of ([$\mathbf{B_f}$] + [**B-4**]) can be obtained by comparing the absorbance at 533 nm of the reverse titration (e.g., fig. S2) with the direct titration of **4** with **B** (e.g., fig. S1). By finding the same absorbance at 533 nm in the latter, we can find

$$[\mathbf{B_f}] + [\mathbf{B\text{-}4}] = [\mathbf{B}]_{direct} \tag{S5}$$

where $[\mathbf{B}]_{direct}$ = concentration of boronic acid **B** retrieved from the direct titration, and thus

$$[\mathbf{B_f}] = [\mathbf{B}]_{direct} - [\mathbf{B\text{-}4}] \tag{S6}$$

Combining eq. S4 with eq. S5, we obtain

$$[\mathbf{B\text{-}C}] = [\mathbf{B}] - [\mathbf{B}]_{direct} \tag{S7}$$

Additionally, we can rewrite

$$[\mathbf{C}]_0 = [\mathbf{C_f}] + [\mathbf{B\text{-}C}] \tag{S8}$$

as

$$[\mathbf{C_f}] = [\mathbf{C}]_0 - [\mathbf{B\text{-}C}] \tag{S9}$$

As a result, eq. S2 can be rewritten as

$$K_D(\mathbf{B\text{-}C}) = (([\mathbf{B}]_{direct} - [\mathbf{B\text{-}4}]) \text{ x } ([\mathbf{C}]_0 - ([\mathbf{B}] - [\mathbf{B}]_{direct})) / ([\mathbf{B}] - [\mathbf{B}]_{direct}) \tag{S10}$$

where, [**B-4**] can be obtained by comparing the absorbance at 533 nm to those obtained by the direct titration of **4** with **B** (e.g., fig. S1).

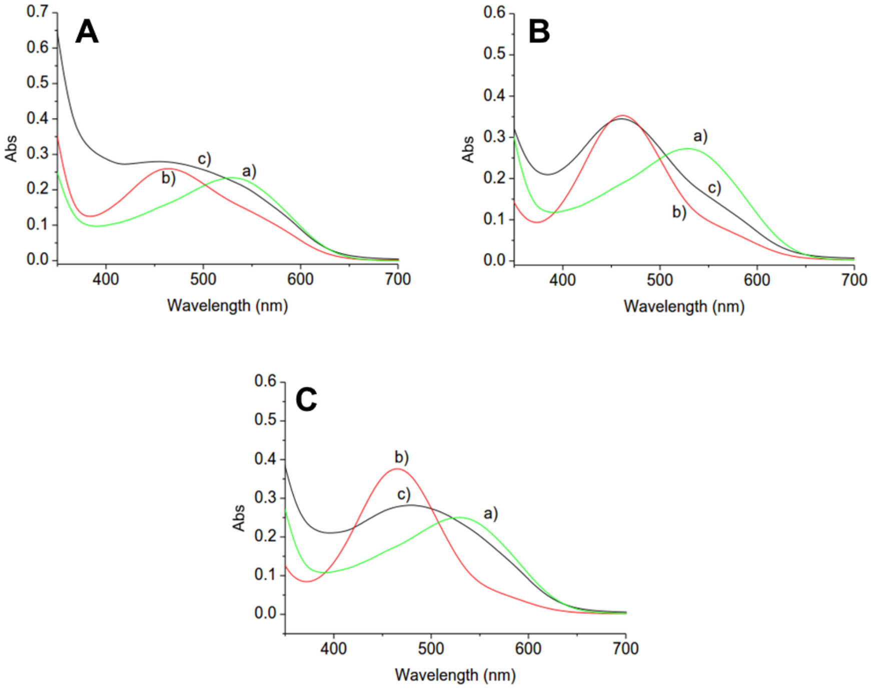

Fig. S2. A: UV-Vis spectra of a) **4** (45 µM), b) **4** (45 µM) and **10** (1.2 mM), and c) **4** (45 µM), **10** (1.2 mM) and **5** (1.6 mM). B: UV-Vis spectra of a) **4** (45 µM), b) **4** (45 µM) and **11** (520 µM), and c) **4** (45 µM), **11** (520 µM) and **5** (1.6 mM). C: UV-Vis spectra of a) **4** (45 µM), b) **4** (45 µM) and **12** (300 µM), and c) **4** (45 µM), **12** (300 µM) and **5** (800 µM).

Table S1. Dissociation constants K_D for boronic esters obtained from boronic acids **10-12** and catechols **1-9**[a]

Boronic acid	K_D (mM)								
	**1**	**2**[b]	**3**	**4**	**5**[b]	**6**	**7**[b]	**8**[b]	**9**[b]
**10**	0.28	0.62	1.04[c]	1.46[c]	2.50	4.20[c]	5.70	6.90	33.0
**11**	0.21	0.34	0.31	0.17	0.50	0.10	1.10	1.40	1.10
**12**	0.03	0.08	0.17[c]	0.09[c]	0.10	0.03[c]	0.26	0.30	0.30

[a]From changes in absorption in MeOH/H_2O 3:1 (100 mM HEPES, pH 7.8) upon direct or [b]reverse titration with **4**. [c]From ref. S1.

4. Orthogonal dynamic covalent bonds in solution

All experiments were run using DMSO-d_6 dried over molecular sieves (3 Å), at rt.

Condition A: DMSO-d_6, 10% D_2O, 2% DIPEA.

Condition B: DMSO-d_6, 1.0 mM **19**, 1.5 mM TFA.

Condition C: DMSO-d_6, 2.0 mM **23**, 2.0 mM TEA.

Condition D: DMSO-d_6.

Condition B*: DMSO-d_6, 11.0 mM aniline, 11.5 mM TFA.

Stock solution B: **19** (8 mg, 50 µmol) and TFA (5.8 µL, 76 µmol) were dissolved in DMSO-d_6 (0.50 mL).

Stock solution C: **23** (1.2 µL, 10 µmol) and TEA (1.4 µL, 10 µmol) were dissolved in DMSO-d_6 (0.50 mL).

Stock solution B*: aniline (50 µL, 55 µmol) and TFA (44 µL, 580 µmol) were dissolved in DMSO-d_6 (0.50 mL).

In most experiments, TES (20 mM) was also included to serve as an internal standard.

4.1. Boronate ester exchange in solution

Boronate ester stock solution: To a solution of **10** (9 mg, 40 μmol) in DMSO-d_6 (1.80 mL), was added **1** (11 mg, 44 μmol) and the solution was shaken. After verifying the complete formation of **13** by ^{1}H NMR spectroscopy, **8** (12 mg, 44 μmol) was added to the solution and after shaking, the solution was used for the exchange experiments.

Condition A: To 0.45 mL of the boronate ester stock solution in an NMR tube, was added 10.0 μL of DIPEA and 50 μL of D_2O. After shaking, the solution was subjected to ^{1}H NMR spectroscopy to monitor the evolution of the exchange processes (Figs. S4, S3 ●, 3A ●).

Condition B: To 0.45 mL of the boronate ester stock solution in an NMR tube, was added 5.0 μL of stock solution B and the mixture was immediately shaken, as to avoid a higher local concentration in the sample. After addition of 45 μL of DMSO-d_6, the solution was subjected to ^{1}H NMR spectroscopy to monitor the evolution of the exchange processes (Figs. S6, S3 ▲, 3B ●).

Condition C: To 0.45 mL of the boronate ester stock solution in an NMR tube, was added 50 μL of stock solution C. After shaking, the solution was subjected to ^{1}H NMR spectroscopy to monitor the evolution of the exchange processes (Figs. S7, S3 ■, 3C ●).

Condition D: To 0.45 mL of the boronate ester stock solution in an NMR tube was added 50 μL of DMSO-d_6. The solution was then subjected to ^{1}H NMR spectroscopy to monitor the evolution of the exchange processes (Fig. S8, S3 ♦).

Condition B*: To 0.45 mL of the boronate ester stock solution in an NMR tube, was added 5.0 μL of stock solution B* and the mixture was shaken. After addition of 45 μL of DMSO-d_6, the solution was subjected to ^{1}H NMR spectroscopy to monitor the evolution of the exchange processes (Fig. S19).

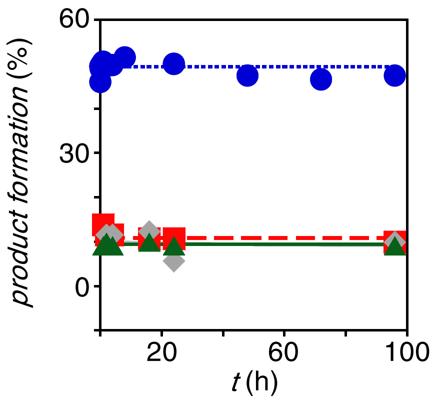

Fig. S3. Exchange kinetics for **13** and **8** in condition **A** (●), condition **B** (▲), condition **C** (■) and condition **D** (control, ♦).

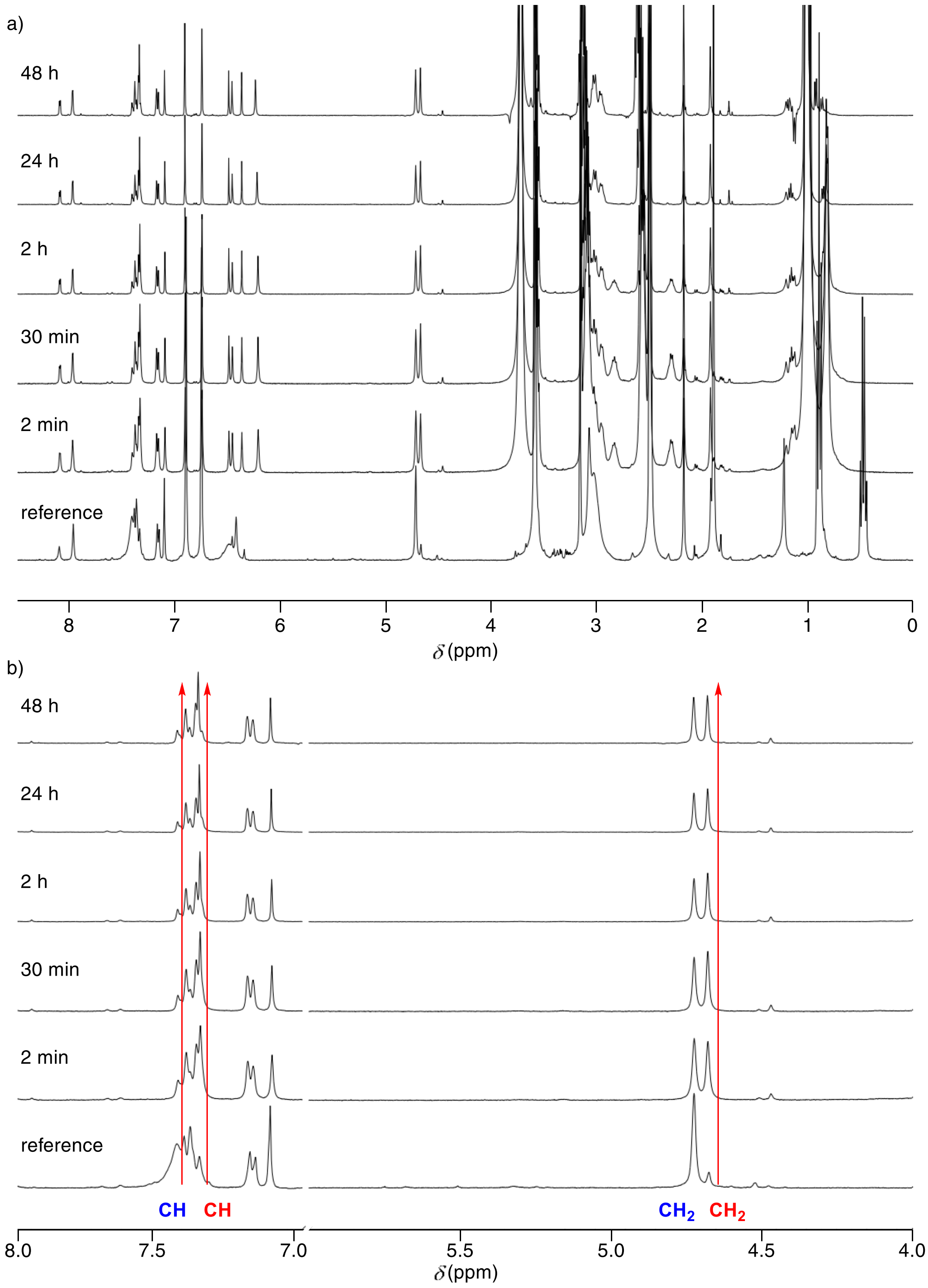

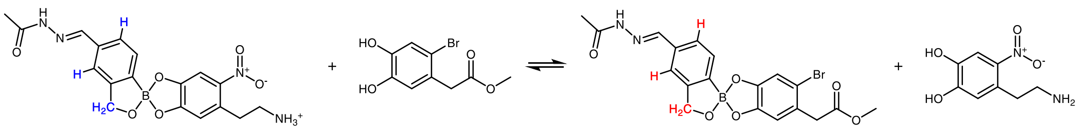

13 8 14 1

Fig. S4. a) ^{1}H NMR spectra of a mixture of **13** (20 mM) and **8** (22 mM) in condition **A** (DMSO-d_6, 10% D_2O, 2% DIPEA), b) zoom of the diagnostic peaks showing the evolution of the exchange processes. In blue is shown the position of the starting material, while red indicates the product of the exchange. The reference spectrum is taken from fig. S8.

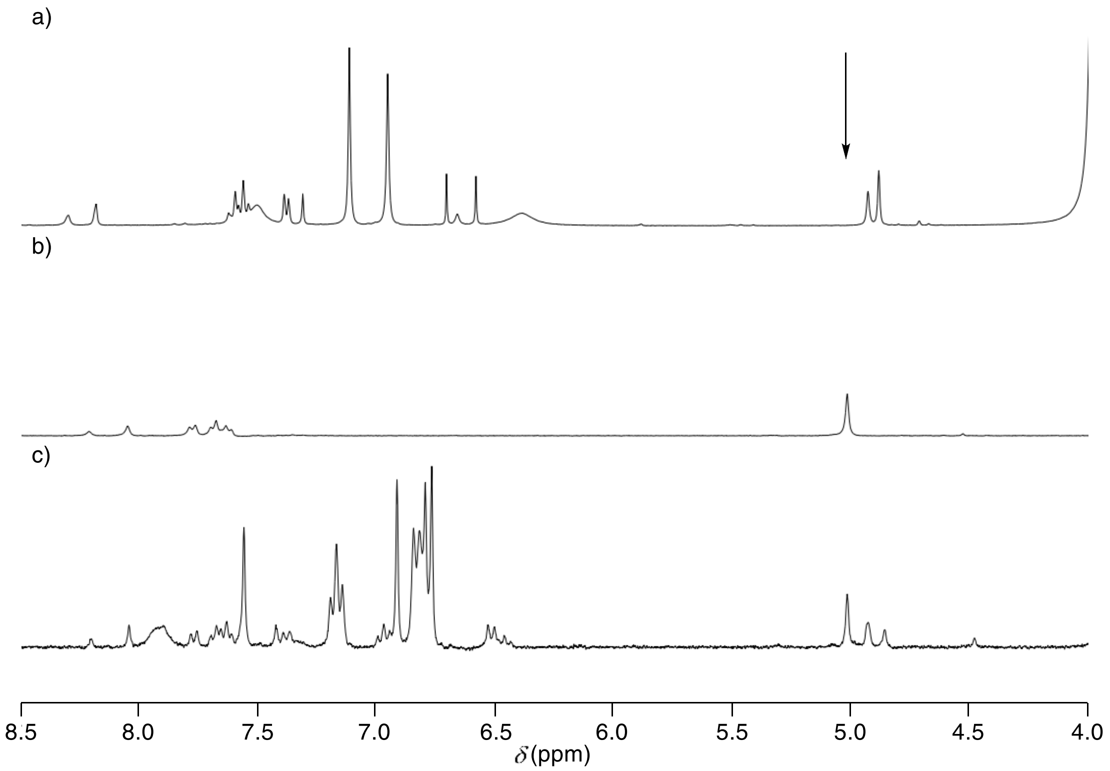

Fig. S5. a) ^{1}H NMR spectrum of a mixture of **13** (20 mM) and **8** (22 mM) after 16 h in condition **A** (DMSO-d_6, 10% D_2O, 2% DIPEA), b) ^{1}H NMR spectrum of benzoboroxole **10** in DMSO-d_6, c) ^{1}H NMR spectrum of a partially hydrolysed mixture of **13** (20 mM) and **8** (22 mM) after 18 h in condition **B*** (DMSO-d_6, 11.0 mM aniline, 11.5 mM TFA). The arrow shows the absence of hydrolysis product during exchange in condition **A**.

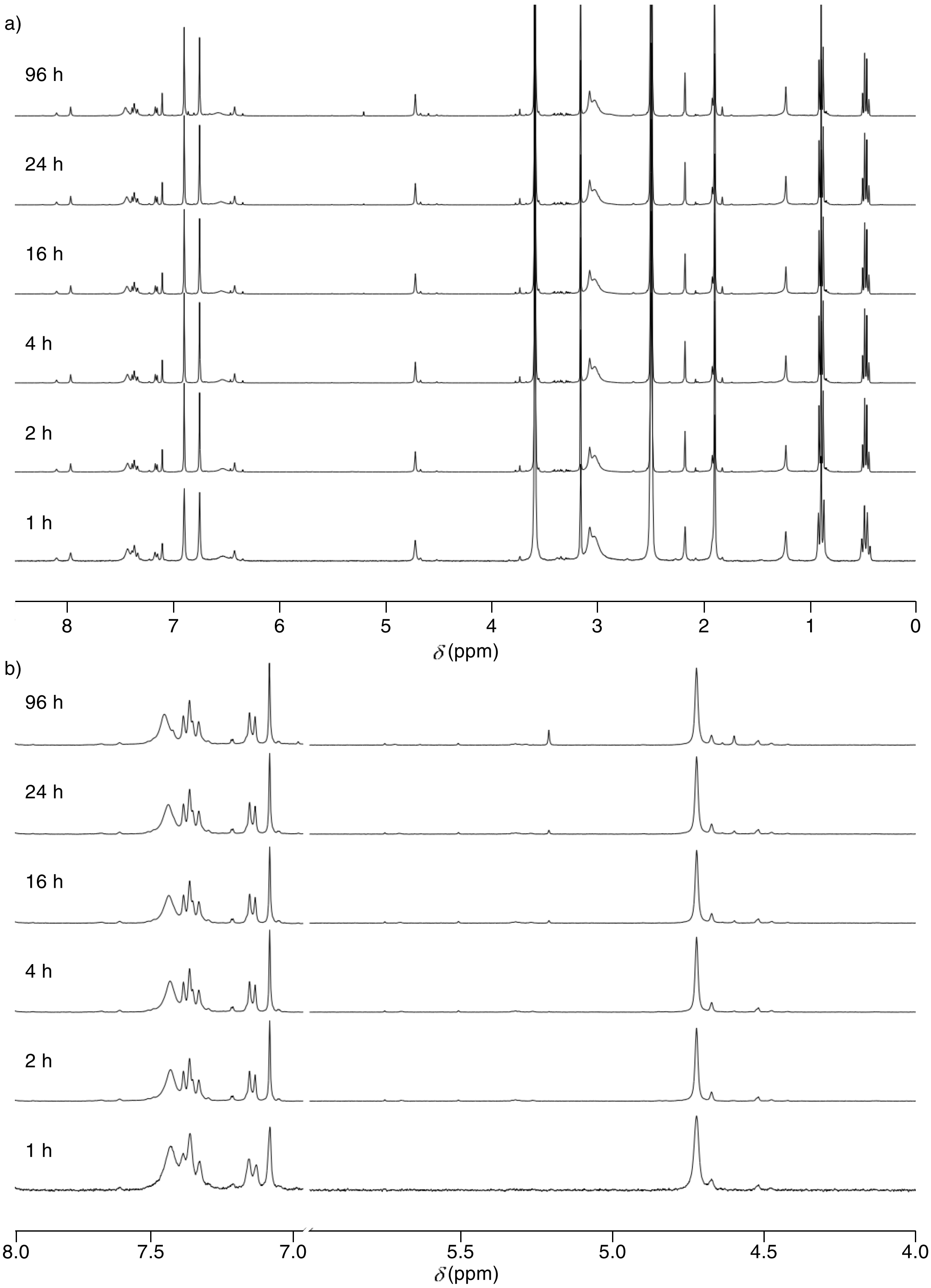

Fig. S6. a) ^{1}H NMR spectra of a mixture of **13** (20 mM), **8** (22 mM) and TES (20 mM) in condition **B** (DMSO-d_6, 1.0 mM **19**, 1.5 mM TFA), b) zoom of the diagnostic peaks showing the lack of evolution of the exchange processes.

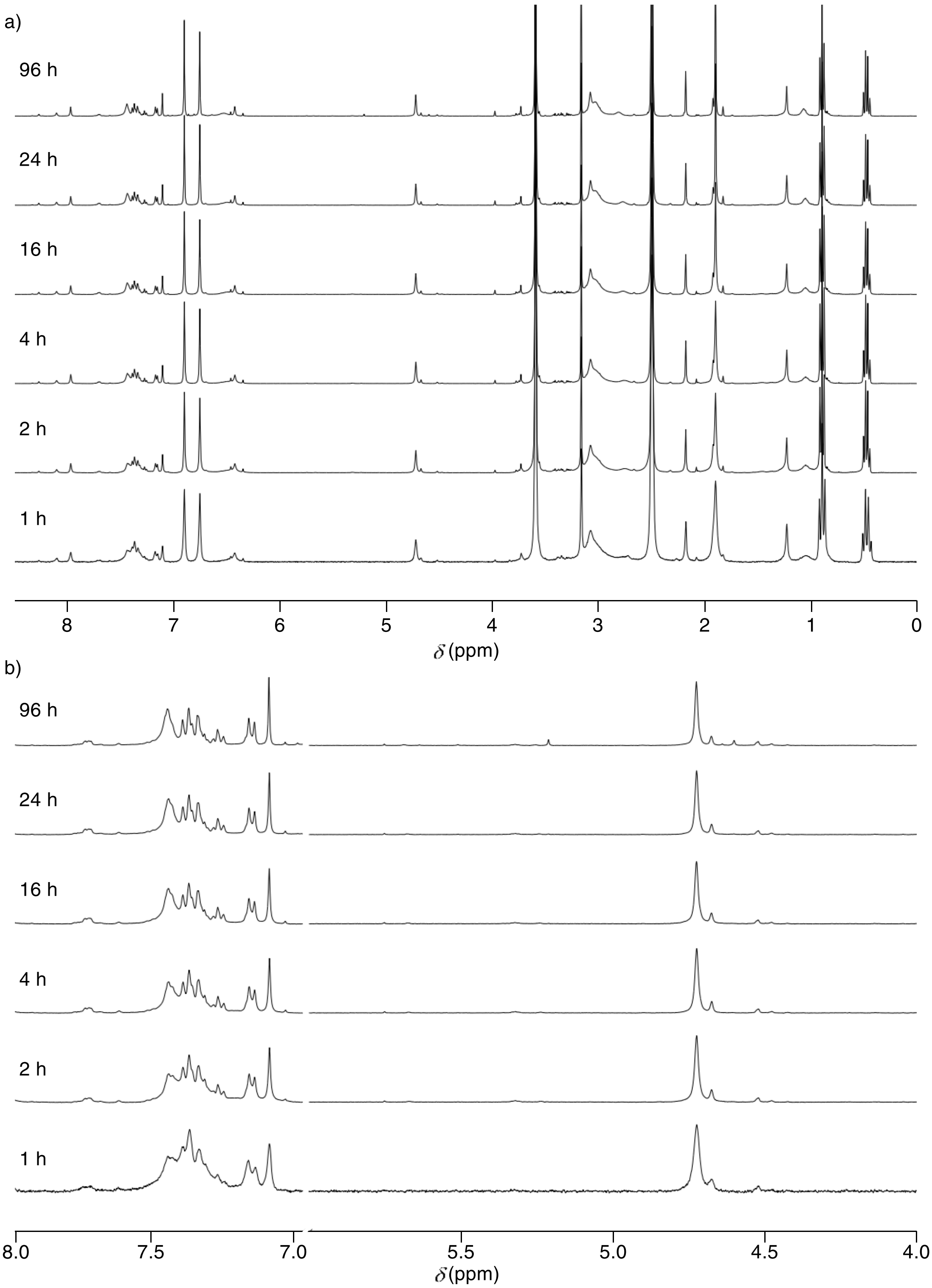

Fig. S7. a) ^{1}H NMR spectra of a mixture of **13** (20 mM), **8** (22 mM) and TES (20 mM) in condition **C** (DMSO-d_6, 2.0 mM **23**, 2.0 mM TEA), b) zoom of the diagnostic peaks showing the lack of evolution of the exchange processes.

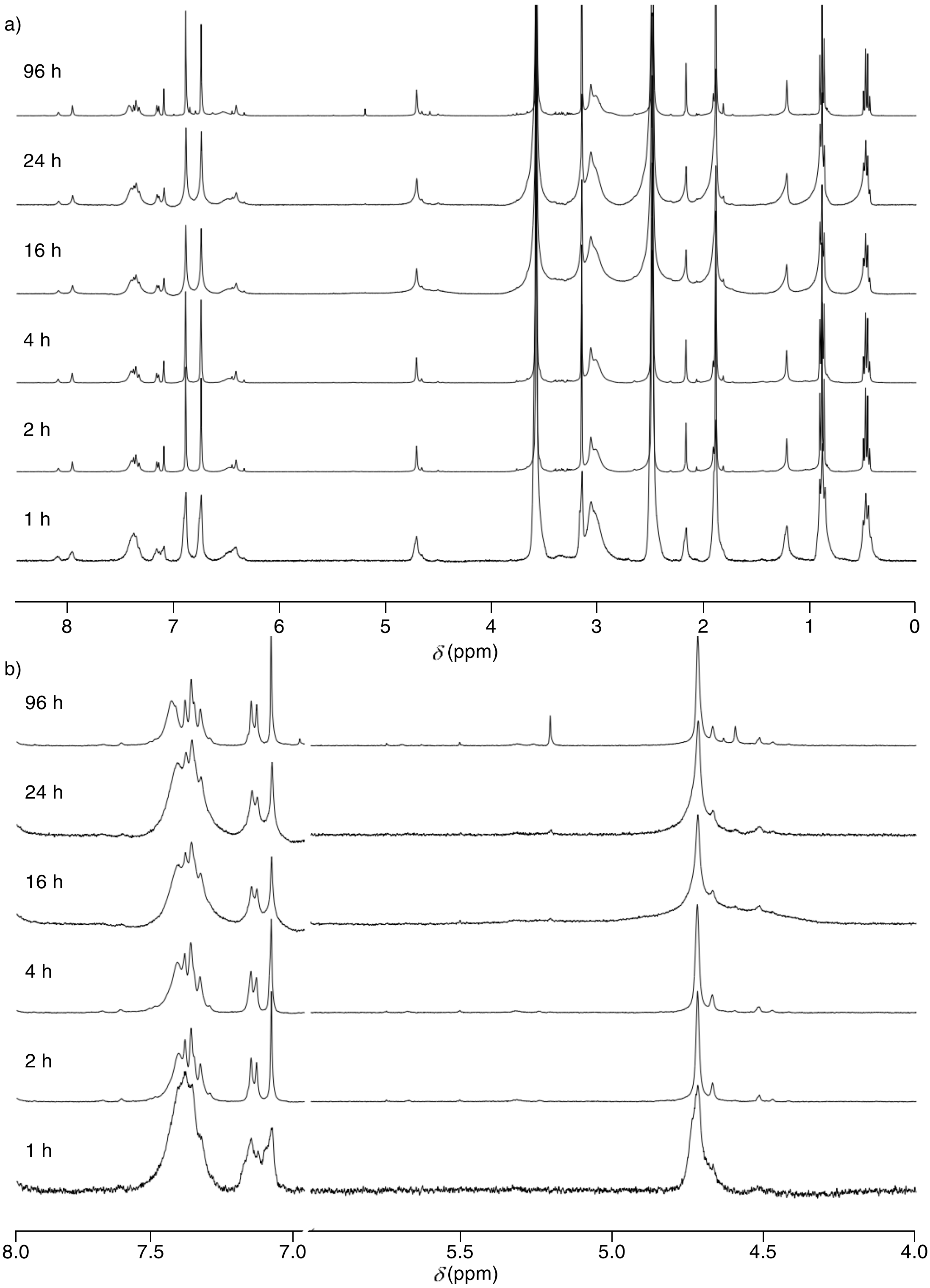

Fig. S8. a) ^{1}H NMR spectra of a mixture of **13** (20 mM), **8** (22 mM) and TES (20 mM) in condition **D** (DMSO-d_6), b) zoom of the diagnostic peaks showing the lack of evolution of the exchange processes.

4.2. Hydrazone exchange in solution

Hydrazone stock solution: To a solution of **16** (3 mg, 40 µmol) in DMSO-d_6 (1.80 mL) was added **15** (9 mg, 40 µmol) and after shaking, the solution was used for the exchange experiments.

Condition A: To 0.45 mL of the hydrazone stock solution in an NMR tube, was added 10.0 µL of DIPEA and 50 µL of D_2O. After shaking, the solution was subjected to ^{1}H NMR spectroscopy to monitor the evolution of the exchange processes (Figs. S11, S9●, 3A ▲).

Condition B: To 0.45 mL of the hydrazone stock solution in an NMR tube, was added 5.0 µL of stock solution B and 45 µL of DMSO-d_6. After shaking, the solution was subjected to ^{1}H NMR spectroscopy to monitor the evolution of the exchange processes (Figs. S10, S9▲, 3B ▲).

Condition C: To 0.45 mL of the hydrazone stock solution in an NMR tube, was added 50 µL of stock solution C. After shaking, the solution was subjected to ^{1}H NMR spectroscopy to monitor the evolution of the exchange processes (Figs. S12, S9 ■, 3C ▲).

Condition D: To 0.45 mL of the hydrazone stock solution in an NMR tube was added 50 µL of DMSO-d_6. The solution was then subjected to ^{1}H NMR spectroscopy to monitor the evolution of the exchange processes (Figs. S13, S9 ♦).

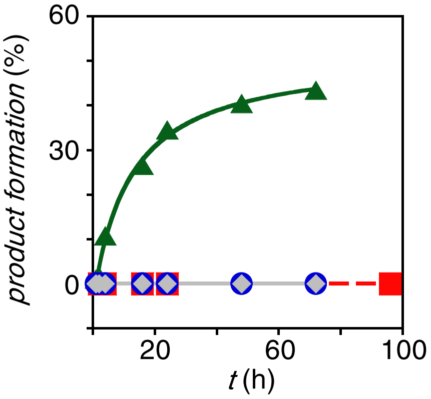

Fig. S9. Exchange kinetics for **15** and **16** in condition **A** (●), condition **B** (▲), condition **C** (■) and condition **D** (control, ♦).

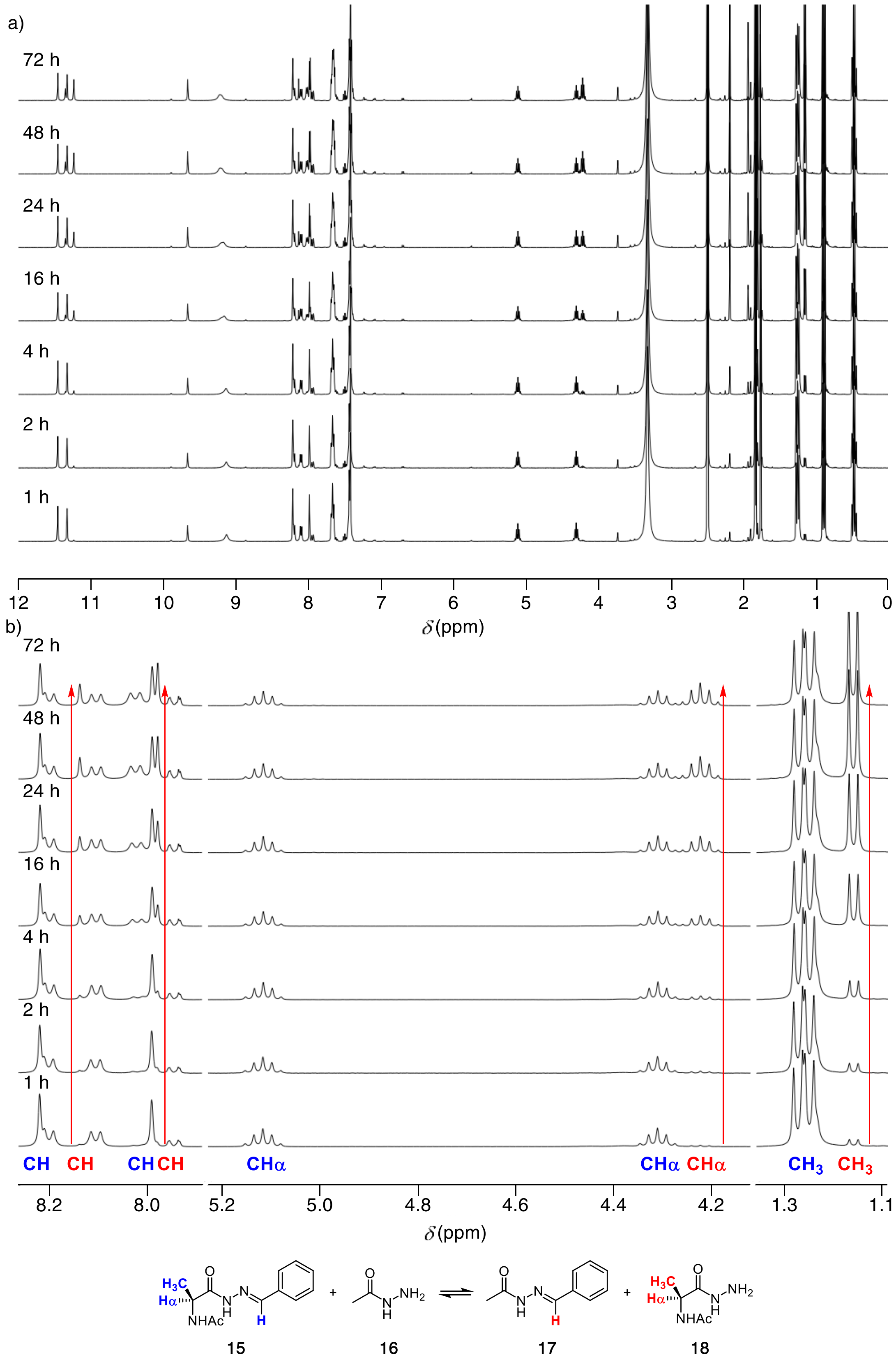

Fig. S10. a) ^{1}H NMR spectra of a mixture of **15** (20 mM), **16** (20 mM) and TES (20 mM) in condition **B** (DMSO-d_6, 1.0 mM **19**, 1.5 mM TFA), b) zoom of the diagnostic peaks showing the evolution of the exchange processes. In blue is shown the position of the starting material, while red indicates the product of the exchange.

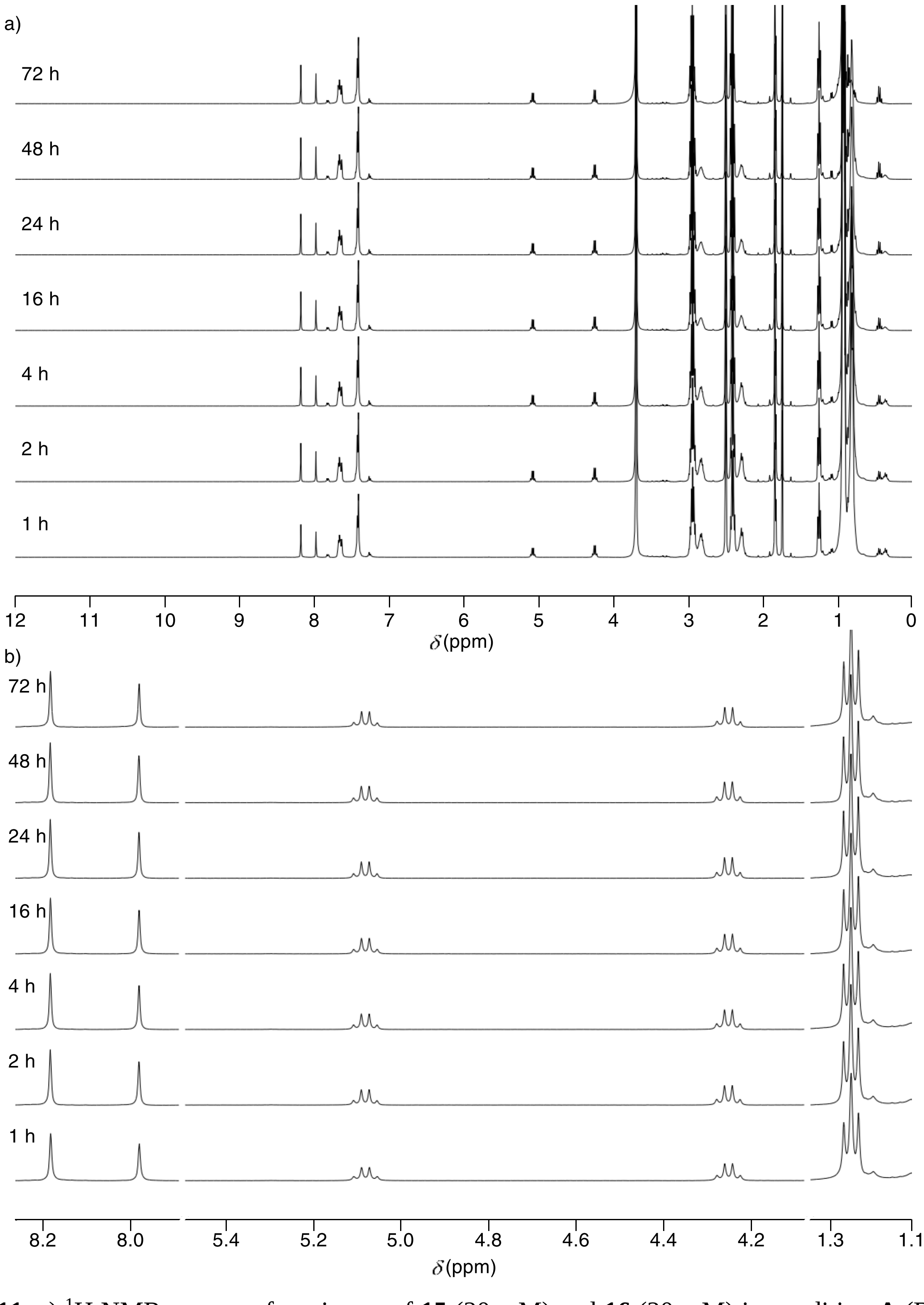

Fig. S11. a) ^{1}H NMR spectra of a mixture of **15** (20 mM) and **16** (20 mM) in condition **A** (DMSO-d_6, 10% D_2O, 2% DIPEA), b) zoom of the diagnostic peaks showing the lack of evolution of the exchange processes.

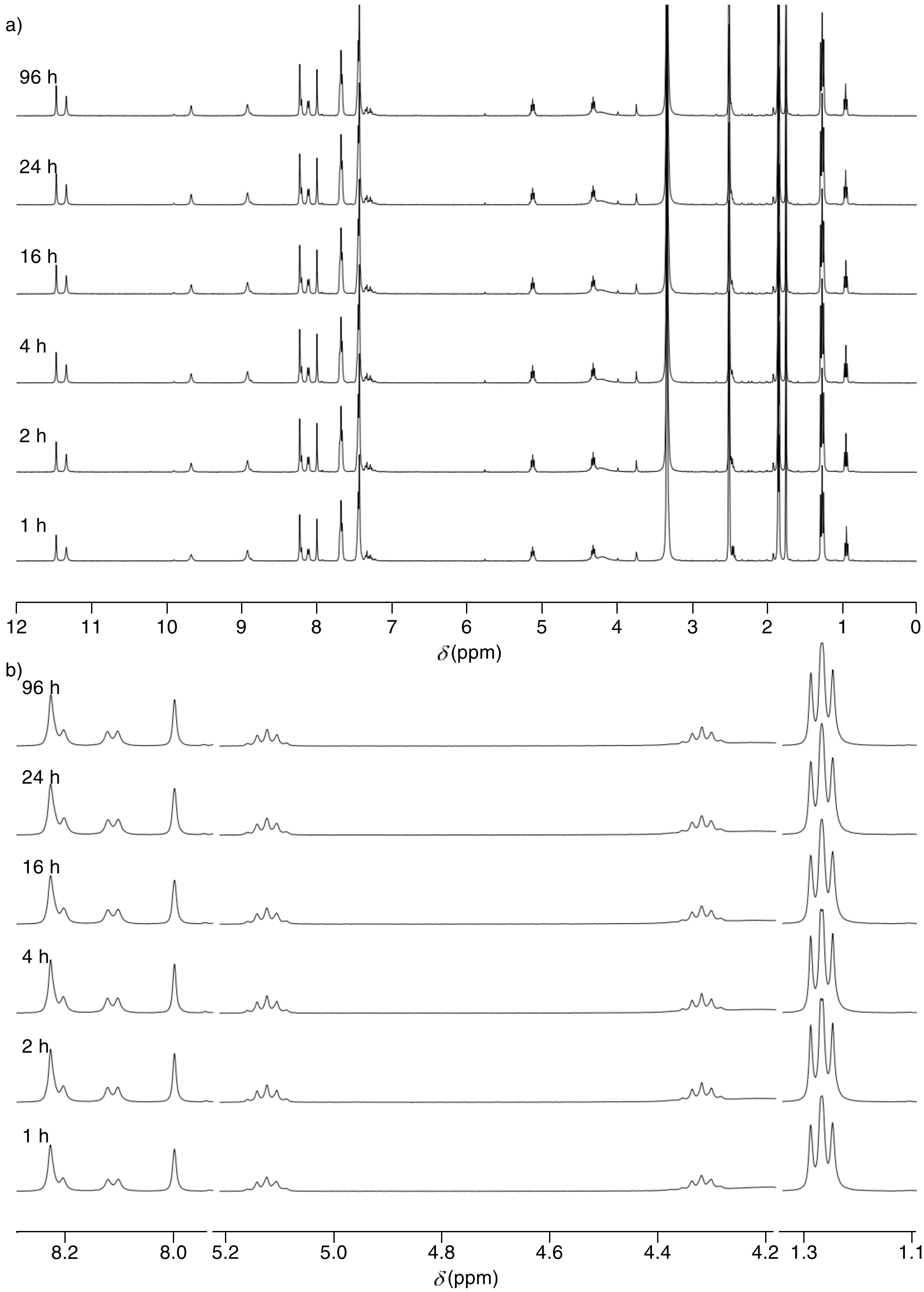

Fig. S12. a) ^{1}H NMR spectra of a mixture of **15** (20 mM) and **16** (20 mM) in condition **C** (DMSO-d_6, 2.0 mM **23**, 2.0 mM TEA), b) zoom of the diagnostic peaks showing the lack of evolution of the exchange processes.

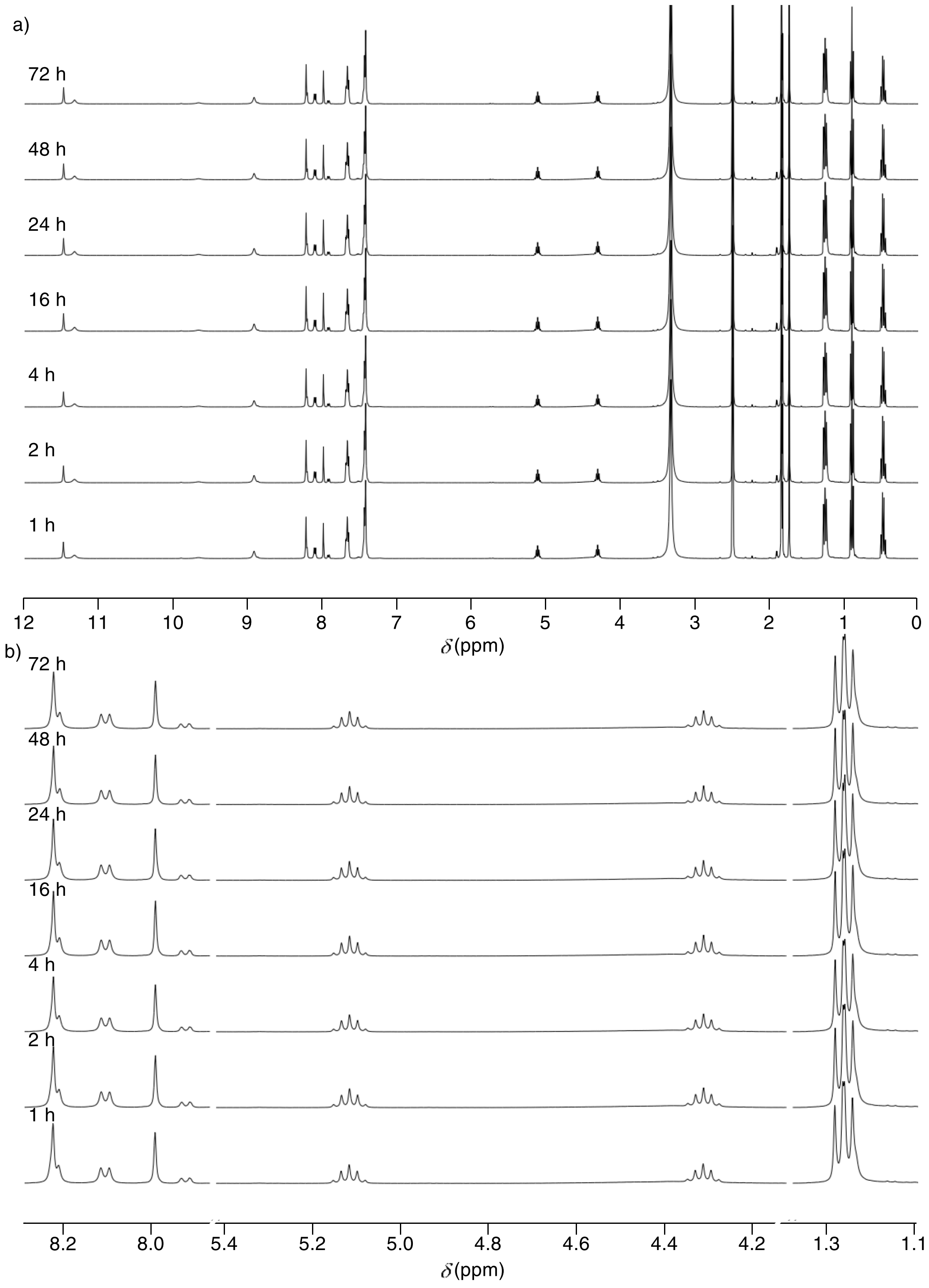

Fig. S13. a) ^{1}H NMR spectra of a mixture of **15** (20 mM), **16** (20 mM) and TES (20 mM) in condition **D** (DMSO-d_6), b) zoom of the diagnostic peaks showing the lack of evolution of the exchange processes.

4.3. Disulfide exchange in solution

Disulfide stock solution: To a solution of **20** (22 mg, 40 µmol) in DMSO-d_6 (1.80 mL) was added **21** (10 mg, 40 µmol) and after shaking, the solution was used for the exchange experiments.

Condition A: To 0.45 mL of the disulfide stock solution in an NMR tube, was added 10.0 µL of DIPEA and 50 µL of D_2O. After shaking, the solution was subjected to ^1H NMR spectroscopy to monitor the evolution of the exchange processes (Figs. S16, S14 ●, 3A ■).

Condition B: To 0.45 mL of the disulfide stock solution in an NMR tube, was added 5.0 µL of stock solution B and 45 µL of DMSO-d_6. After shaking, the solution was subjected to ^1H NMR spectroscopy to monitor the evolution of the exchange processes (Figs. S17, S14 ▲, 3B ■).

Condition C: To 0.45 mL of the disulfide stock solution in an NMR tube, was added 50 µL of stock solution C. After shaking, the solution was subjected to ^1H NMR spectroscopy to monitor the evolution of the exchange processes (Figs. S15, S14 ■, 3C ■).

Condition D: To 0.45 mL of the disulfide stock solution in an NMR tube was added 50 µL of DMSO-d_6. The solution was then subjected to ^1H NMR spectroscopy to monitor the evolution of the exchange processes (Figs. S18, S14 ♦).

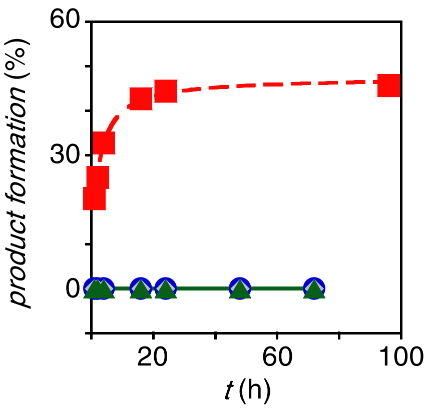

Fig. S14. Exchange kinetics for **20** and **21**, in condition **A** (●), condition **B** (▲), condition **C** (■) and condition **D** (control, ♦).

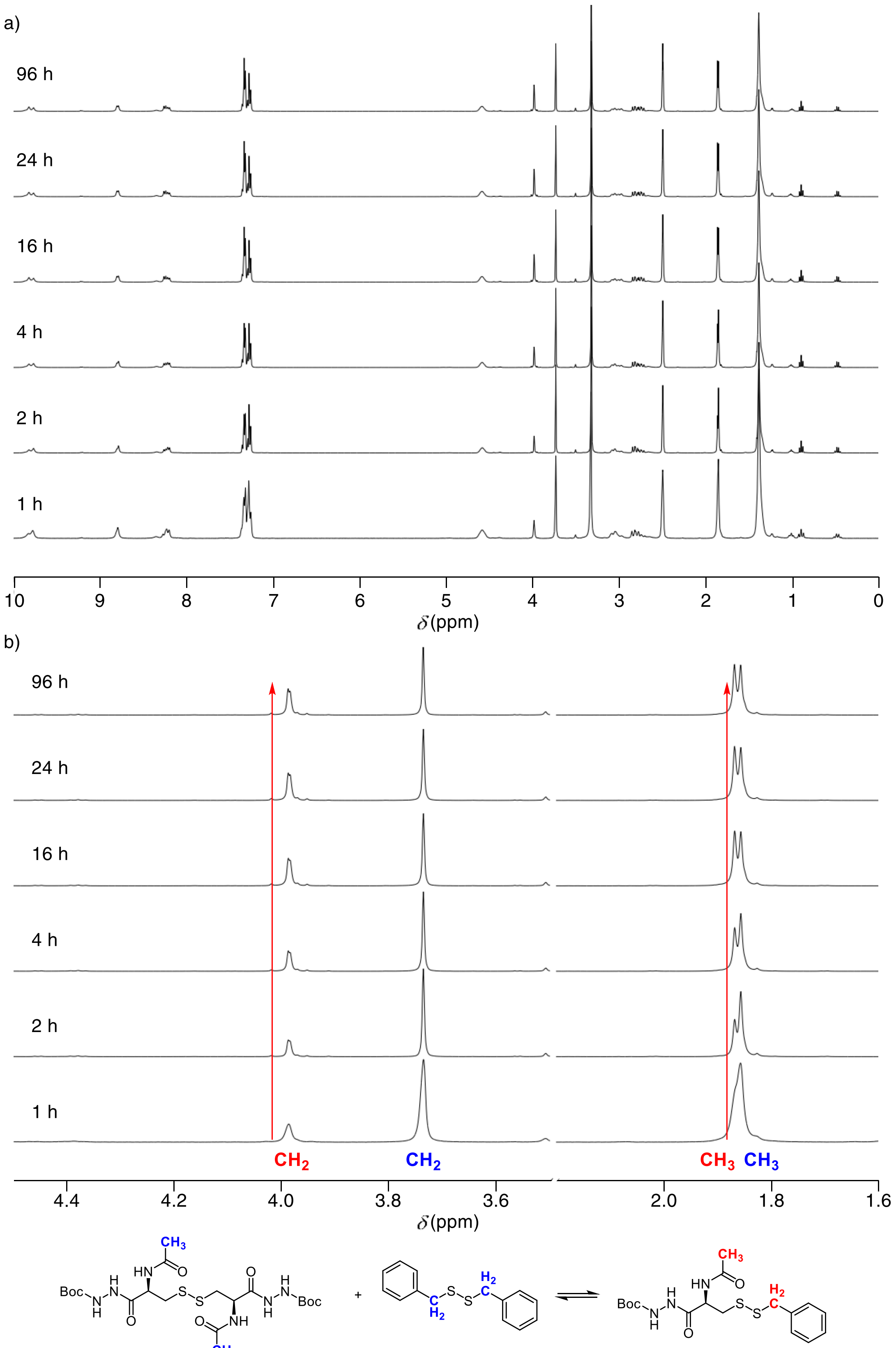

Fig. S15. a) ^{1}H NMR spectra of a mixture of **20** (20 mM), **21** (20 mM) and TES (20 mM) in condition **C** (DMSO-d_6, 2.0 mM **23**, 2.0 mM TEA), b) zoom of the diagnostic peaks showing the evolution of the exchange processes. In blue is shown the position of the starting material, while red indicates the product of the exchange.

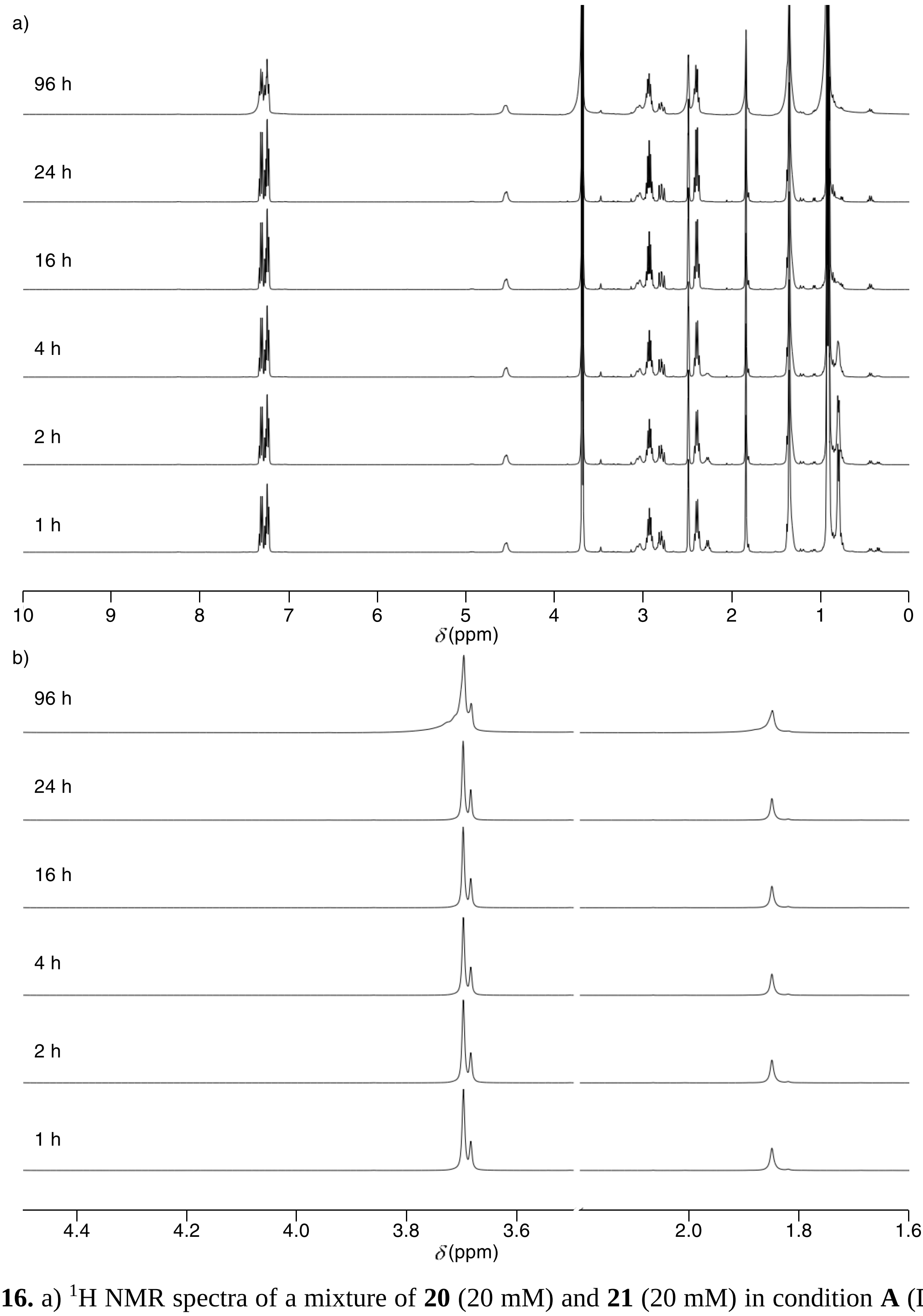

Fig. S16. a) ^{1}H NMR spectra of a mixture of **20** (20 mM) and **21** (20 mM) in condition **A** (DMSO-d_6, 10% D_2O, 2% DIPEA), b) zoom of the diagnostic peaks showing the lack of evolution of the exchange processes. The peak at 3.7 ppm corresponds to the water peak.

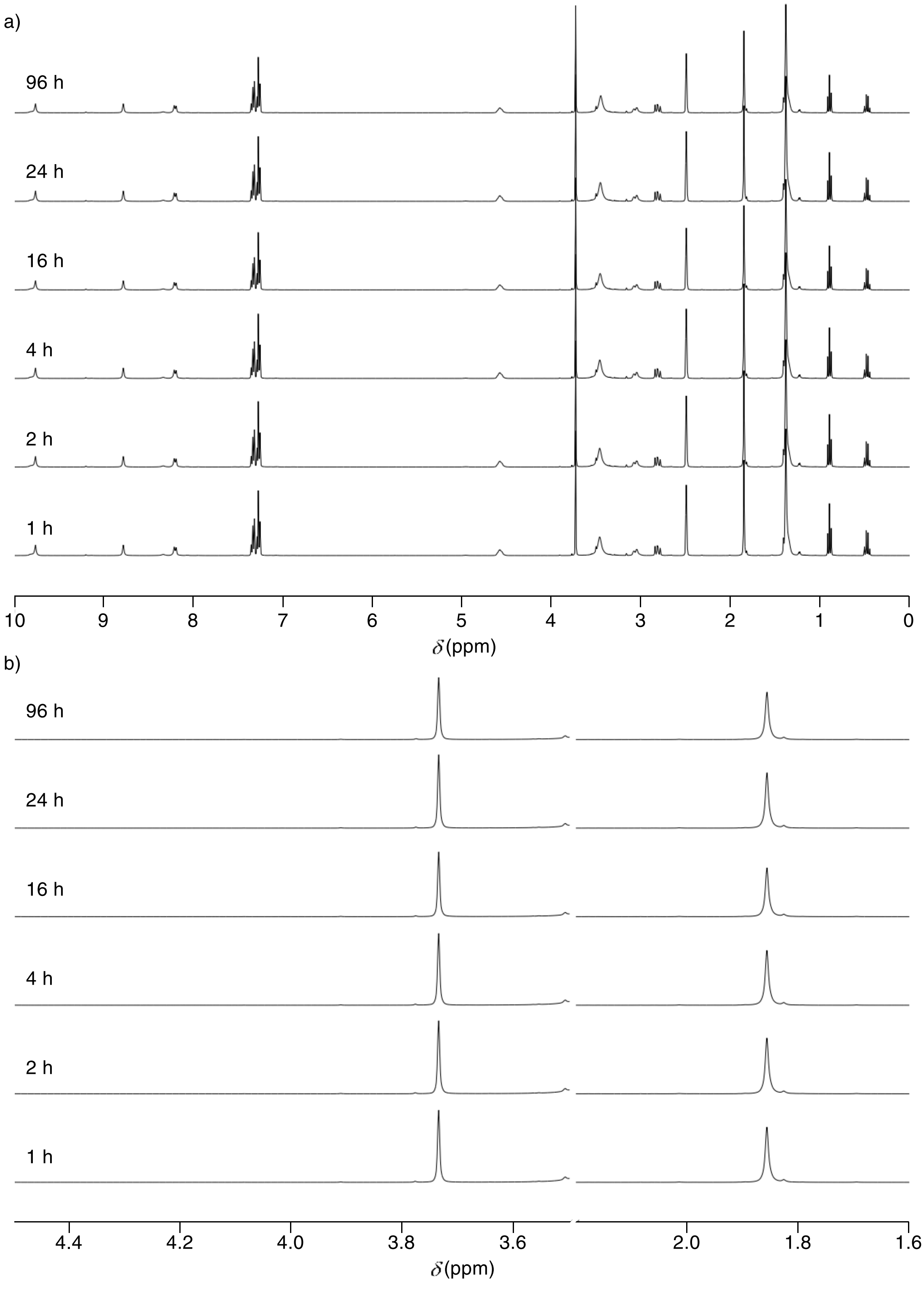

Fig. S17. a) ^{1}H NMR spectra of a mixture of **20** (20 mM), **21** (20 mM) and TES (20 mM) in condition **B** (DMSO-d_6, 1.0 mM **19**, 1.5 mM TFA), b) zoom of the diagnostic peaks showing the lack of evolution of the exchange processes.

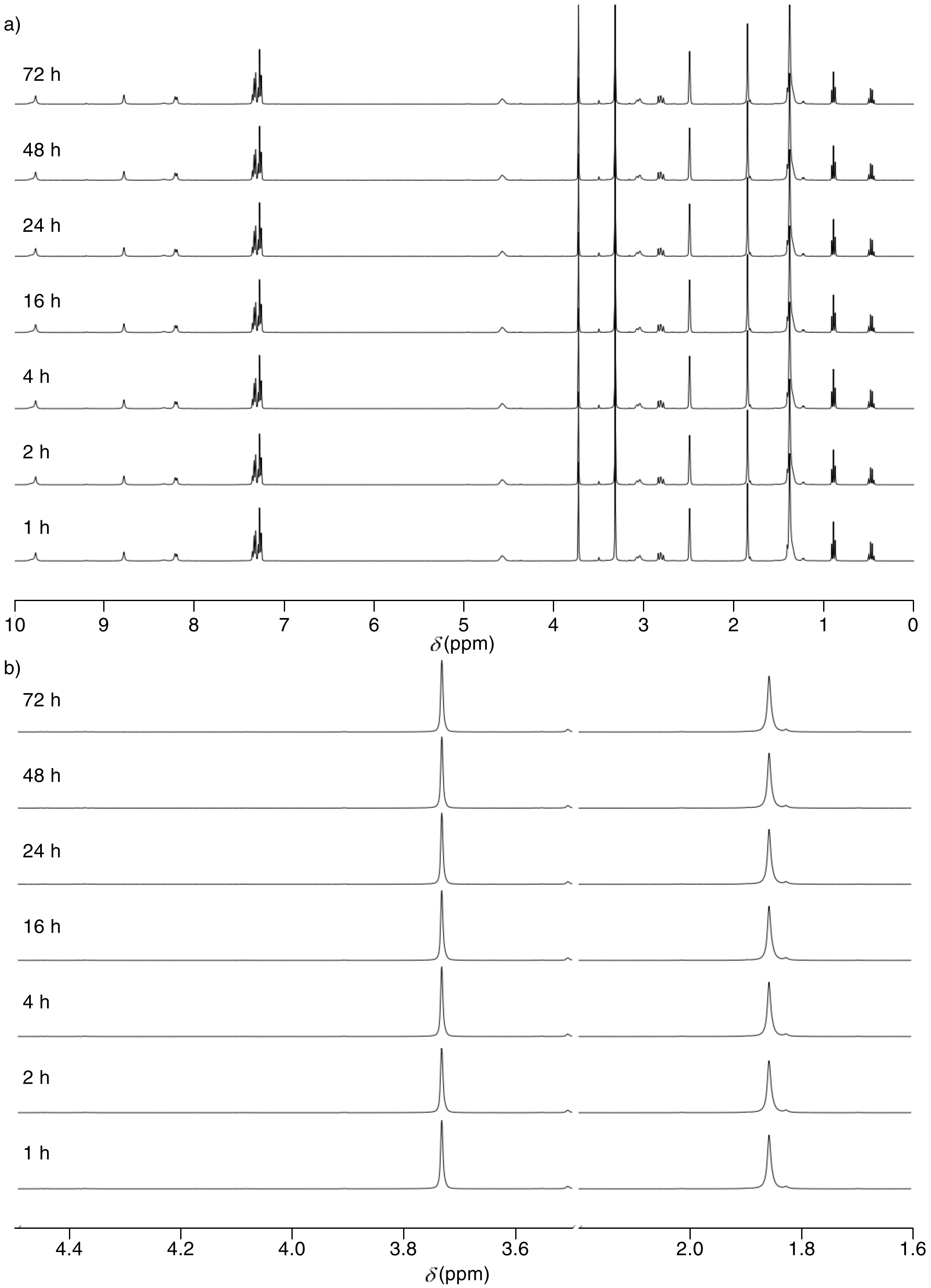

Fig. S18. a) ^{1}H NMR spectra of a mixture of **20** (20 mM), **21** (20 mM) and TES (20 mM) in condition **D** (DMSO-d_6), b) zoom of the diagnostic peaks showing the lack of evolution of the exchange processes.

4.4. Control experiments in solution

4.4.1. Non-orthogonal hydrazone exchange conditions

Classical hydrazone exchange conditions involve the use of acid and often the use of aniline as a catalyst. In an attempt to identify mild conditions in which the exchange takes place, condition **B*** (DMSO-d_6, 11.0 mM aniline, 11.5 mM TFA) was found, resulting in hydrazone exchange. However, the concentrations of acid needed in these conditions are excessive and result in the hydrolysis of boronate esters (Fig. S19), showing the incompatibility of aniline catalysis with boronate esters and thus the necessity of an alternative catalyst.

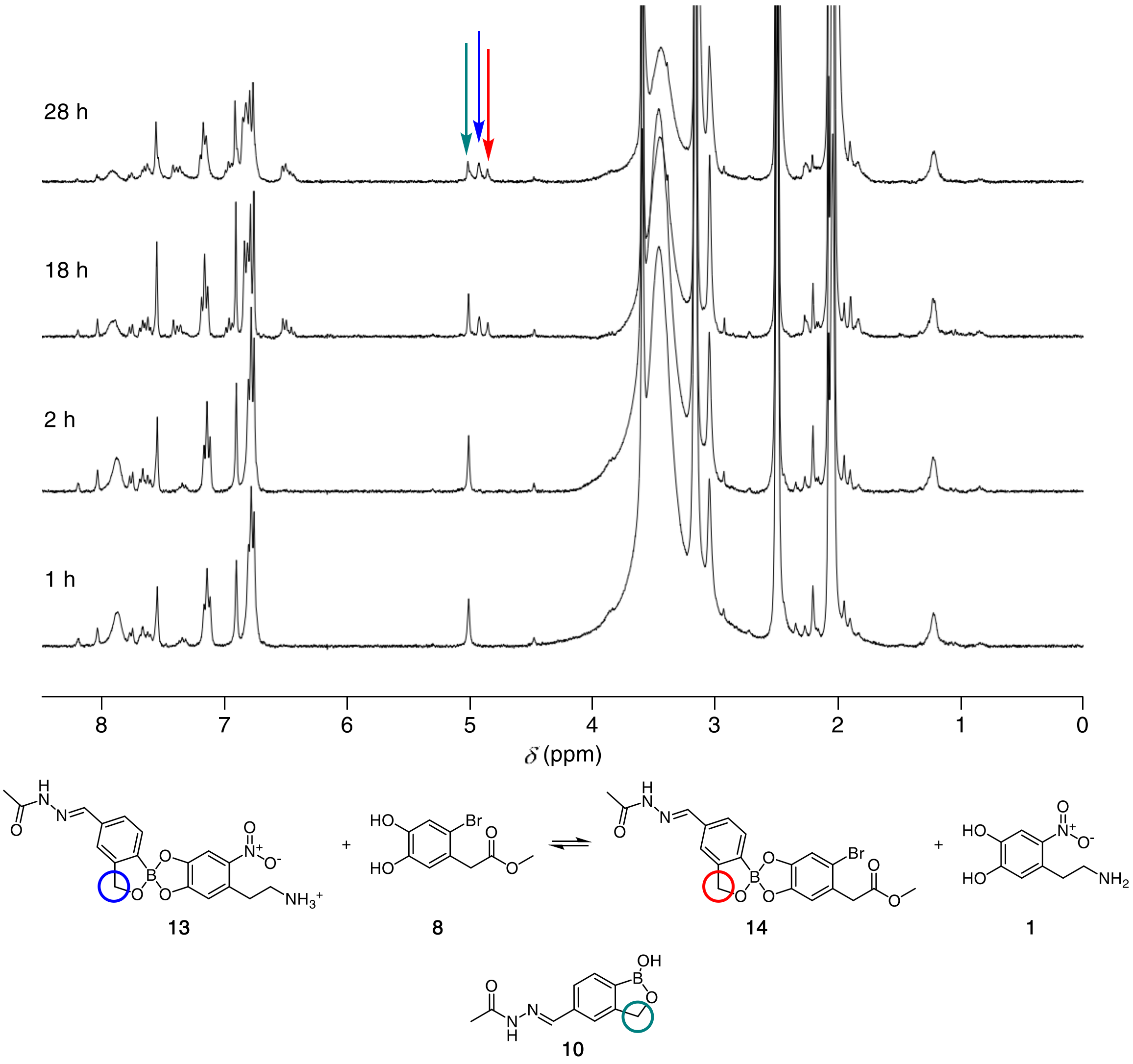

Fig. S19. ^{1}H NMR spectra of a mixture of **13** (20 mM) and **8** (22 mM) in condition **B*** (DMSO-d_6, 11.0 mM aniline, 11.5 mM TFA). After 1 h, there is complete hydrolysis of the boronate ester **13** (blue arrow) into benzoboroxole **10** (green arrow). After 18 h, the boronate esters **13** and **14** (red arrow) are seen to slowly form. This shows the incompatibility of hydrazone exchange conditions **B*** for boronate ester **13**.

4.4.2. Non-orthogonal boronate esters

To test the orthogonality of other boronate esters, boronate ester **36** was synthesized and tested with **8** in condition **B**. In these conditions, significant exchange was observed, confirming the non-orthogonal character of these exchange partners in hydrazone exchange condition **B**.

Boronate ester 36 stock solution: To a solution of **10** (2 mg, 10 µmol) in DMSO-d_6 (0.45 mL), was added **5** (3 mg, 10 µmol) and the solution was shaken. After verifying the complete formation of **36** by ^{1}H NMR spectroscopy, **8** (3 mg, 10 µmol) was added to the solution and after shaking, the solution was used for the exchange experiments.

Boronate ester 36 in condition B: To 0.45 mL of the boronate ester **36** stock solution in an NMR tube, was added 5.0 µL of stock solution **B** and the mixture was shaken. After addition of 45 µL of DMSO-d_6, the solution was subjected to ^{1}H NMR spectroscopy to monitor the evolution of the exchange processes (Figs. S20 ♦, S21).

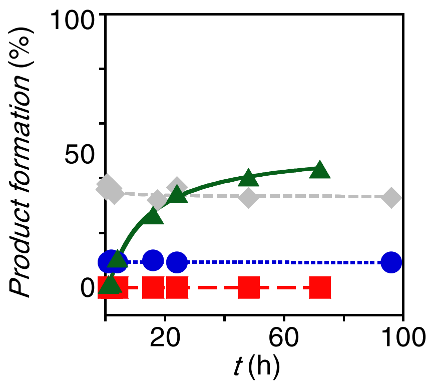

Fig. S20. Exchange kinetics for boronate ester **13** and **8** (●), boronate ester **36** and **8** (♦), hydrazone **15** and **16** (▲) and disulfides **20** and **21** (■) in condition **B**.

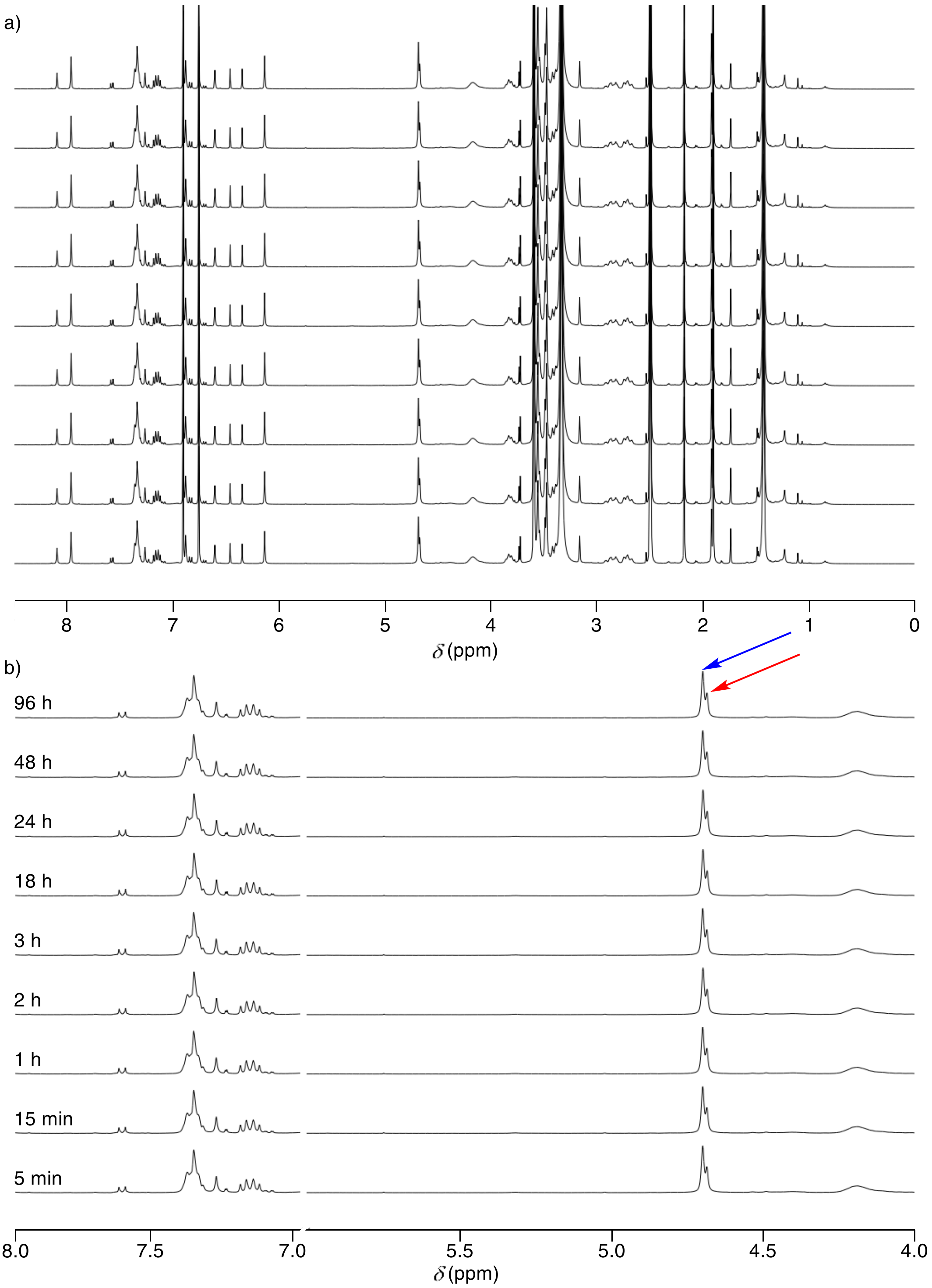

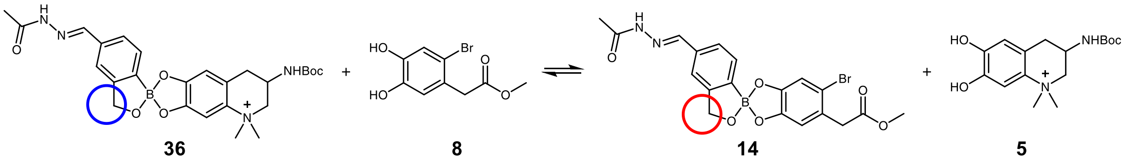

Fig. S21. a) ^{1}H NMR spectra of a mixture of **36** (20 mM) and **8** (22 mM) in condition **B** (DMSO-d_6, 1.0 mM **19**, 1.5 mM TFA), b) zoom of the diagnostic peaks. A significant amount of **14** (red arrow), shows that even in mild condition **B**, **36** undergoes exchange with **8**. Contrary to **13** (Fig. S6), **36** is not orthogonal to hydrazone exchange under condition **B**.

5. Orthogonal dynamic covalent bonds on surfaces

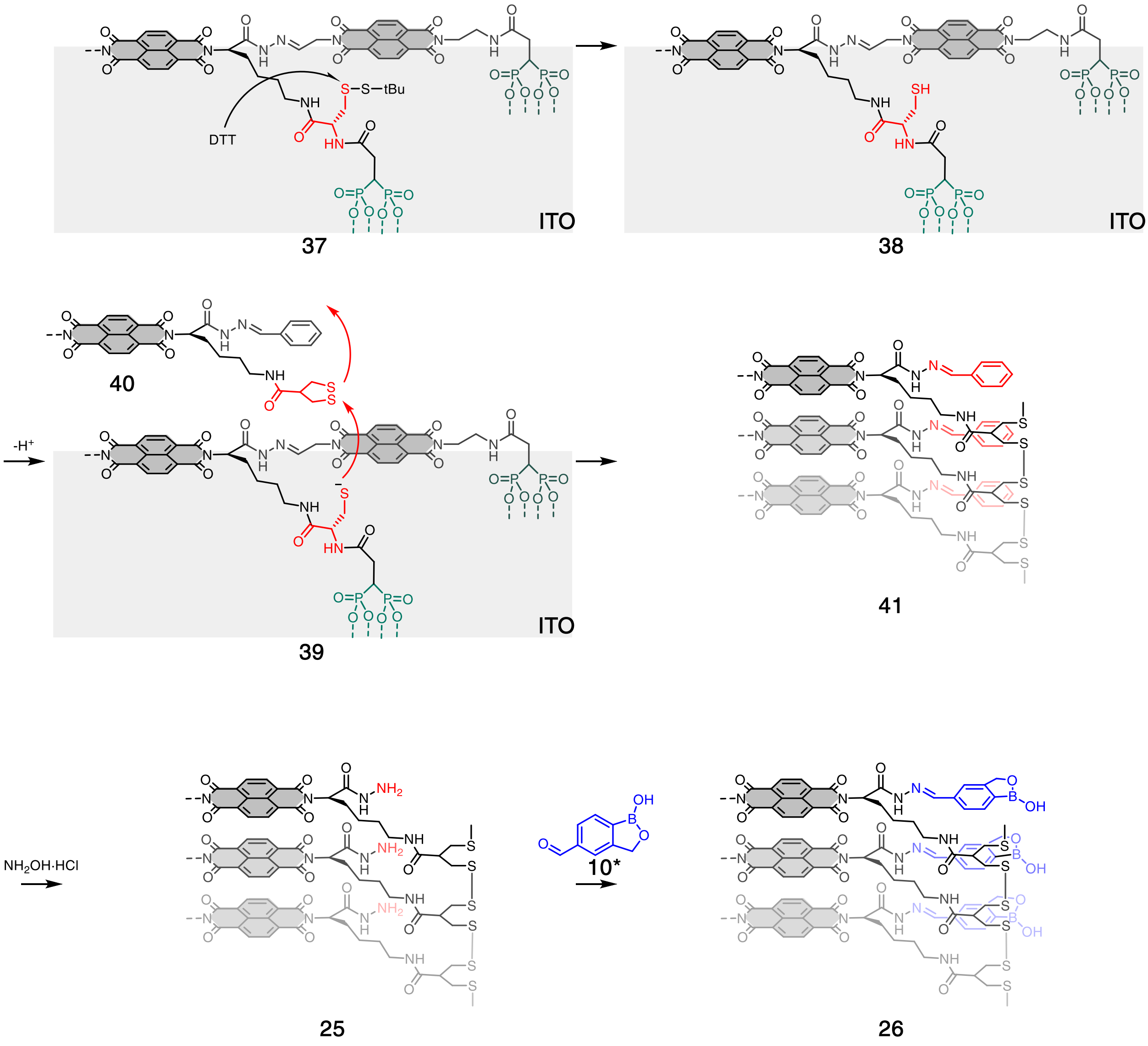

Fig. S22. Schematic description of the synthesis of architecture **26**. Briefly, after formation of a SAM, activation of the "initiator" **37** with DTT, results in thiol **38**. After deprotonation, thiolate **39** starts a ring-opening disulfide-exchange polymerization with the "propagator" **40** resulting in architecture **41**. Treatment with $NH_2OH{\cdot}HCl$ yields architecture **25**, which then condenses with **10*** to yield **26**.

Formation of architecture 24: ITO electrodes **26** were prepared as described in ref. S1 and placed in a solution of **1** (50 mM) in DMSO with 2% DIPEA (v/v) at rt for 24 h. The resulting electrodes were quickly rinsed with EtOH and dried under a flow of air.

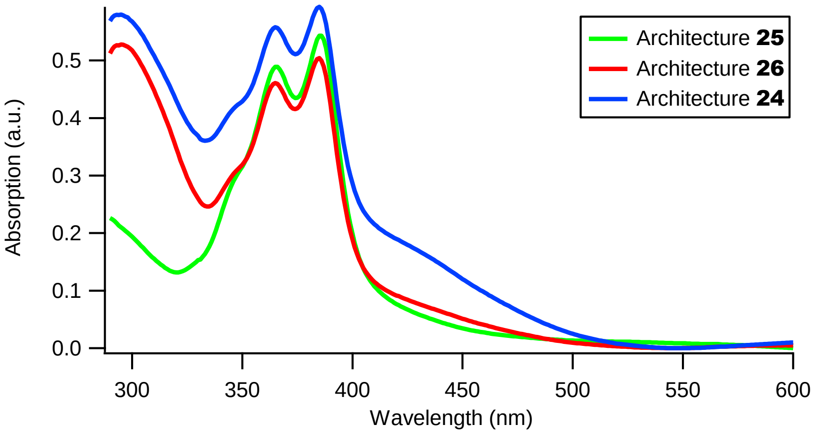

Fig. S23. UV-Vis spectra of architectures **25** (green, after treatment with NH_2OH for 24 h at 40 °C), **26** (red, after treatment with a solution of **41** for 24 h at rt) and **24** (blue, after treatment with a solution of **1** for 24 h at rt).

To determine TSA yield η, the following extinction coefficients were used: [S1]

$$\textbf{10}: \varepsilon_{311} = 14.3\ \text{mM}^{-1}\text{cm}^{-1}$$

$$\textbf{13}: \varepsilon_{423} = 5.5\ \text{mM}^{-1}\text{cm}^{-1}$$

TSA yield η was calculated as follows:

$$\eta = [\Delta A_{423}\ (\textbf{26}\rightarrow\textbf{24}) / \varepsilon_{423}(\textbf{13})]\ /\ [\Delta A_{311}\ (\textbf{25}\rightarrow\textbf{26}) / \varepsilon_{311}(\textbf{10})]$$

$$= [0.097/5.5]\ /\ [0.287/14.3]$$

$$= 0.88$$

Hydrazone exchange on surfaces: 5 electrodes **24** were immersed in 0.1 mL each of a solution of **16** (20 mM), **19** (1.0 mM) and TFA (1.5 mM) in DMSO-d_6 for 7 h at 40 °C. Afterwards, 5 more electrodes **24** were immersed in the same solutions for 7 h at 40 °C. Each solution was used twice to increase the concentration of exchange product in solution. The solutions were combined in an NMR tube and subjected to ^{1}H NMR spectroscopy.

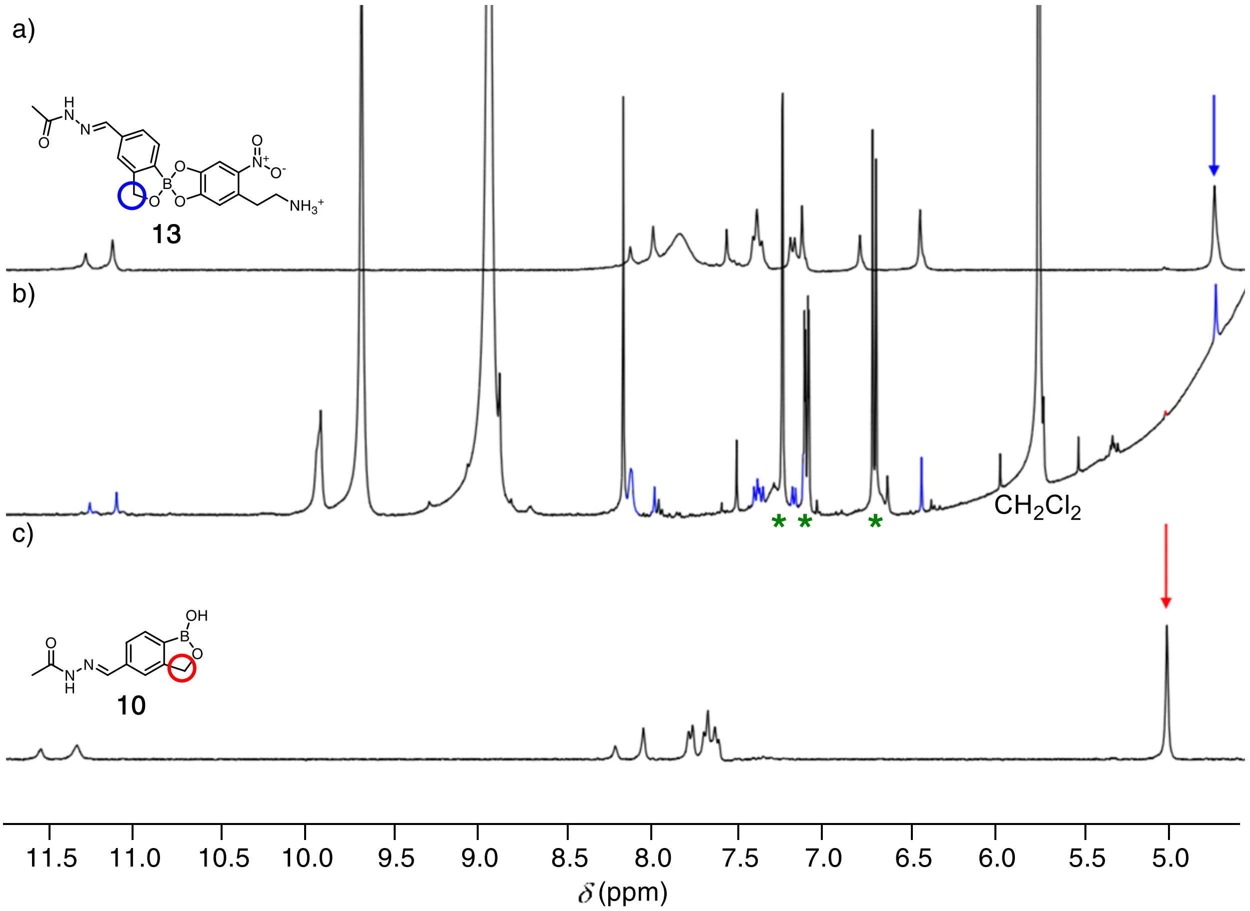

Fig. S24. ^{1}H NMR spectra of a) boronate ester **13** in DMSO-d_6, b) a solution of **19** (1.0 mM) and TFA (1.5 mM) in DMSO-d_6 (condition **B**) after utilization for hydrazone exchange on SOSIP-TSE architecture **24** and c) boronic acid **10** (hydrolysis product) in DMSO-d_6. The raising baseline of b) is due to the peak of water. Peaks assigned to **19** are marked with a green asterisk.

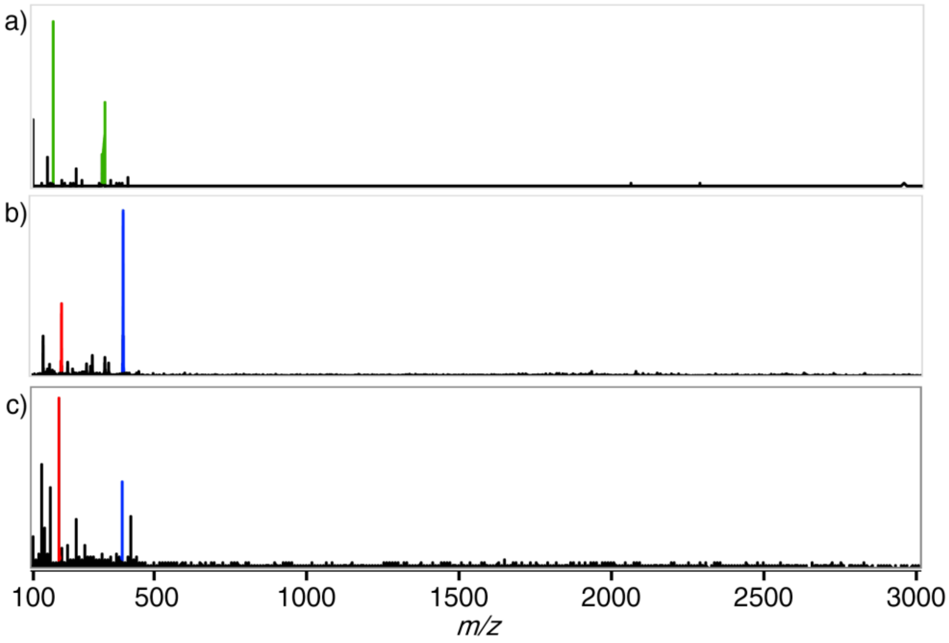

Fig. S25. Full range of ESI-MS (negative mode) of a) catalyst **19** (*m/z* 166 ([M-H]$^-$), 333 ([2M-H]$^-$)), b) **13** (*m/z* 397 ([M-H]$^-$)) and catechol **1** (*m/z* 197 ([M-H]$^-$)), and c) the solution obtained from incubation of multicomponent surface architecture **24** with hydrazide **16** and 1.0 mM catalyst **19** in DMSO-d_6, 1.5 mM TFA, for 7 h at 40 °C (**10**: *m/z* 217 ([M-H]$^-$)).

6. Supporting references

S1. K.-D. Zhang and S. Matile, *Angew. Chem. Int. Ed.*, 2015, **54**, 8980–8983.

S2. D. Larsen, M. Pittelkow, S. Karmakar and E. T. Kool, *Org. Lett.*, 2015, **17**, 274–277.

S3. R. Berger, P. M. A. Rabbat and J. L. Leighton, *J. Am. Chem. Soc.*, 2003, **125**, 9596–9597.

S4. B. Malisova, S. Tosatti, M. Textor, K. Gademann and S. Zürcher, *Langmuir*, 2010, **26**, 4018–4026.

S5. K. Gademann, *ChemBioChem*, 2005, **6**, 913–919.

S6. B. Geiseler and L. Fruk, *J. Mater. Chem.*, 2012, **22**, 735–741.

S7. W. S. Li, D. L. Jiang, Y. Suna and T. Aida, *J. Am. Chem. Soc.*, 2005, **127**, 7700–7702.

7. NMR spectra

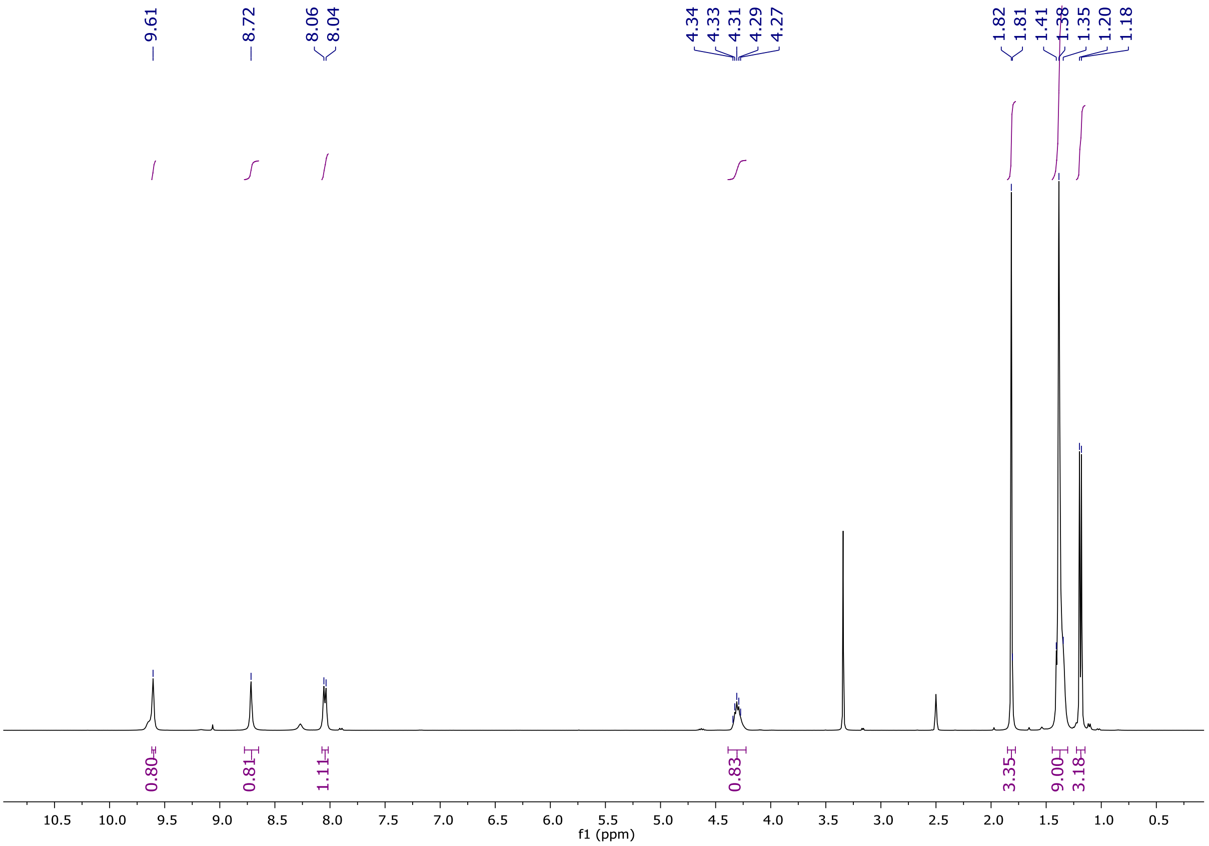

Fig. S26. ^{1}H NMR spectrum of **29** in DMSO-d_6.

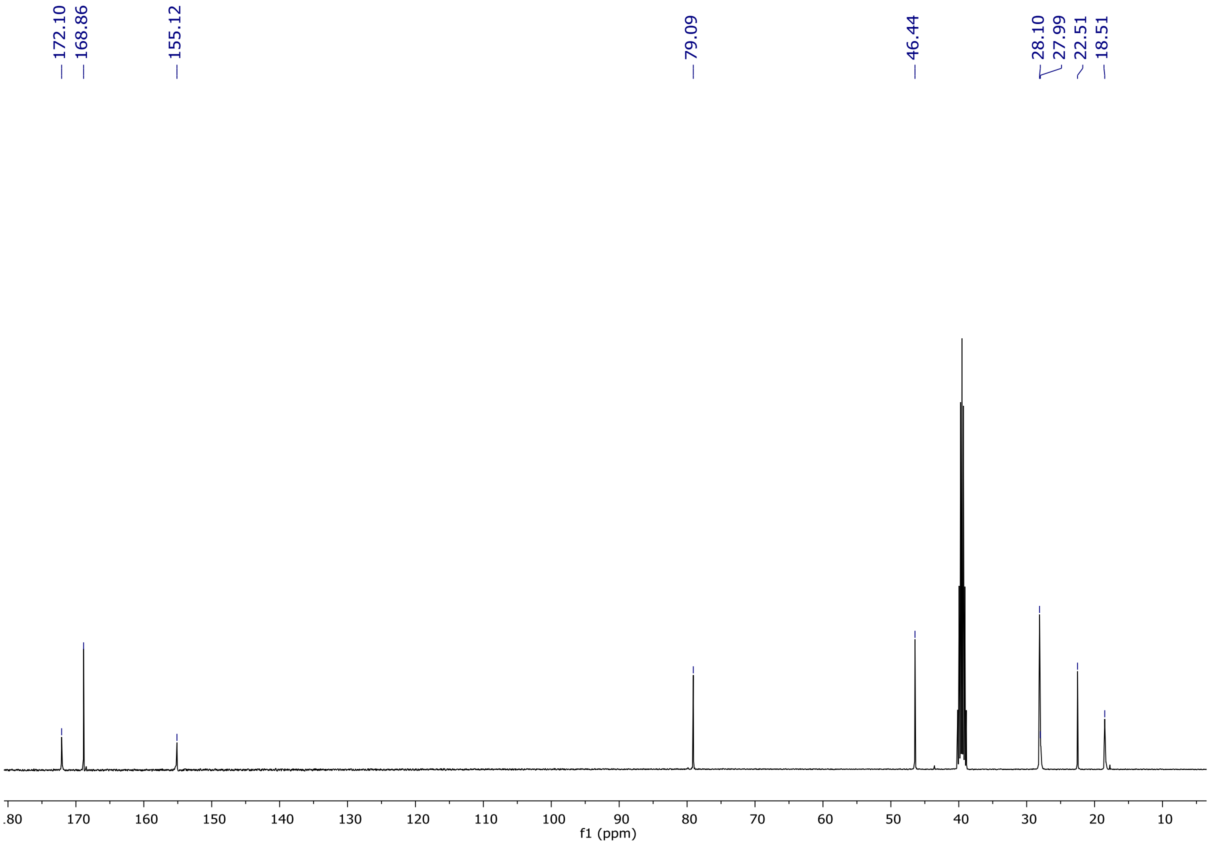

Fig. S27. ^{13}C NMR spectrum of **29** in DMSO-d_6.

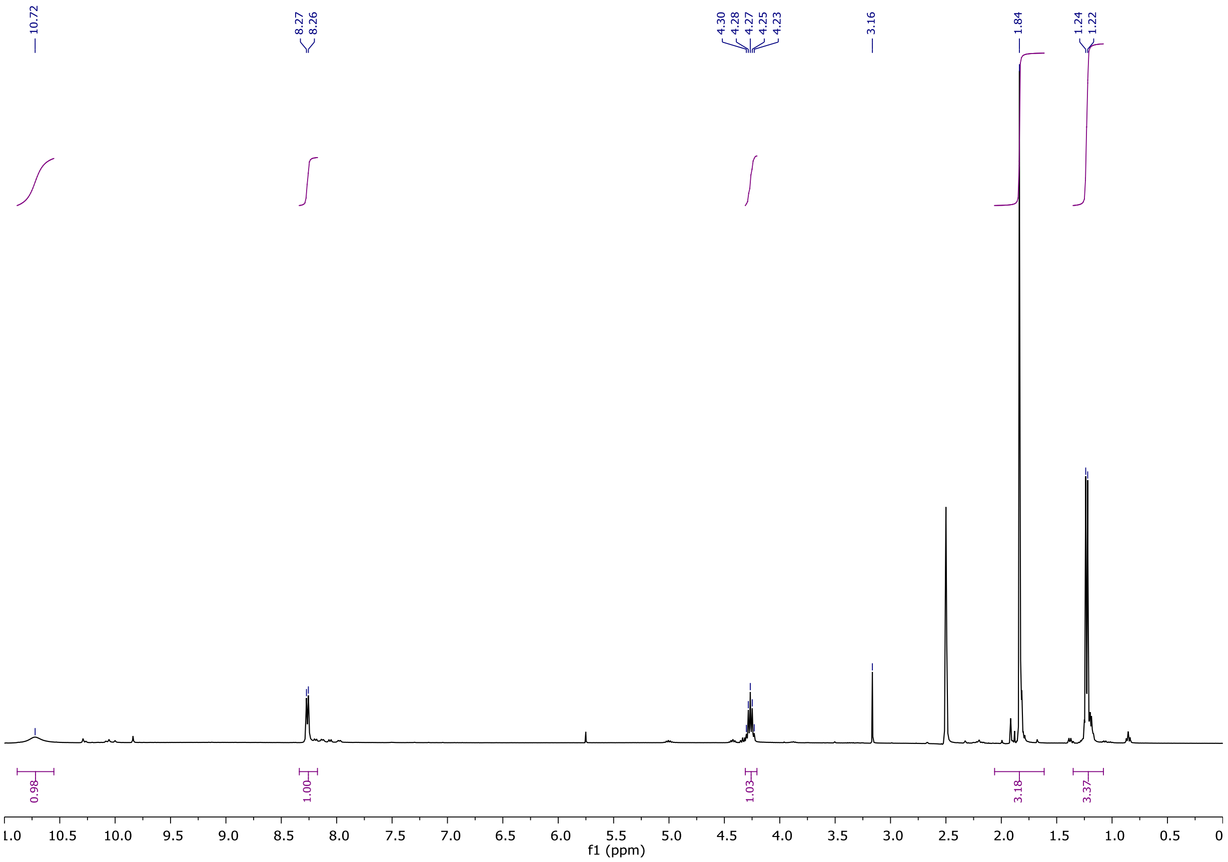

Fig. S28. ^{1}H NMR spectrum of **18** in DMSO-d_6.

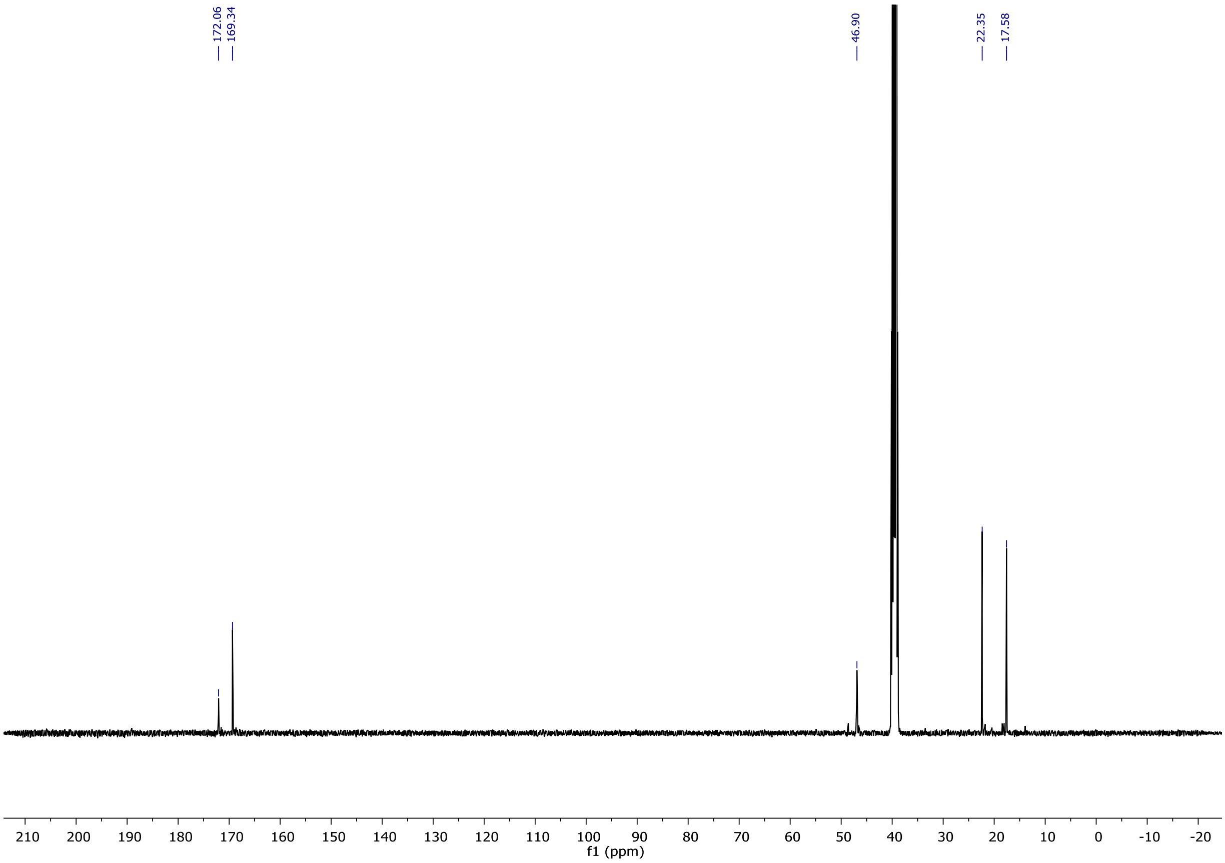

Fig. S29. ^{13}C NMR spectrum of **18** in DMSO-d_6.

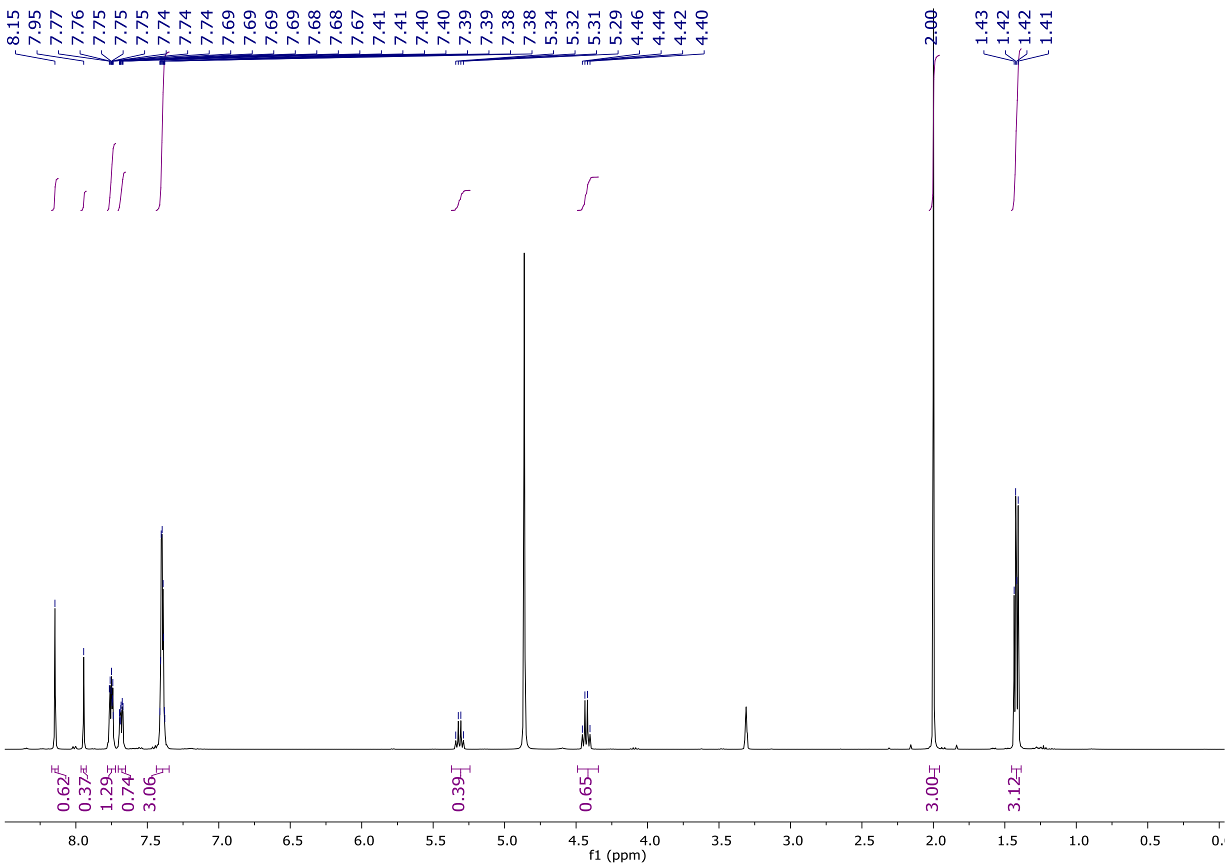

Fig. S30. ^{1}H NMR spectrum of **15** in CD_3OD.

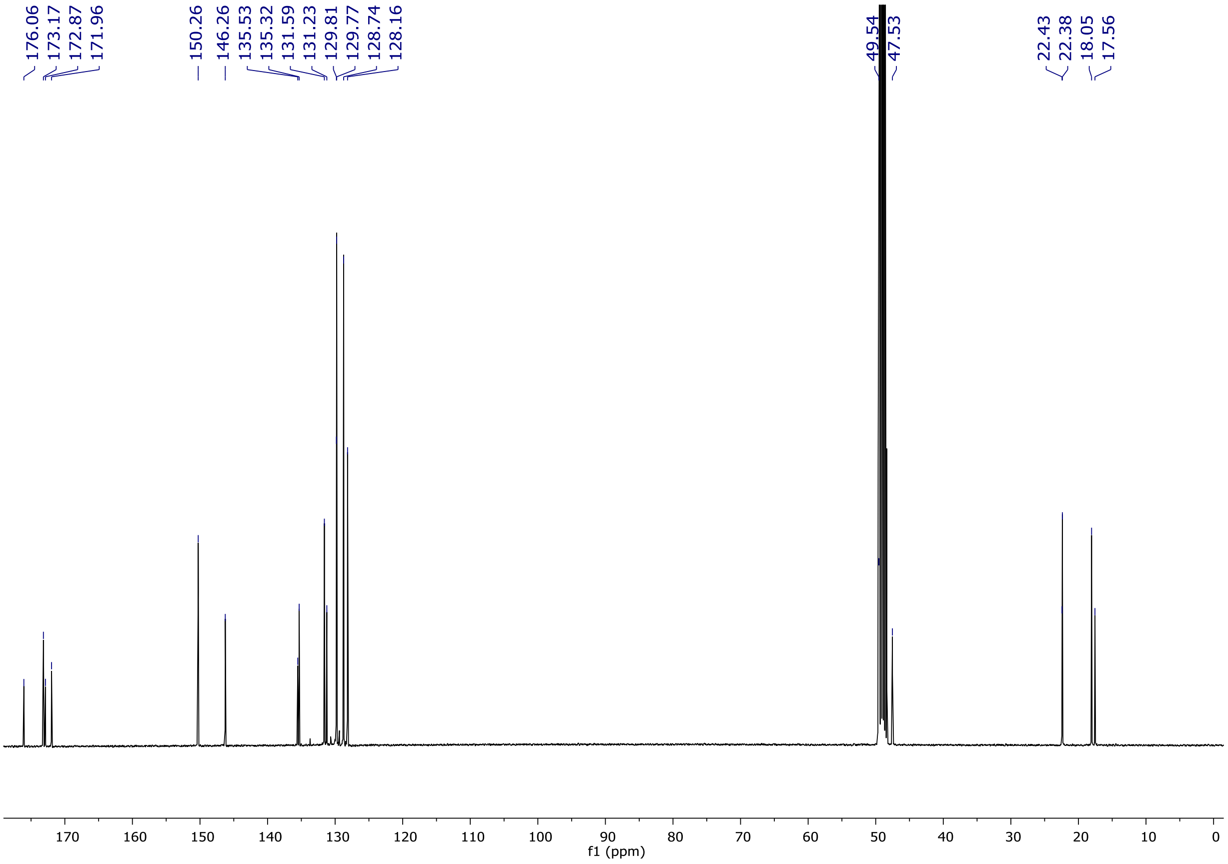

Fig. S31. ^{13}C NMR spectrum of **15** in CD_3OD.

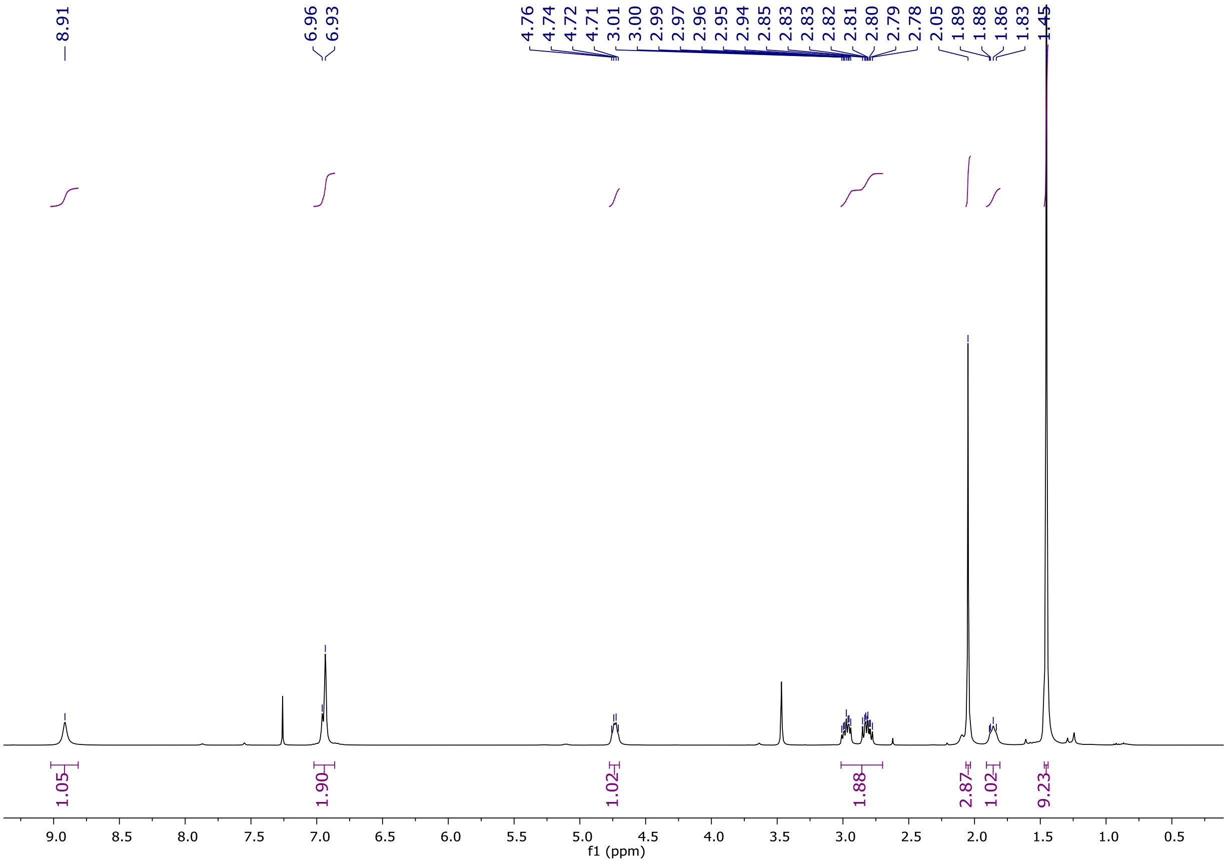

Fig. S32. ^{1}H NMR spectrum of **31** in $CDCl_3$.

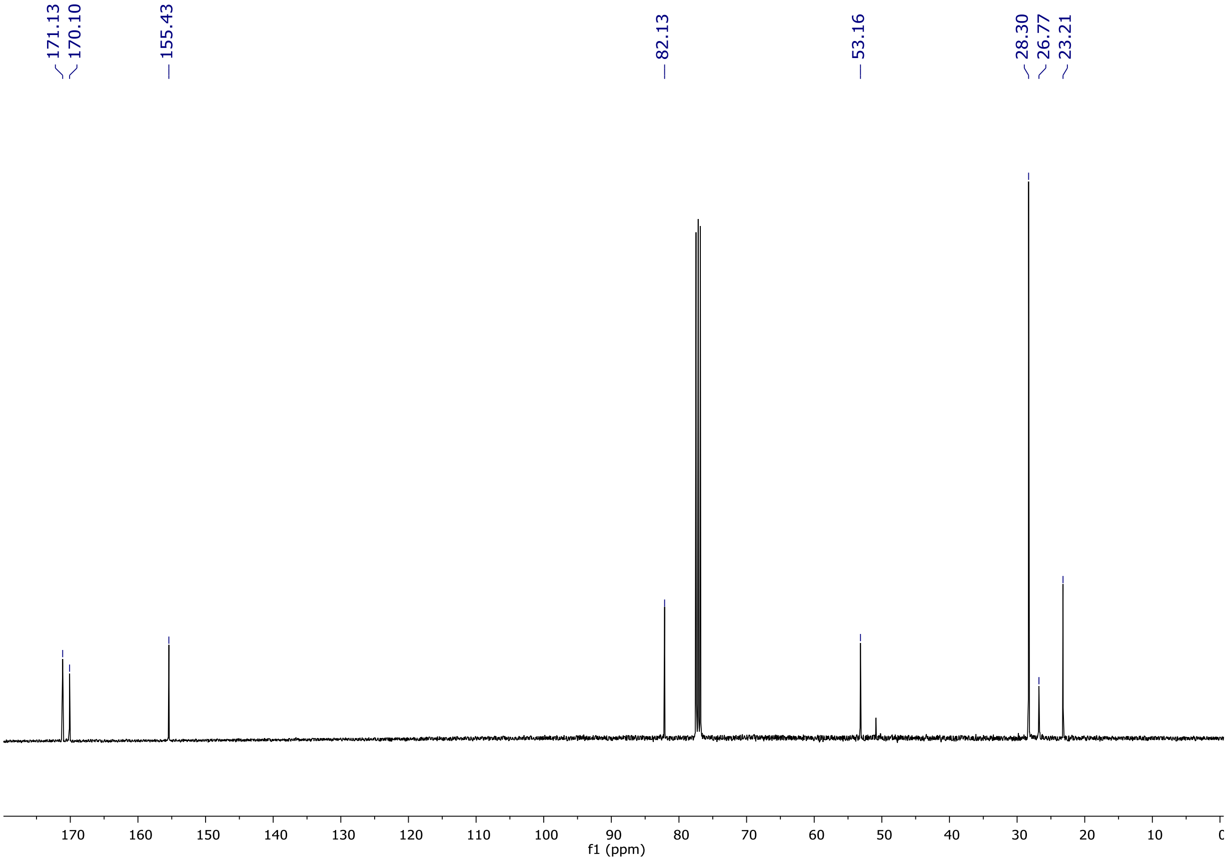

Fig. S33. ^{13}C NMR spectrum of **31** in $CDCl_3$.

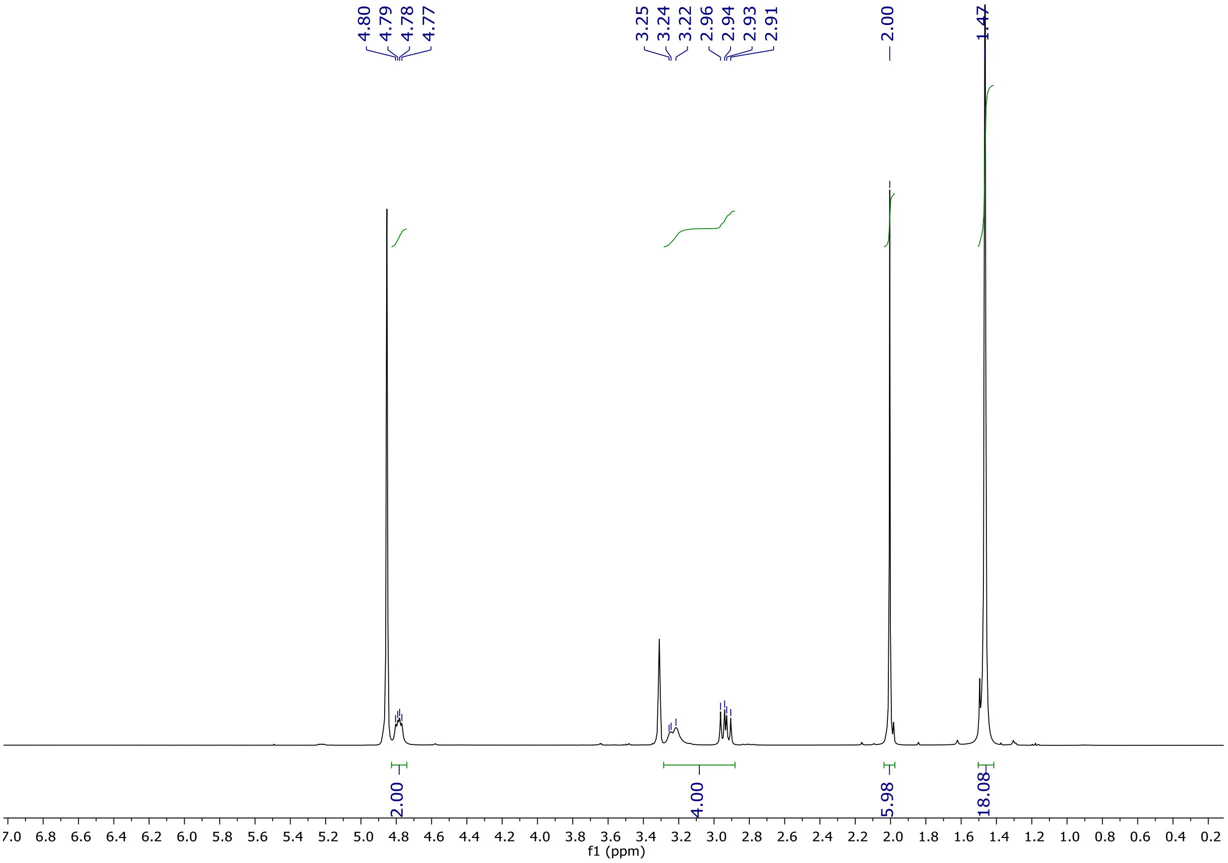

Fig. S34. ^{1}H NMR spectrum of **20** in CD_3OD.

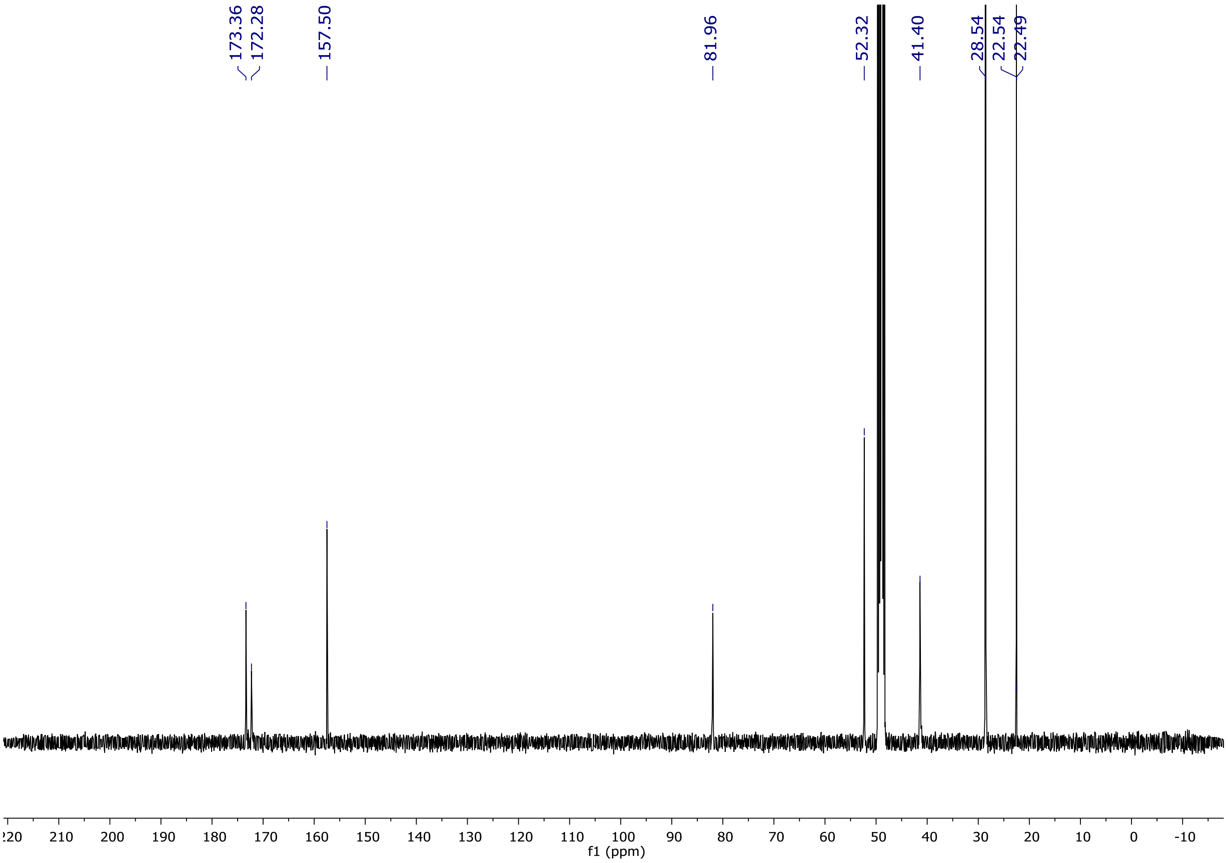

Fig. S35. ^{13}C NMR spectrum of **20** in CD_3OD.

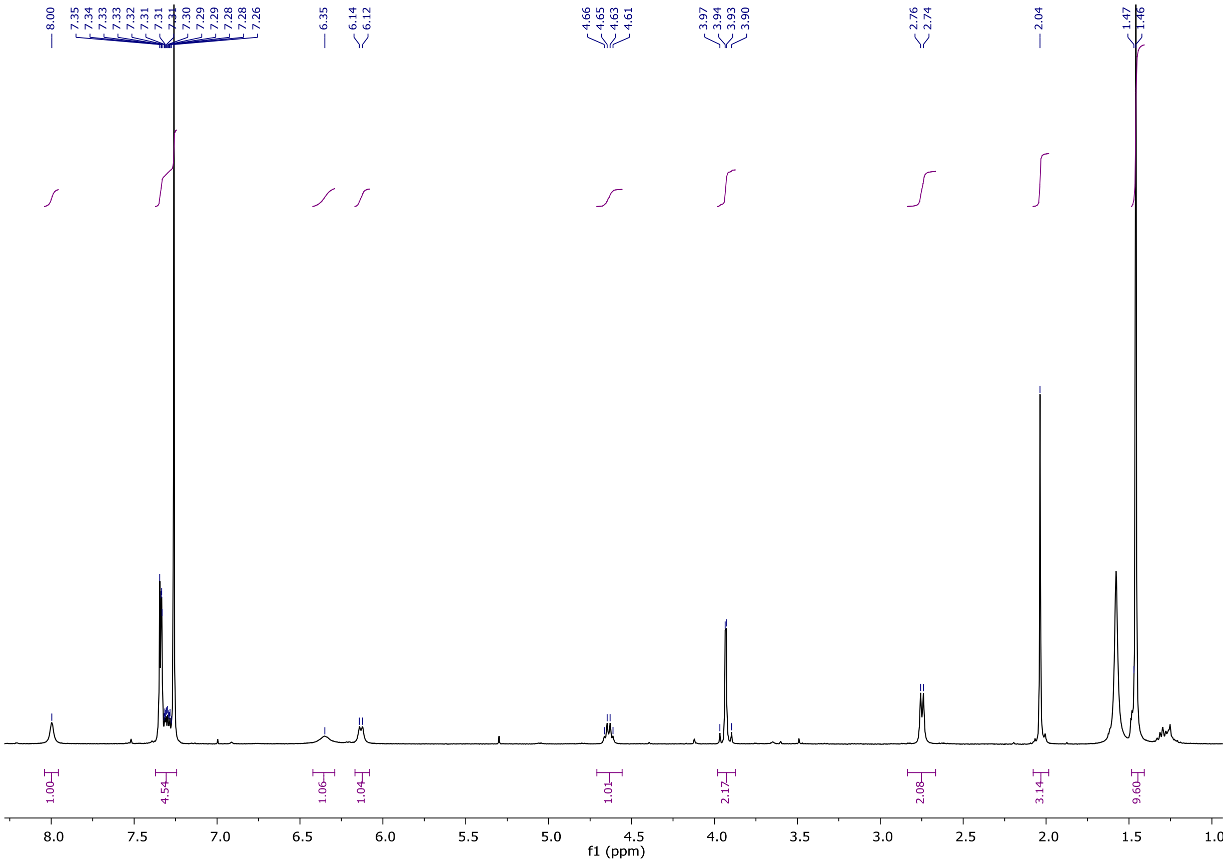

Fig. S36. ^{1}H NMR spectrum of **22** in $CDCl_3$.

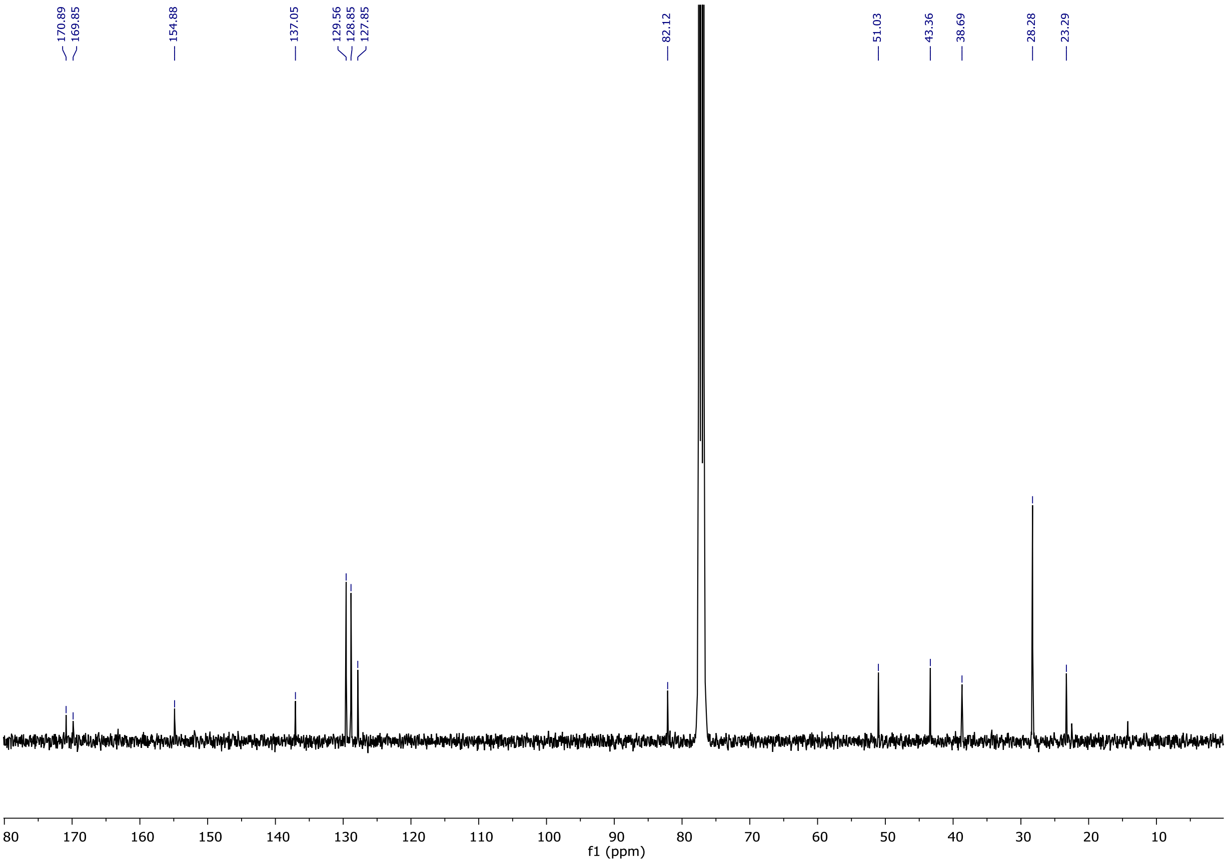

Fig. S37. ^{13}C NMR spectrum of **22** in $CDCl_3$.

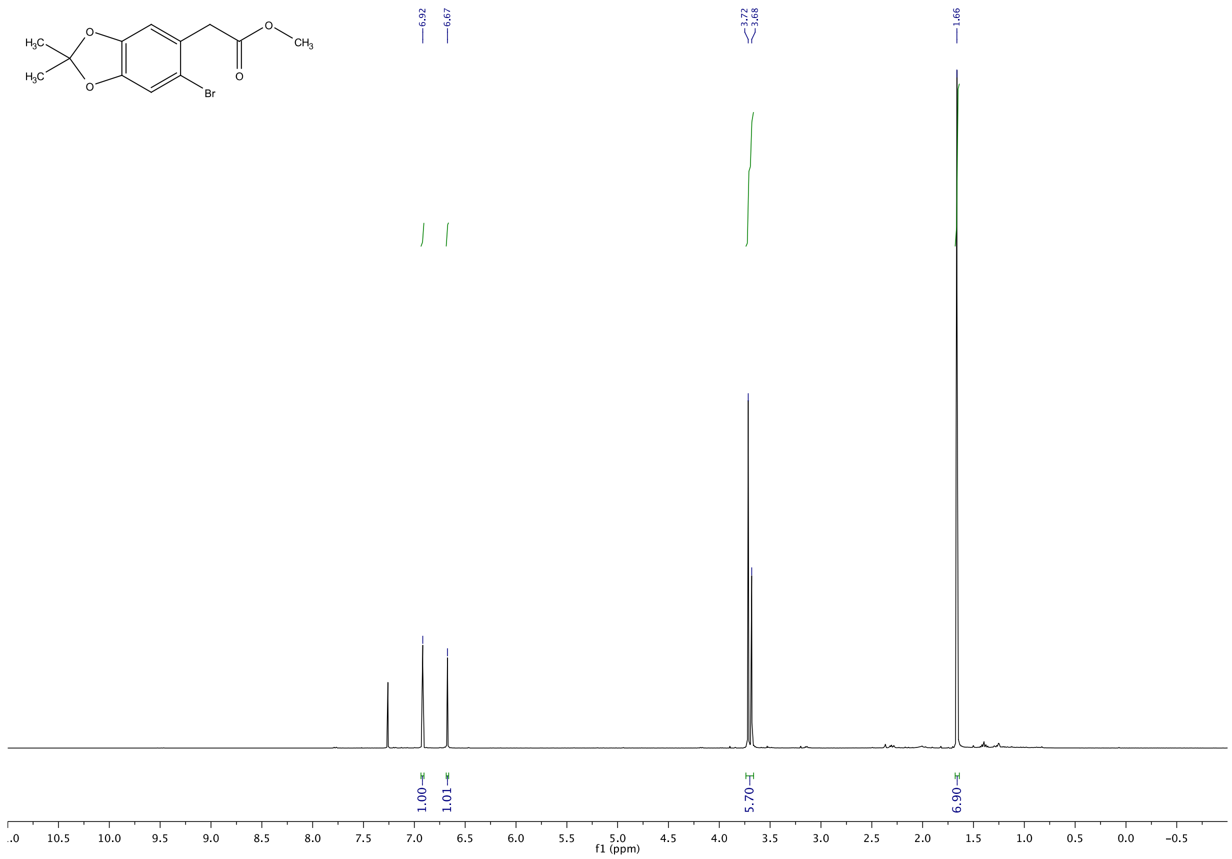

Fig. S38. ^{1}H NMR spectrum of **32** in $CDCl_3$.

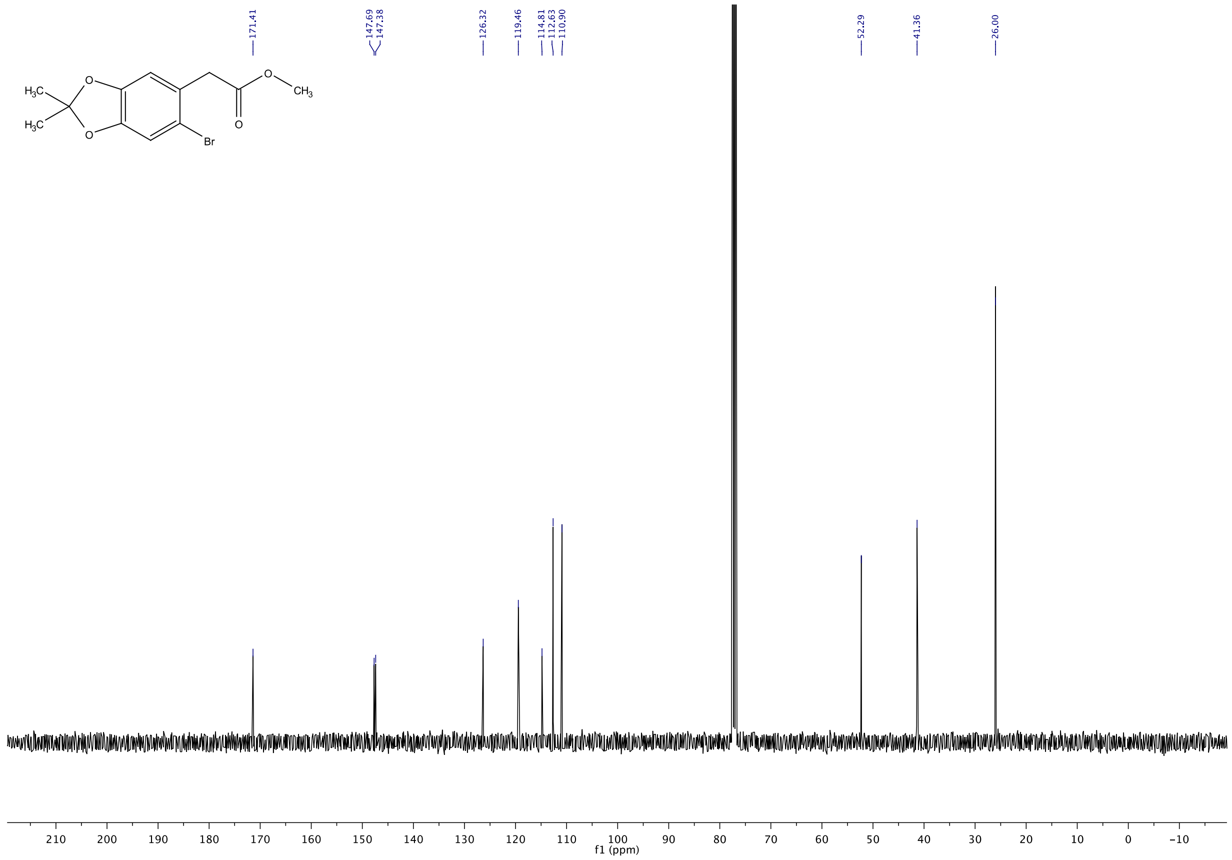

Fig. S39. ^{13}C NMR spectrum of **32** in $CDCl_3$.

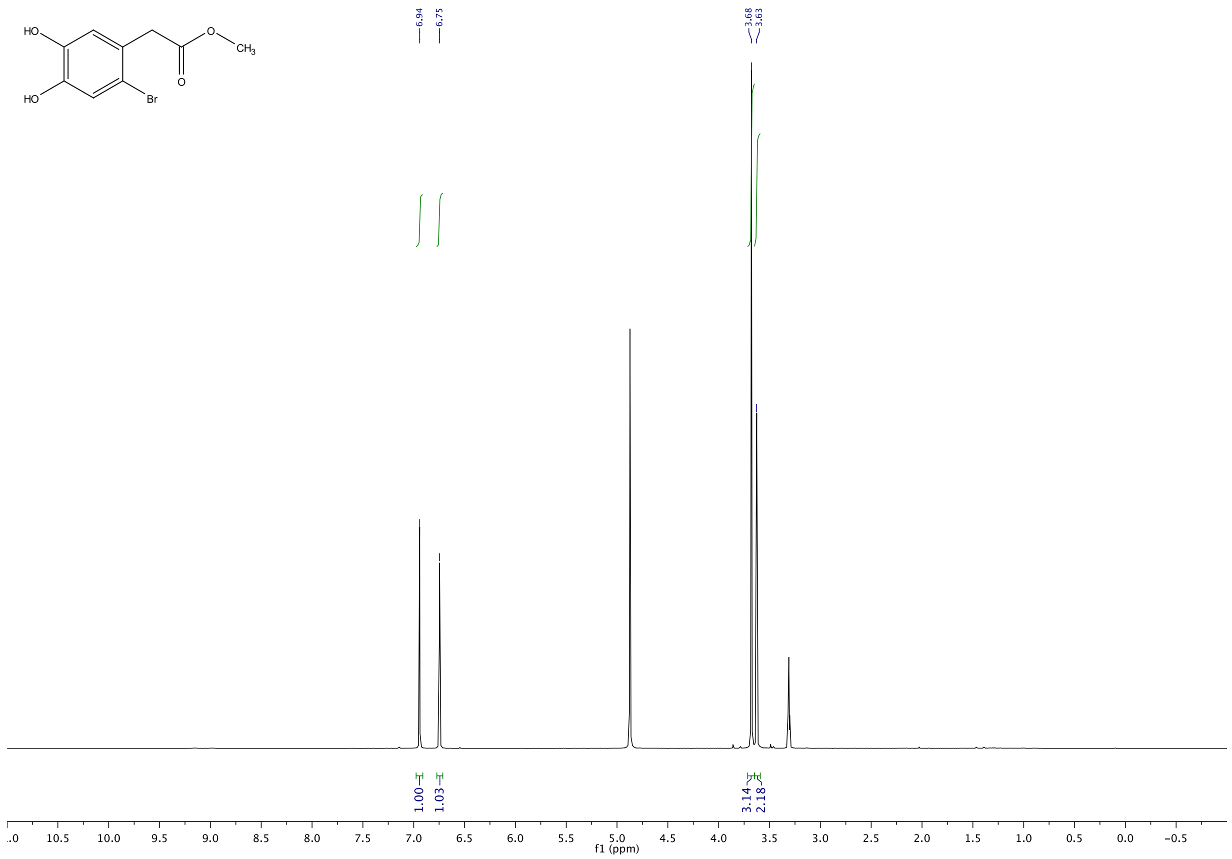

Fig. S40. ^{1}H NMR spectrum of **8** in CD_3OD.

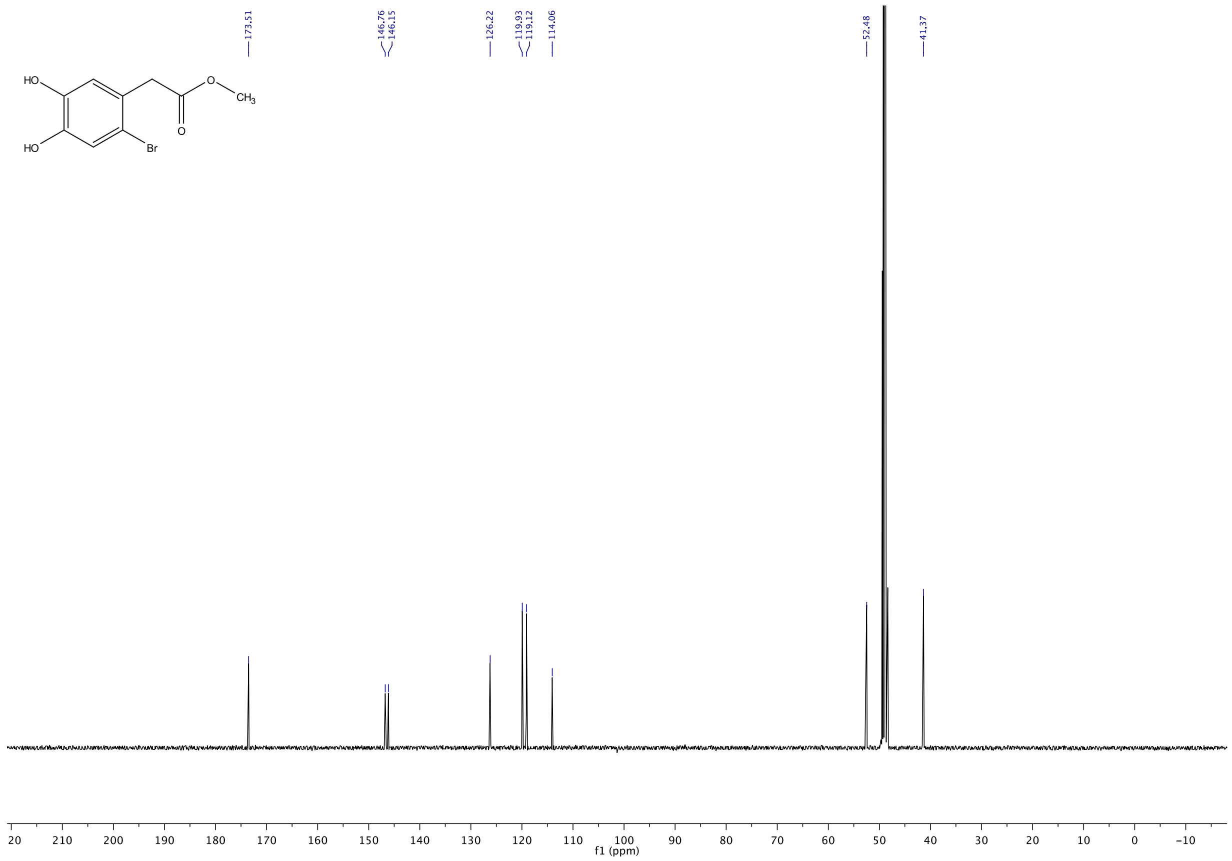

Fig. S41. ^{13}C NMR spectrum of **8** in CD_3OD.

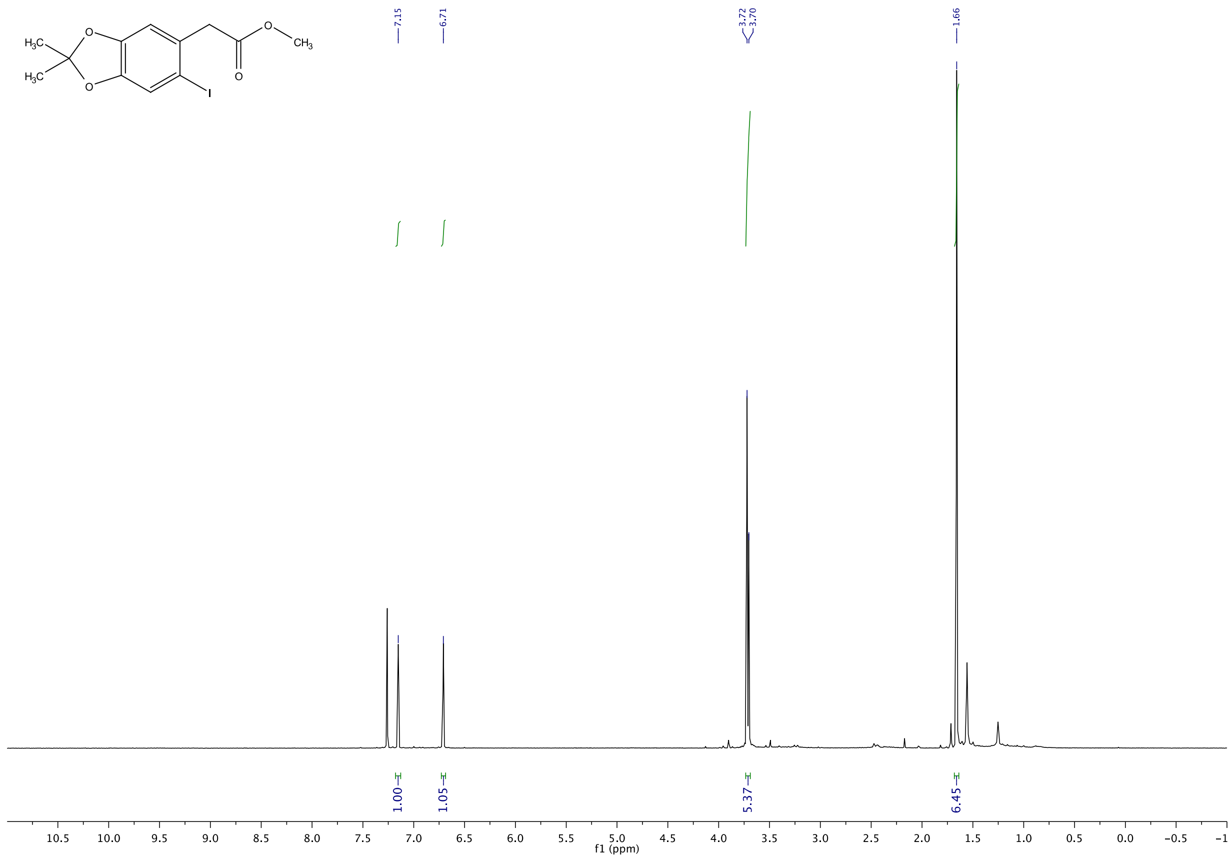

Fig. S42. ^{1}H NMR spectrum of **33** in $CDCl_3$.

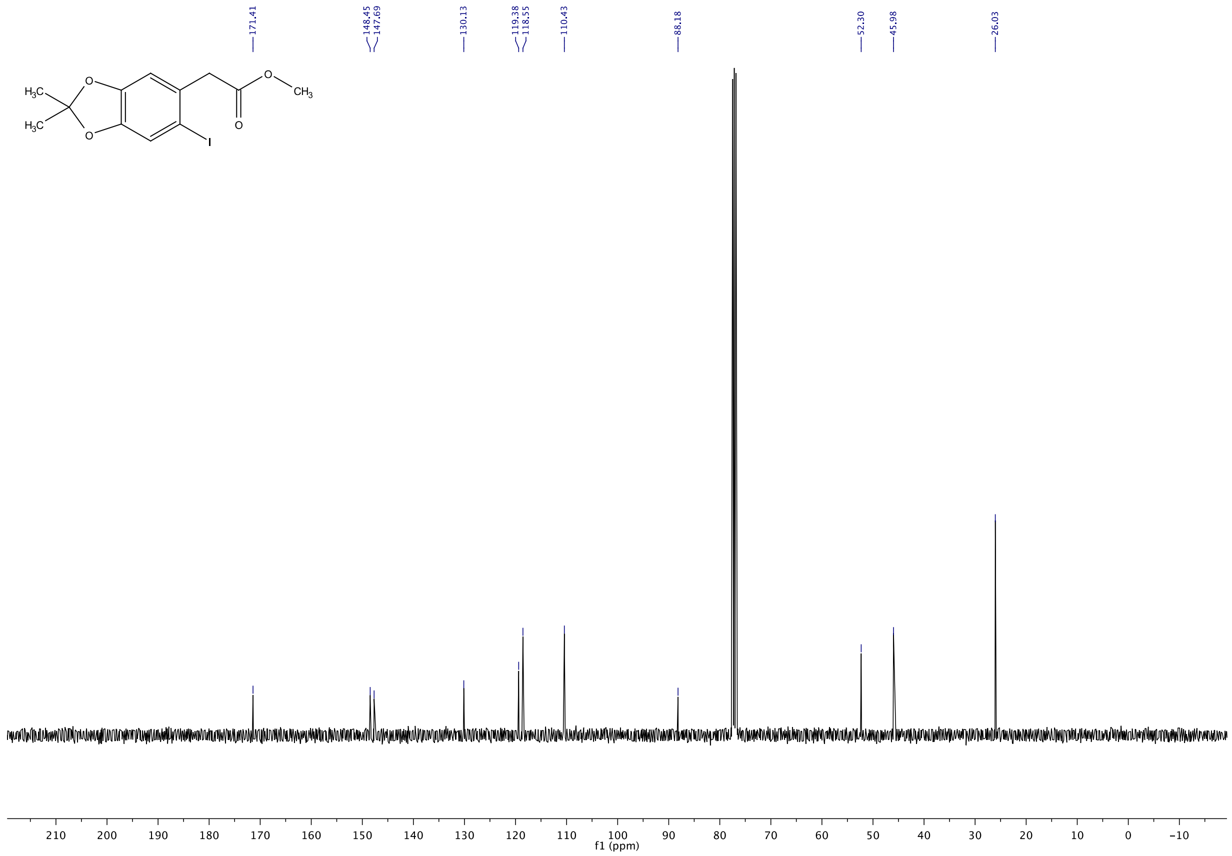

Fig. S43. ^{13}C NMR spectrum of **33** in $CDCl_3$.

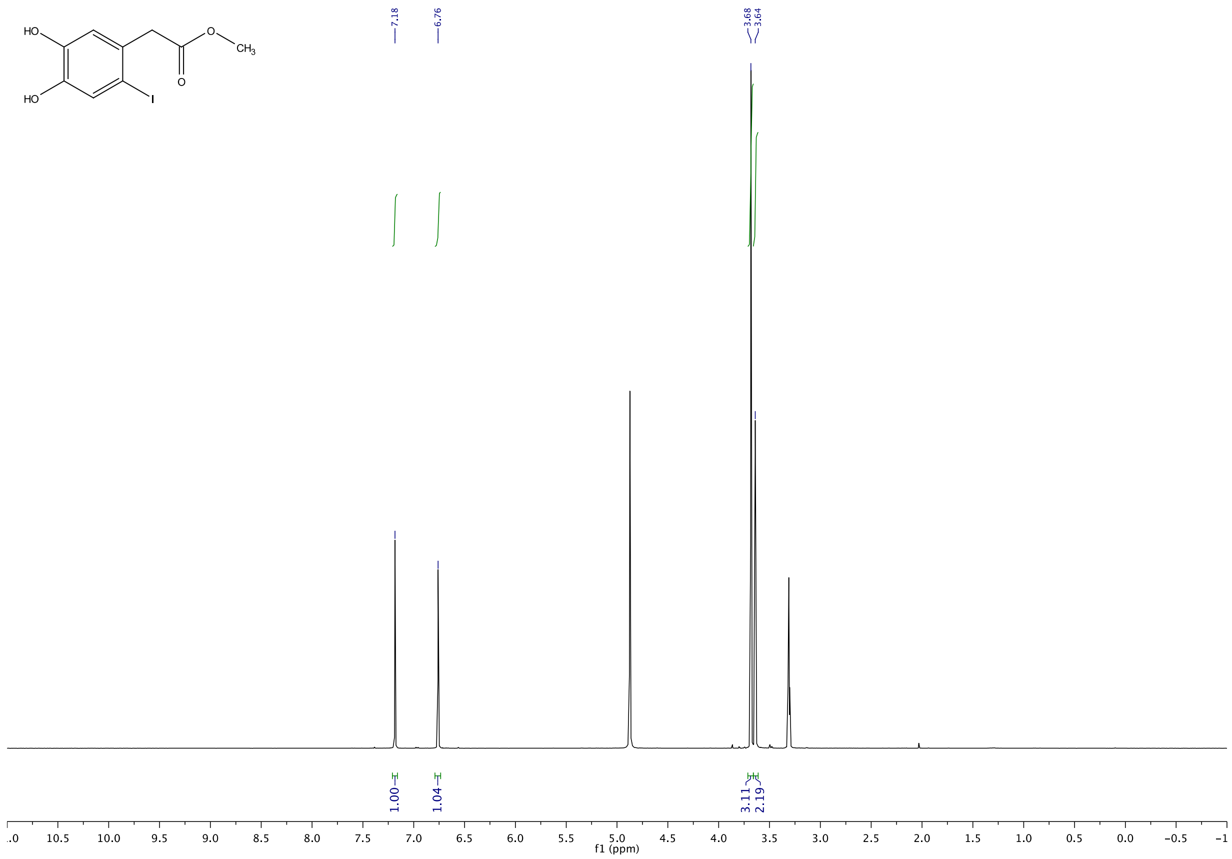

Fig. S44. ^{1}H NMR spectrum of **9** in CD_3OD.

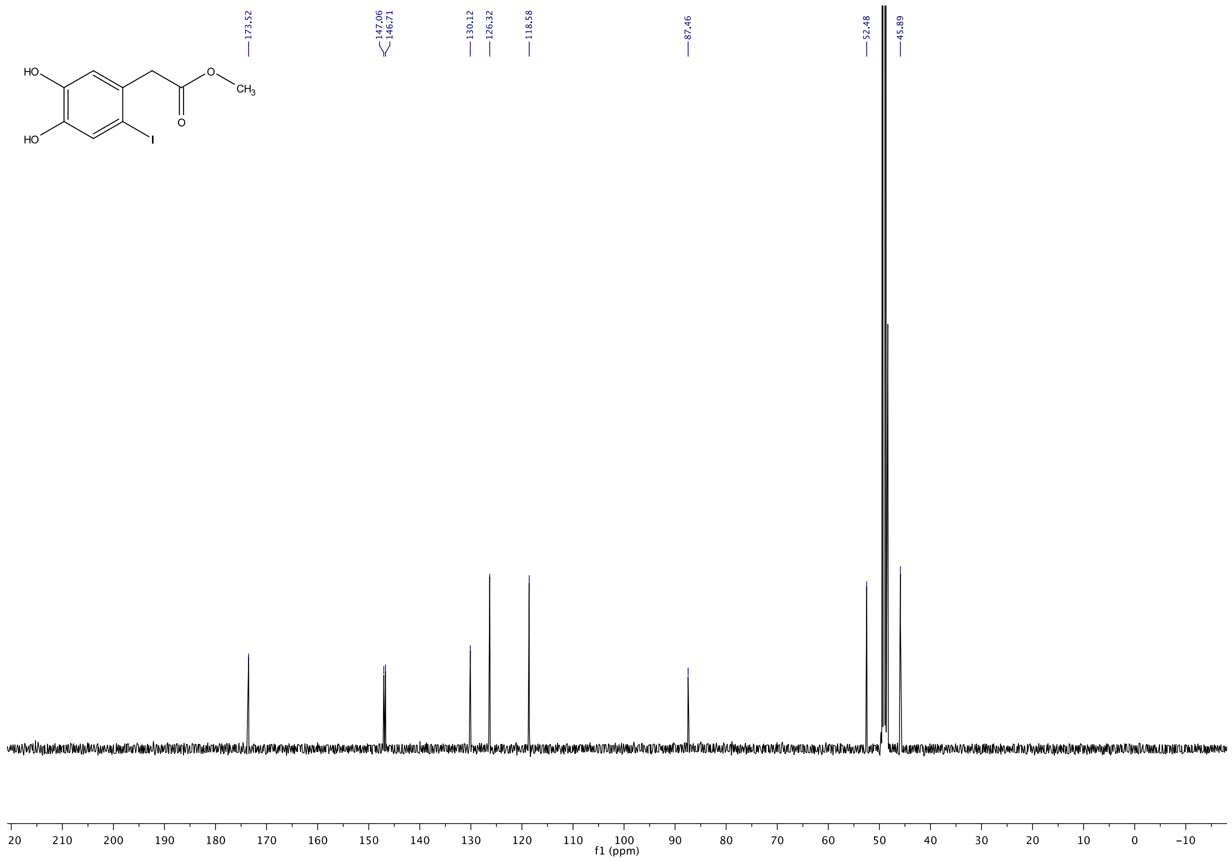

Fig. S45. ^{13}C NMR spectrum of **9** in CD_3OD.

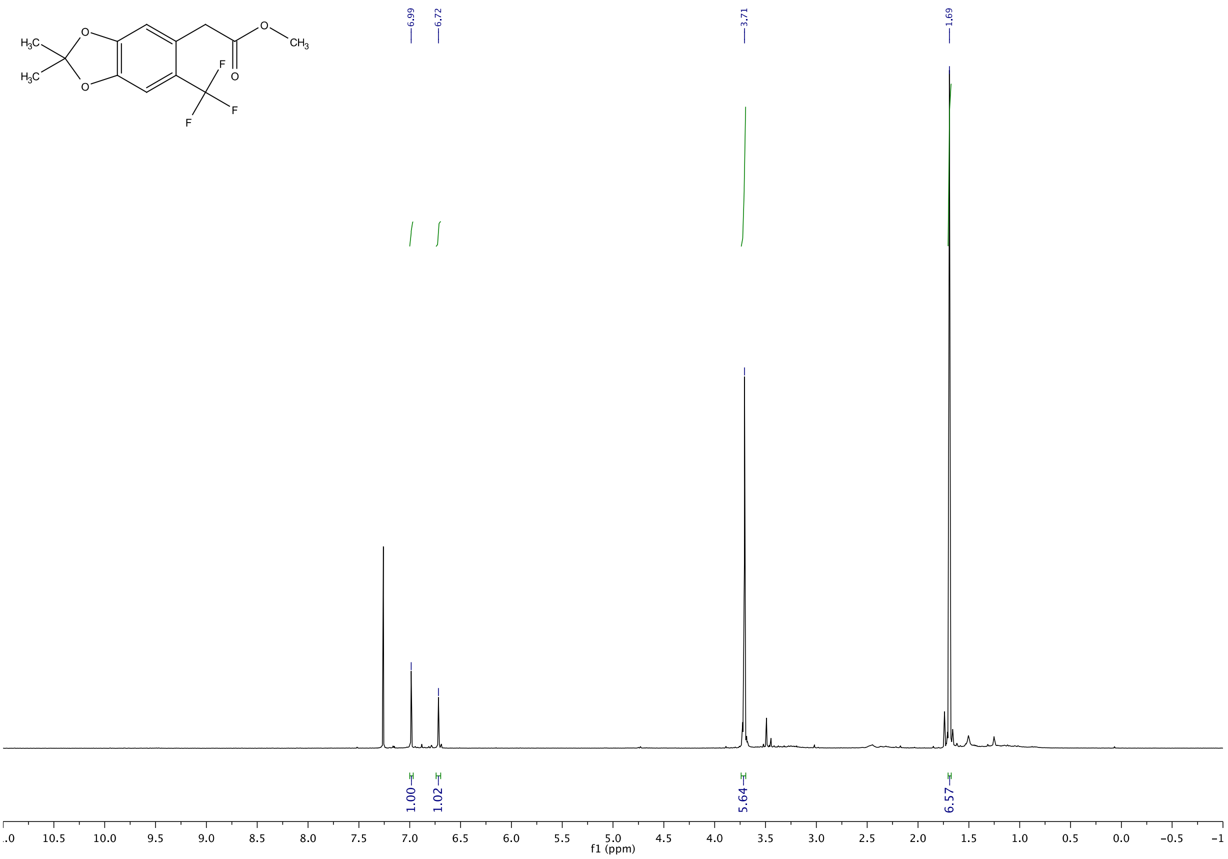

Fig. S46. ^{1}H NMR spectrum of **34** in $CDCl_3$.

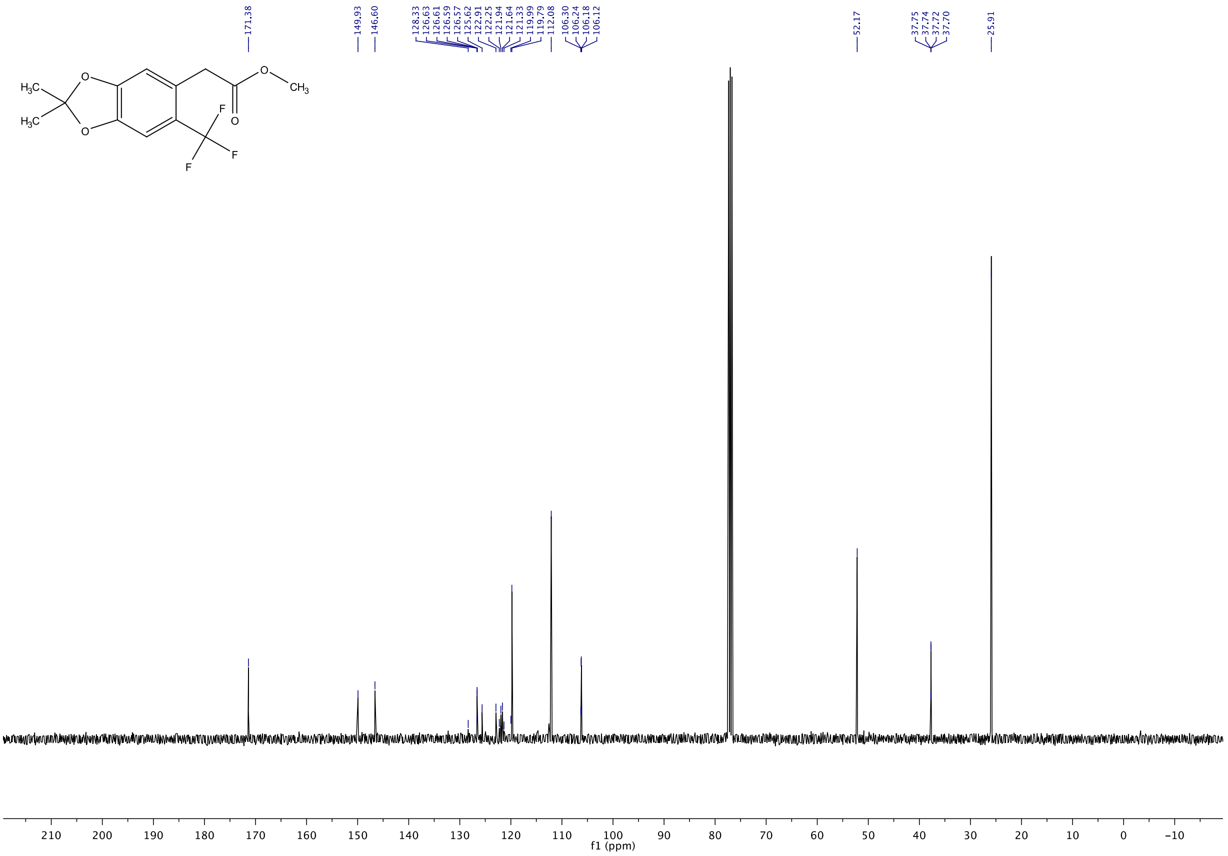

Fig. S47. ^{13}C NMR spectrum of **34** in $CDCl_3$.

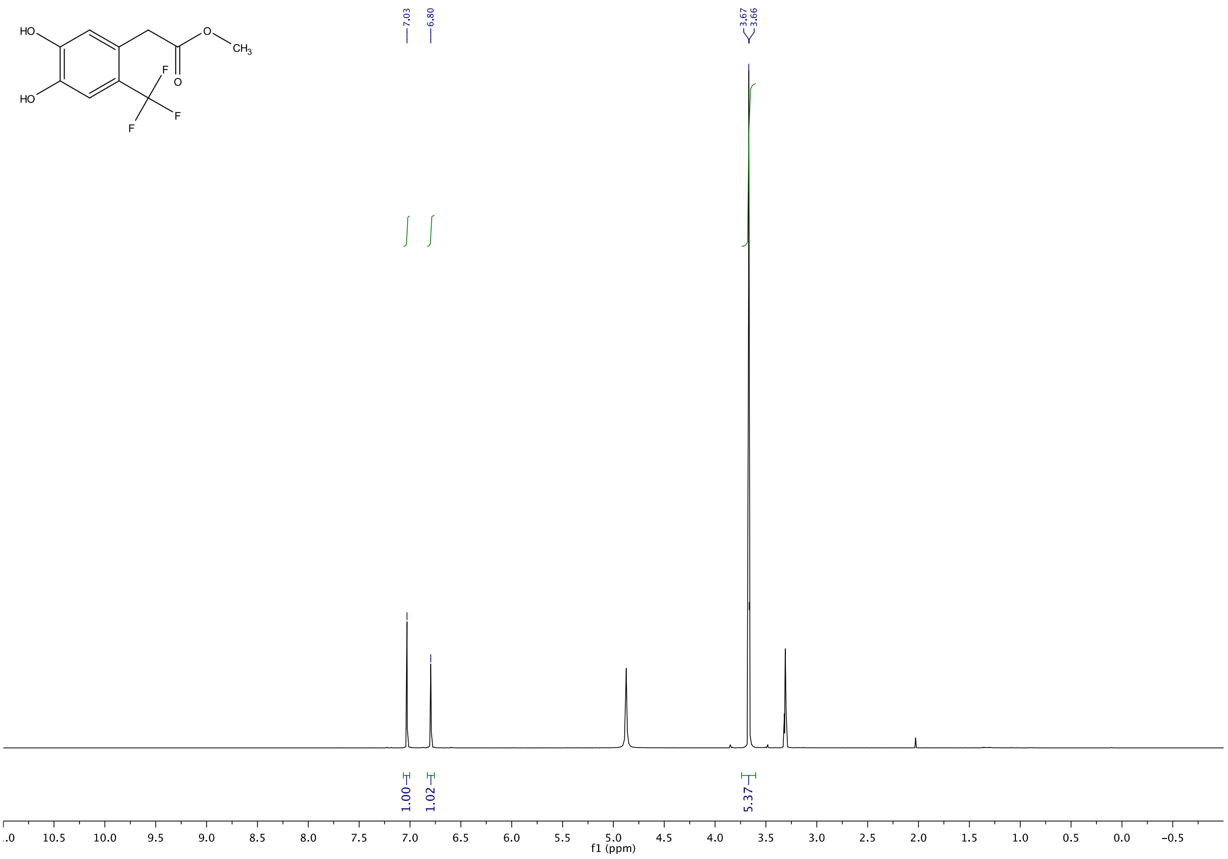

Fig. S48. ^{1}H NMR spectrum of **7** in CD_3OD.

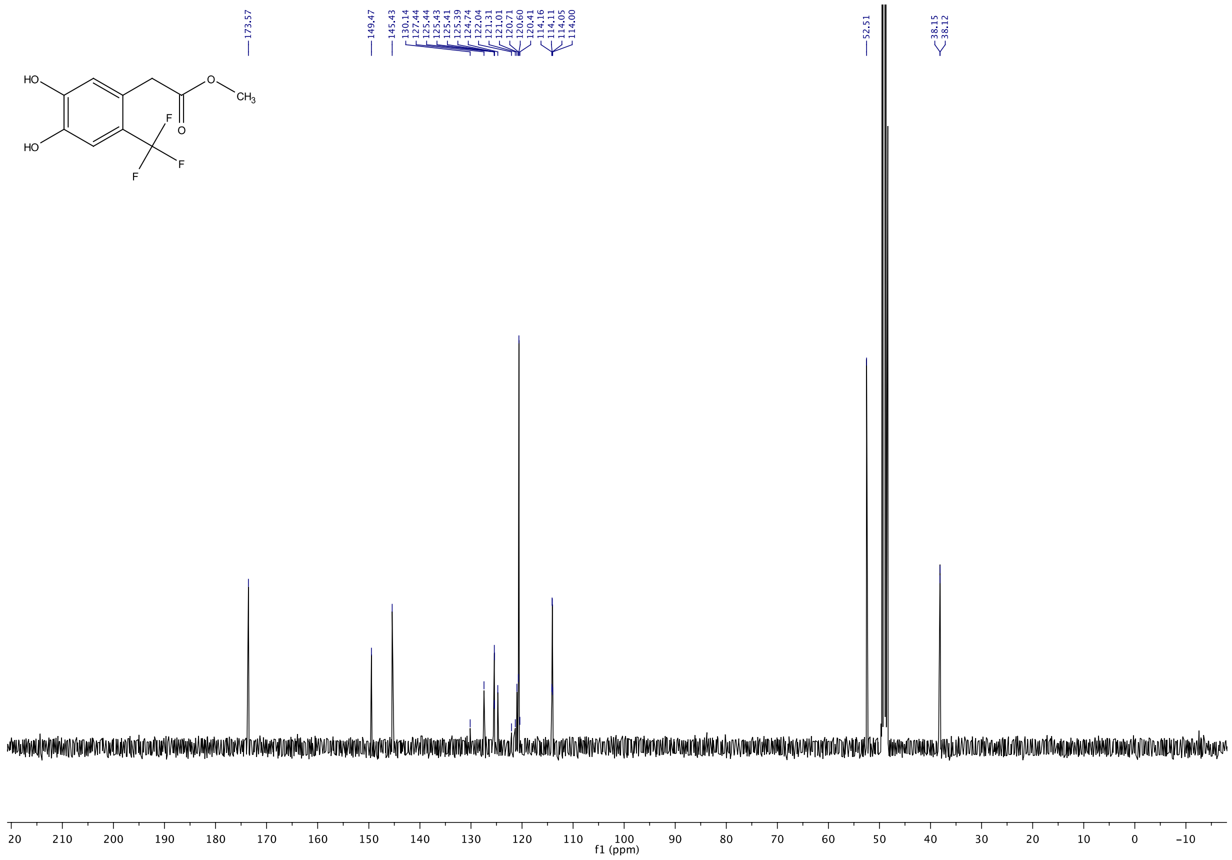

Fig. S49. ^{13}C NMR spectrum of **7** in CD_3OD.

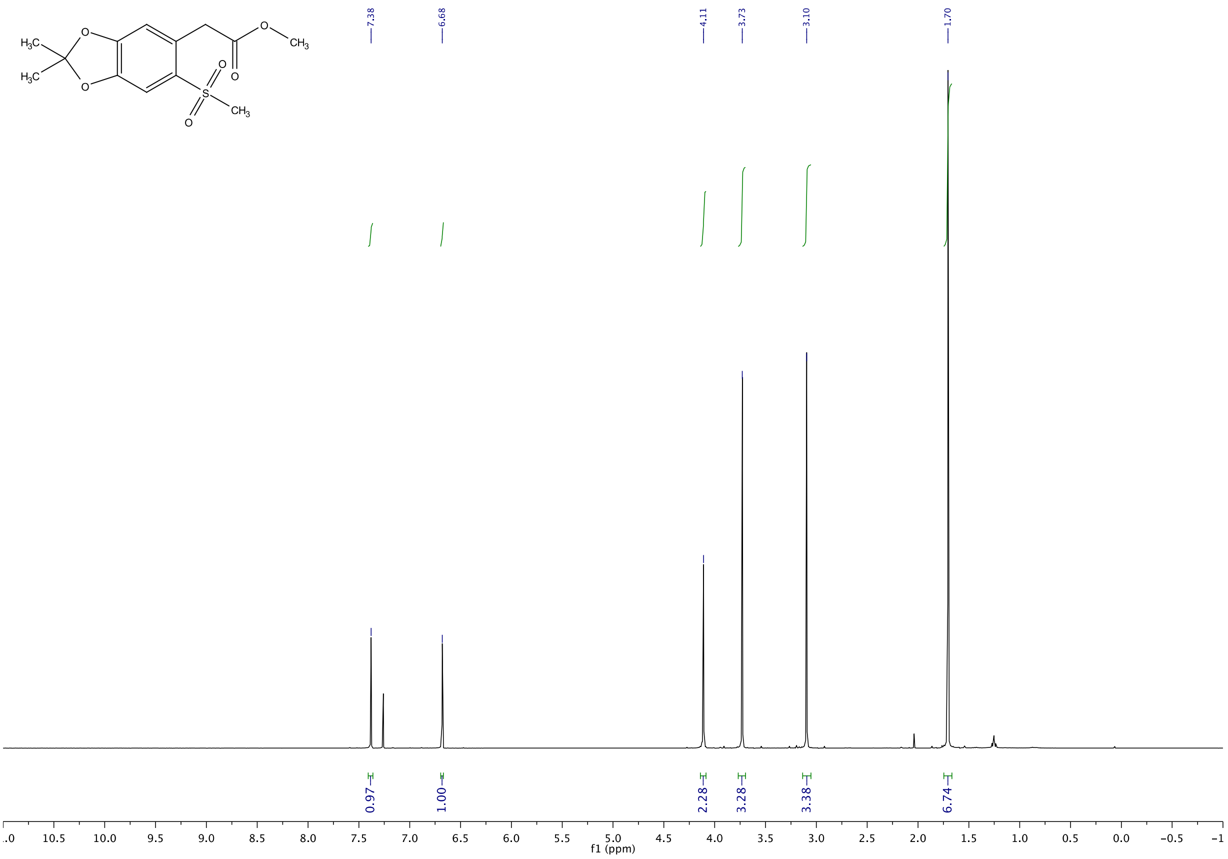

Fig. S50. ^{1}H NMR spectrum of **35** in $CDCl_3$.

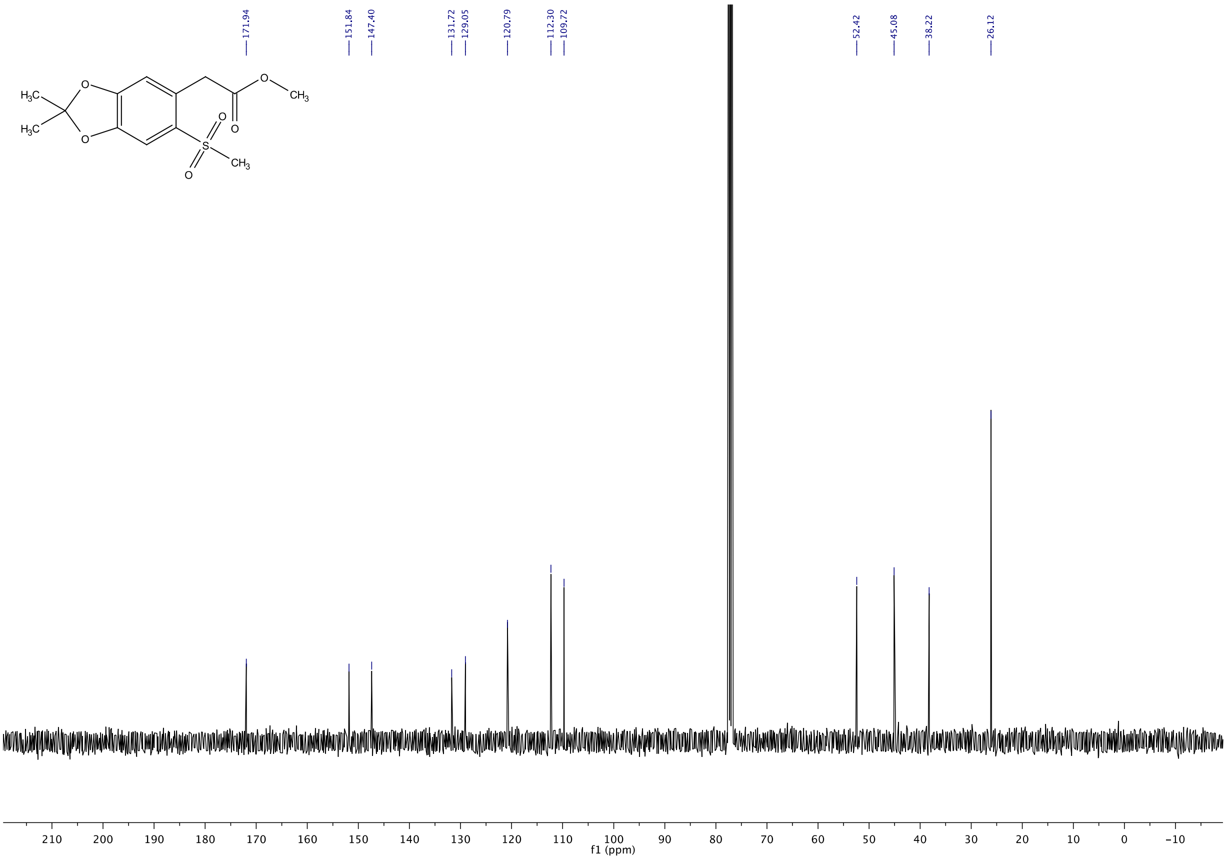

Fig. S51. ^{13}C NMR spectrum of **35** in $CDCl_3$.

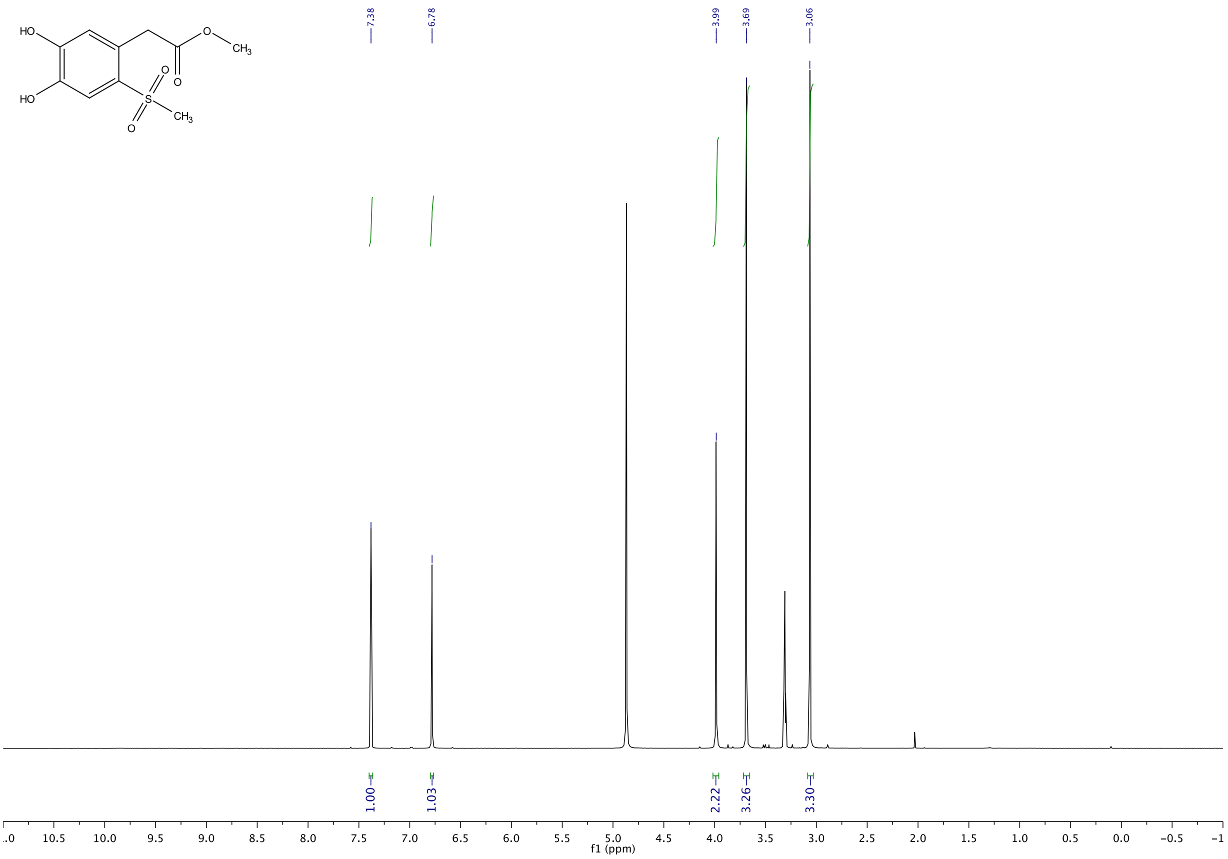

Fig. S52. ^{1}H NMR spectrum of **2** in CD_3OD.

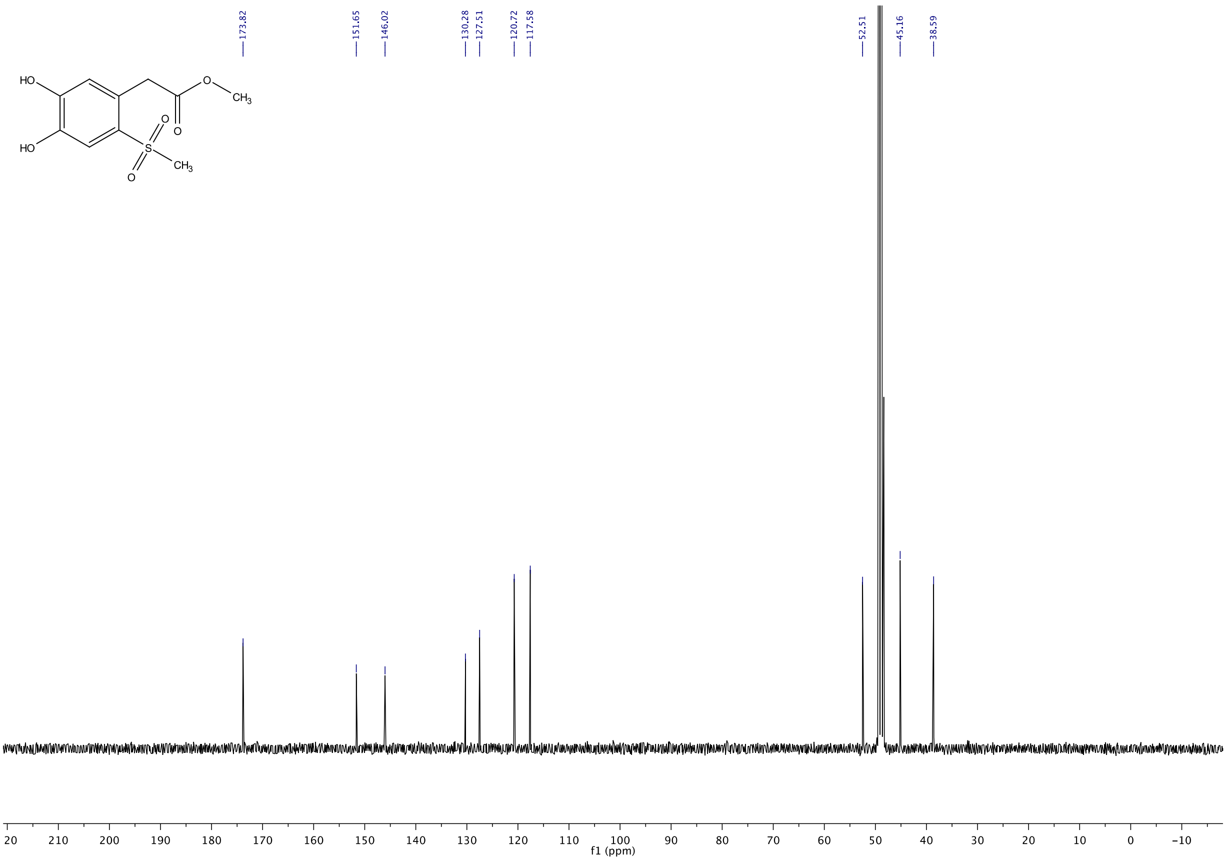

Fig. S53. ^{13}C NMR spectrum of **2** in CD_3OD.
